# Synthesis and Antimicrobial Activity of 1,2-Benzothiazine Derivatives

**DOI:** 10.3390/molecules21070861

**Published:** 2016-06-30

**Authors:** Chandani Patel, Jatinder P. Bassin, Mark Scott, Jenna Flye, Ann P. Hunter, Lee Martin, Madhu Goyal

**Affiliations:** 1 School of Life and Medical Sciences, University of Hertfordshire, Hatfield AL10 9AB, UK; chandanipatel21@hotmail.com (C.P.); m.scott6@herts.ac.uk (M.S.); 2 EPSRC UK National Mass Spectrometry Facility, Institute of Mass Spectrometry, Swansea University Medical School, Swansea SA2 8PP, UK; mscentre@swansea.ac.uk (J.F.); a.p.hunter@swansea.ac.uk (A.P.H.); 3 School of Science and Technology, Nottingham Trent University, Clifton Lane, Clifton, Nottingham NG11 8NS, UK; lee.martin@ntu.ac.uk

**Keywords:** 1,2-benzothiazines, chalcones, *Bacillus subtilis*, *Staphylococcous aureus*, *Proteus vulgaris*, *Salmonella typhimurium*

## Abstract

A number of 1,2-benzothiazines have been synthesized in a three-step process. Nine chalcones **1**–**9** bearing methyl, fluoro, chloro and bromo substituents were chlorosulfonated with chlorosulfonic acid to generate the chalcone sulfonyl chlorides **10**–**18**. These were converted to the dibromo compounds **19**–**27** through reaction with bromine in glacial acetic acid. Compounds **19**–**27** were reacted with ammonia, methylamine, ethylamine, aniline and benzylamine to generate a library of 45 1,2-benzothiazines **28**–**72**. Compounds **28**–**72** were evaluated for their antimicrobial activity using broth microdilution techniques against two Gram-positive bacteria (*Bacillus subtilis* and *Staphylococcus aureus*) and two Gram-negative bacteria (*Proteus vulgaris* and *Salmonella typhimurium*). The results demonstrated that none of the compounds showed any activity against Gram-negative bacteria *P. vulgaris* and *S. typhimurium*; however, compounds **31**, **33**, **38**, **43**, **45**, **50**, **53**, **55**, **58**, **60**, **63** and **68** showed activity against Gram-positive bacteria *Bacillus subtilis* and *Staphylococcous aureus*. The range of minimum inhibitory concentration (MIC) and minimum bactericidal concentration (MBC) was 25–600 µg/mL, though some of the MIC and MBC concentrations were high, indicating weak activity. Structure activity relationship studies revealed that the compounds with a hydrogen atom or an ethyl group on the nitrogen of the thiazine ring exerted antibacterial activity against Gram-positive bacteria. The results also showed that the compounds where the benzene ring of the benzoyl moiety contained a methyl group or a chlorine or bromine atom in the *para* position showed higher antimicrobial activity. Similar influences were identified where either a bromine or chlorine atom was in the *meta* position.

## 1. Introduction

The emergence of drug-resistant strains of bacteria is an increasing threat to society. Many antibiotics that were formerly effective in curing bacterial infections are no longer efficient because of the evolution of resistant strains. Therefore, there is an urgent need for a new class of antibacterial agents [1]. Clinically, antibiotic resistance is one of the greatest challenges of the 21st century. Antibiotic resistance has led to the emergence of superbugs such as methicillin-resistant *Staphylococcus aureus* and extremely resistant tuberculosis bacteria which are impossible to treat with available medicines [2,3]. New antibacterial agents are currently being developed at a much slower pace than our growing need for such drugs. Amongst the family of heterocyclic compounds, those with nitrogen and sulfur atoms have been identified as having the most comprehensive spectrum of biological activities [4]. The biological and pharmacological properties of these compounds have been demonstrated in a number of studies [4,5].

**Figure 1:**
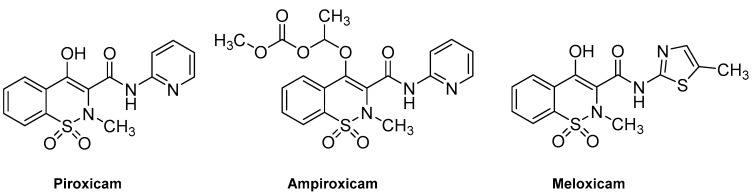
Structures of 1,2-benzothiazine-based drugs.

**Scheme 1:**
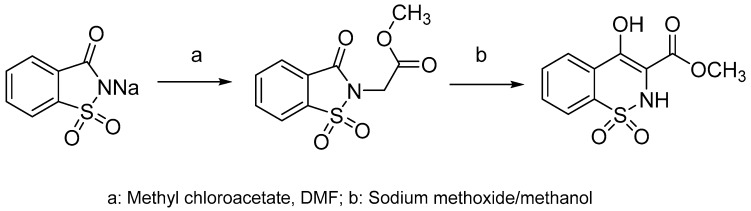
Synthesis of 1,2-benzothiazines via the alkoxide rearrangement of saccharin derivatives.

Derivatives of benzothiazine have, depending upon the substituents present, shown biological activities that range from antipsychotic to anti-inflammatory [6,7,8]. The 1,2-benzothiazines have shown various biological activities such as anti-inflammatory [9], CNS depressant [10,11], anti-depressants [12], anticancer [13] and antimicrobial [14]. They also act as potent calpain I inhibitors [15] and vasodilators [16]. Previous research has shown 1,2-benzothiazines to be potent antimicrobial agents against Gram-positive bacterial strains and fungal strains such as *Aspergillus flavus*, *Aspergillus niger*, *Fusarium oxysporum* [17]. In addition 1,2-benzothiazines have been seen to show marked activity against *B. subtilis* [17,18,19,20]. In recent years there has been a rapid growth in the literature pertaining to the benzothiazine ring system; much of this literature relates to the discovery that 3-carboxamides of 2-alkyl-4-hydroxy-2*H*-1,2-benzothiazine-1,1-dioxides are anti-inflammatory agents, which includes the drugs piroxicam, ampiroxicam and meloxicam (Figure 1) [21,22,23,24,25,26,27,28]. The common route to these types of compounds is via the alkoxide rearrangement of saccharin derivatives (Scheme 1) [29,30,31,32,33,34,35].

There are a number of different synthetic routes to 1,2-benzothiazines; however, the starting materials are not readily available. We recently developed a straightforward route to 1,2-benzothiazines using readily available chalcones [36]. In light of these observations, a series of new 1,2-benzothiazines were synthesized with the aim of obtaining potential antibacterial agents.

## 2. Results

### 2.1. Chemistry

**Scheme 2:**
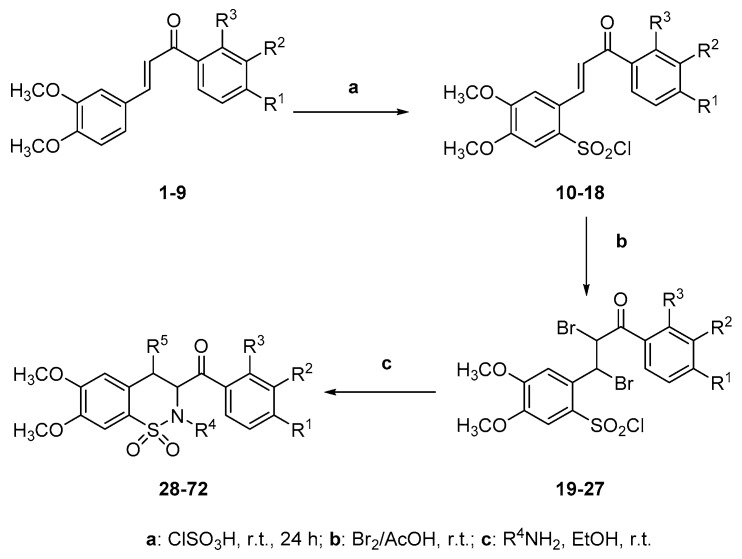
Synthetic steps for compounds **10**–**18**, **19**–**27** and 1,2-benzothiazines **28**–**72**.

Synthetic target compounds **28**–**72** were synthesized in a three-step process outlined in Scheme 2 in yields of 44%–96%. The chalcones **1**–**9**, which were synthesized by reacting different acetophenones with 3,4-dimethoxybenzaldehyde following known literature methods [37,38,39], were reacted with chlorosulfonic acid at room temperature to generate the chalcone sulfonyl chlorides **10**–**18**. Melting points and spectral data (IR, ^1^H-NMR, ^13^C-NMR and nanoESI-MS) were consistent with literature data (see Supplementary Materials for ^1^H-NMR, ^13^C-NMR, HSMS of representative compounds). The sulfonyl chlorides **10**–**18** were deemed pure enough without recrystallization and reacted with bromine in glacial acetic acid at room temperature, which yielded the chalcone dibromo sulfonyl chlorides **19**–**27**. Subsequently, the dibromo chalcone sulfonyl chlorides **19**–**27** were treated with ammonia and four primary amines (methylamine, ethylamine, aniline and benzylamine) to afford a benzothiazine library of 45 compounds **28**–**72**. The structures of the newly synthesized benzothiazines **28**–**72** were appropriately characterized by spectral data. The IR spectra of compounds **28**–**72** confirmed the presence of the carbonyl signal in the region 1650–1720 cm^−1^ and all these compounds exhibited additional bands corresponding to the SO_2_ group (ν_max_ 1340 and 1170 cm^−1^).

**Figure 2:**
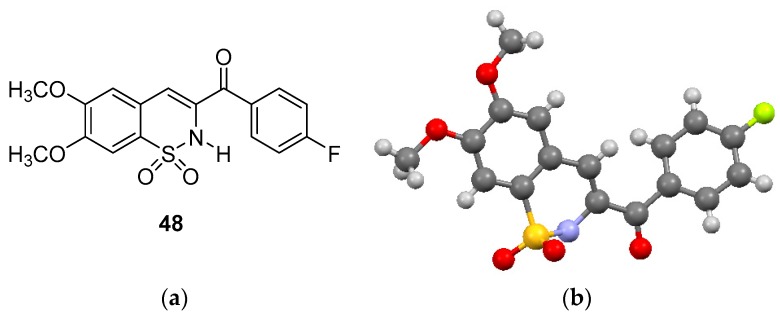
(**a**) Structure of (6,7-dimethoxy-1,1-dioxido-2*H*-benzo[*e*][1,2]thiazin-3-yl)(4-fluoro-phenyl)methanone and (**b**) ORTEP diagram of **48**.

Compounds **29**, **34**, **39**, **54**, **59**, **64** and **69** formed when the dibromo chalcone sulfonyl chlorides were reacted with methylamine. The ^1^H-NMR of these compounds showed the presence of the methylamino group in the δ 2.2–2.8 region. The methine protons all appeared as doublet-of-doublets in all the 1,2-benzothizaines, as expected, with the exception of compound **48** which did not show peaks for the two methine protons. Compound **48** showed two singlets at δ 3.93 and δ 4.01 for the two methoxy groups and three singlets in the aromatic region between δ 6.86–7.84. A broad singlet at δ 8.20 was observed for the amino proton which was exchangeable with D_2_O. The definitive structure of compound **48** was confirmed by spectral and X-ray analysis with the ORTEP representation of the molecular structure shown in Figure 2b.

### 2.2. Antimicrobial Screening

The synthesized compounds **28**–**72** (Table 1) dissolved in DMSO were evaluated for their antimicrobial activity against bacterial strains of *Bacillus subtilis* ATCC 6633, *Staphylococcous aureus* ATCC 6538, *Salmonella typhimurium* ATCC 14028 (*Salmonella enterica)* and *Proteus vulgaris* ATCC 13315 by determining their minimum inhibitory concentrations (MICs) using a micro-broth dilution technique and their minimum bactericidal concentrations (MBCs). Their activities were compared with those of the known antibacterial streptomycin. There was no inhibition of growth of any of the bacteria in the presence of DMSO up to 5% concentration; however, there was a slight decrease in absorbance between concentrations of 5% to 8%, but after 8% there was a steep decrease in absorbance showing inhibition of growth.

The MICs and MBCs of selected compounds are presented against both Gram-positive and Gram-negative bacterial strains in Table 2. Results of the anti-bacterial activity showed that the MICs and MBCs of compounds (**31**, **33**, **38**, **43**, **45**, **50**, **55**, **58**, **60**, **63** and **68**) varied between the ranges of 25–600 µg/mL for *B. subtilis*. The MICs range of these compounds for *S. aureus* varied between 100–500 µg/mL whereas only compounds **53**, **58** and **60** showed a bactericidal effect in the range of 200–400 µg/mL. None of the compounds showed any activity against the selected Gram-negative bacteria as MIC values were higher than 600 µg/mL.

The MIC of most of the compounds that showed antimicrobial activity against bacterial species *B. subtilis* and *S. aureus* was quite high (400–600 μg/mL) as compared to streptomycin which has a MIC of 12.5 μg/mL for Gram-positive bacteria (Table 2), therefore showing very weak antibacterial activity as compared to streptomycin. Only compounds **33**, **38**, **43** and **58** showed MIC values between 25–50 μg/mL for *B. subtilis* (see Supplementary Materials Figures S1–S6).

## 3. Discussion

Prompted by the well-established antibacterial properties of benzothiazines, a series of 1,2-benzothiazine derivatives, covering different modifications of the benzothiazine scaffold, were synthesized from readily available chalcones. The title compounds were assayed in vitro for the evaluation of their antimicrobial activity against Gram-positive and Gram-negative bacteria.

The compounds with a hydrogen or an ethyl group on the nitrogen in the thiazine rings showed antibacterial properties against Gram-positive bacteria. The structure–antimicrobial activity relationship in the synthesized compounds revealed that **33**, **38**, **43**, **53**, **58**, **63** and **68** inhibited the growth of Gram-positive bacteria. These compounds all contain a hydrogen atom on the nitrogen of the thiazine ring. This suggests that the amino group in the thiazine ring may be playing a role in the antibacterial property of these compounds. The results, therefore, support previous work which revealed that 4*H*-1,4-benzothiazines showed antibacterial activity against Gram-positive bacteria and fungi but no significant activity against Gram-negative bacteria [37]. In addition to this, compounds with an ethyl moiety on the nitrogen, **45**, **50**, **55** and **60**, showed some inhibition of Gram-positive bacteria, though at higher concentrations.

In previous work on 1,4-benzothiazines, it was noted that the presence of an extra nitrogen and oxygen atom increases the lipophilicity of the compounds [37]. They reported that in a series of 1,2-benzothiazine-1,1-dioxides (N-methyl analogues), the compounds with greater lipophilicity possessed higher antibacterial activity. In the current work, compounds **29**, **34**, **39**, **54**, **59**, **64** and **69** (N-methyl analogues) showed no activity, while **33**, **38**, **43**, **53**, **58**, **63** and **68** showed weak antibacterial activity. These results are in contrast with the work of Ahmed and colleagues [37]. On the other hand, compounds **45**, **50**, **55** and **60** (N-ethyl analogues) showed some antibacterial activity which is in line with previous work [37].

The reaction of methylamine with the dibromo sulfonyl chlorides **19**–**27** unexpectedly resulted in the 4-methyamino derivatives **29**, **34**, **39**, **54**, **59**, **64** and **69** which showed no antibacterial activity. However, the compounds with a bromine atom on carbon-4 in the thiazine ring displayed weak antibacterial activity, which would suggest that a bromine atom may be playing some role. This is supported by the fact that compound **48** did not show any antibacterial activity.

The presence of different substituents in the benzene ring of the benzoyl moiety also had an effect on the antibacterial activity against *B. subtilis*. Compounds with a chlorine or bromine atom or a methyl group in the *para* position showed increased antibacterial activity and a bromine atom in the *meta* position gave similar results. Amongst the compounds which contain no substituents in the benzene ring, only **31** showed very weak antibacterial activity (MIC of 500 μg/mL for *B. subtilis* and *S. aureus*) while compounds **28**–**30** and **32** showed no activity. However, compounds substituted with methyl, chloro, bromo and fluoro substituents in the *meta* or *para* position showed inhibition for *B. subtilis*. The *ortho*-substituted compounds with the same substituents inhibited *S. aureus* weakly.

In summary, the study conducted in this work has designed a simple synthetic route to 1,2-benzothiazines from readily available starting materials. Some of the compounds (**33**, **38**, **43**, **45**, **50**, **53**, **55** and **58**) synthesized, based on a library of 45 structures, showed some biological activity against Gram-positive bacteria *B. subtilis* and *S. aureus*, though the antibacterial activity was very weak as compared to the reference streptomycin standard. Based on our results, in vitro cytotoxicity work was not conducted for these compounds.

## 4. Experimental Section

### 4.1. General Information

All chemicals were purchased form Sigma Aldrich (St. Louis, MO, USA) and were used without any further purification. Melting points were determined using a Gallenkamp melting point apparatus (Thermo Fisher Scientific, Paisley, UK) and are uncorrected. The NMR spectra were recorded using a 600 MHz spectrometer (JEOL Co Ltd., Tokyo, Japan) with tetramethylsilane as internal standard and solvents as indicated. Chemical shifts were measured in ppm (δ) relative to TMS (0.00 ppm). Coupling constants (*Ј*) are reported in Hertz (Hz). The following abbreviations are used to describe the signal multiplicities: s (singlet), d (doublet), t (triplet), q (quartet) and m (multiplet). Accurate mass measurements were made at the National Mass Spectrometry Facility using Thermo Scientific LTQ Orbitrap XL with Advion Nanomate nanoelectrospray system (Waltham, MA, USA). Infrared spectra were recorded with a Varian spectrophotometer as KBr discs. TLC (thin layer chromatography) was performed using silica gel plates by dissolving the compound in dichloromethane and using the solvent system dichloromethane and diethyl ether. Clog P values were calculated using ChemBioDraw Ultra 13.0 (CambridgeSoft, Perkin Elmer, UK).

### 4.2. Synthesis of Chalcones

Chalcones **1**–**9** were synthesized by the well-established procedure using acetophenones and 3,4-dimethoxybenzaldehyde [38].

### 4.3. General Procedure for the Synthesis of Chalcone Sulfonyl Chlorides ***10**–**18***

The chalcones **1**–**9**, (10 g; 0.032 mol) were added in portions to stirred chlorosulfonic acid (37.67 g; 0.32 mol) in an ice bath. After the addition was complete the reaction mixture was left stirring at room temperature. Progress of the reaction was monitored by thin layer chromatography (TLC). When the reaction was complete (24 h), the mixture was poured slowly over ice to remove the excess chlorosulfonic acid. The sulfonyl chloride was filtered by suction filtration and washed with cold water acetonitrile mixture. The resulting precipitate was considered pure enough to be used in subsequent reactions by TLC analysis.

### 4.4. General Procedure for the Synthesis of Dibromo Chalcone Sulfonyl Chlorides ***19**–**27***

The crude chalcone sulfonyl chloride **10**–**18** (10 g; 0.032 mol) was added to glacial acetic acid (125 mL) with stirring. The resulting mixture was stirred at room temperature and to the stirred mixture was added bromine (20.48 g; 0.13 mol) dissolved in 50 mL glacial acetic acid. The mixture was stirred until a precipitate was formed which was filtered and washed with cold glacial acetic acid.

### 4.5. General Procedure for the Synthesis of Benzothiazines ***28**–**72***

To a stirred solution, at room temperature compounds **19**–**27**, (1 g) in ethanol (25 mL) were added drop-wise to a primary amine (3 mol equivalent). The mixture was warmed on a water bath for 10–20 min making sure that the solvent does not evaporate. The mixture was then stirred at room temperature for a further half hour and then ice was added until a precipitate appeared. The resulting solid was filtered and allowed to dry overnight and recrystallized from the appropriate solvent.

*(4-Bromo-6,7-dimethoxy-1,1-dioxido-3,4-dihydro-2H-benzo[e][1,2]thiazin-3-yl)(phenyl)methanone* (**28**). Recrystallization solvent: ethanol; yield 68.7%; mp 203–204 °C; IR (KBr, ν cm^−1^) 1688.9 (C=O), 1598.8 (C-C, aromatic), 1329.6, 1146.2 (SO_2_). ^1^H-NMR (CDCl_3_): δ 3.87 (d, 1H), 3.93 (s, 3H, OCH_3_), 3.95 (s, 3H, OCH_3_), 4.51(d, 1H), 7.00–8.10 (m, 7H, Ar-H). ^13^C-NMR (CDCl_3_): δ 30.00, 46.00, 54.25, 56.51, 103.00, 107.00, 112.50, 117.00, 120.00, 128.50, 134.00, 138.00, 149.00, 151.50, 192.10. HR-MS (nES) *m*/*z* calcd [M + H]^+^ 346.0744: observed 346.0741.

*(6,7-Dimethoxy-2-methyl-4-(methylamino)-1,1-dioxido-3,4-dihydro-2H-benzo[e][1,2]thiazin-3-yl)(phenyl)methanone* (**29**). Recrystallization solvent: ethanol; yield 79.5%; mp 190–192 °C. IR (KBr, ν cm^−1^) 1598.1 (C=O), 1545.0 (C-C, aromatic), 1383.6, 1157.1 (SO_2_). ^1^H-NMR (CDCl_3_): δ 2.61 (d, 3H, CH_3_), 2.80 (s, 3H, CH_3_), 3.93 (s, 3H, OCH_3_), 3.97 (s, 3H, OCH_3_), 5.63(d, 1H), 6.80 (s, 1H), 7.24–7.79 (m, 7H, Ar-H), 11.4 (broad singlet, 1H, NH (exchangeable with D_2_O). ^13^C-NMR (CDCl3): δ 9.00, 11.00, 30.10, 30.40, 32.10, 32.20, 55.10, 54.20, 101.00, 112.00, 113.00, 126.00, 126.40, 126.81, 128.37, 128.40, 132.00, 160.00, 192.00. HR-MS (nES) *m*/*z* calcd [M + H]^+^ 391.1322: observed 391.1322.

*(4-Bromo-2-ethyl-6,7-dimethoxy-1,1-dioxido-3,4-dihydro-2H-benzo[e][1,2]thiazin-3-yl)(phenyl)methanone* (**30**). Recrystallization solvent: ethanol; yield 93.0%; mp 200–201 °C. IR (KBr, ν cm^−1^) 1693.5(C=O), 1594.7 (C-C, aromatic), 1365.8, 1136.0 (SO_2_). ^1^H-NMR (CDCl_3_): δ 1.32 (t, 3H, CH_3_), 3.48 (q, 2H, CH_2_), 3.68 (s, 3H, OCH_3_), 3.88 (s, 3H, OCH_3_), 5.12 (d, 1H, CH), 5.39 (d, 1H, CH), 6.68–7.92 (7H, Ar-H). ^13^C-NMR (CDCl_3_): 13.20, 4594, 48.83, 56.20, 56.32, 62.85, 102.61, 108.00, 128.82, 128.90, 129.10, 130.00, 134.10, 134.57, 150.75, 152.83, 193.70. HR-MS (nES) *m*/*z* calcd [M + H]^+^ 454.0318: observed 454.0316.

*(4-Bromo-6,7-dimethoxy-1,1-dioxido-2-phenyl-3,4-dihydro-2H-benzo[e][1,2]thiazin-3-yl)(phenyl)methanone* (**31**). Recrystallization solvent: ethanol; yield 51.5%; mp 121–124 °C. IR (KBr, ν cm^−1^) 1689.1 (C=O), 1597.3 (C-C, aromatic), 1382.9, 1142.8 (SO_2_). ^1^H-NMR (CDCl_3_): δ 3.77 (s, 3H, OCH_3_), 4.02 (s, 3H, OCH_3_), 5.759(d, 1H, CH), 7.04 (d, 1H, CH), 7.10–8.12 (m, 12H, Ar-H). ^13^C-NMR (CDCl_3_): δ 29.07, 43.56, 56.46, 56.90, 11.25, 112.45, 121.40, 122.28, 125.30, 129.00, 129.03, 129.24, 130.10, 130.70, 134.20, 136.10, 148.20, 152.25, 190.30. HR-MS (nES) *m*/*z* calcd [M + H]^+^ 502.0318: observed 502.0309.

*(2-Benzyl-4-bromo-6,7-dimethoxy-1,1-dioxido-3,4-dihydro-2H-benzo[e][1,2]thiazin-3-yl)(phenyl)methanone* (**32**). Recrystallization solvent: ethanol; yield 91.8%; mp 133–134 °C. IR (KBr, ν cm^−1^) 1685.7 (C=O), 1589.7 (C-C, aromatic), 1359.9, 1145.8 (SO_2_). ^1^H-NMR (CDCl_3_): 3.90 (s, 3H, OCH_3_), 3.96 (s, 3H, OCH_3_), 4.38 (d, 1H, CH_2_), 4.63 (d, 1H, CH_2_), 5.00 (d, 1H, CH), 5.37 (d, 1H, CH), 7.11–7.85 (12H, Ar-H). ^13^C-NMR (CDCl_3_): δ 29.69, 48.50, 52.00, 56.38, 60.50, 98.50, 101.50, 109.00, 122.00, 123.50, 124.00, 128.12, 128.13, 128.68, 128.90. 129.00, 134.00, 135.00, 136.5, 194.00.

*(4-Bromo-6,7-dimethoxy-1,1-dioxido-3,4-dihydro-2H-benzo[e][1,2]thiazin-3-yl)(p-tolyl)methanone* (**33**). Recrystallization solvent: ethanol; yield 50.6%; mp 176–177 °C. IR (KBr, ν cm^−1^) 1681.6 (C=O), 1505.9 (C-C, aromatic), 1329.3, 1153.8 (SO_2_). ^1^H-NMR (CDCl_3_): δ 2.41 (s, 3H, CH_3_), 3.85 (d, 1H, CH), 3.92 (s, 3H, OCH_3_), 3.95 (s, 3H, OCH_3_), 4.50 (d, 1H, CH), 7.00–8.99 (m, 6H, Ar-H). ^13^C-NMR (CDCl_3_): δ 21.84, 29.68, 45.94, 55.50, 56.48, 104.18, 106.64, 125.00, 129.05, 129.21, 129.73, 146.00, 151.00, 154.00, 189.60.

*(6,7-Dimethoxy-2-methyl-4-(methylamino)-1,1-dioxido-3,4-dihydro-2H-benzo[e][1,2]thiazin-3-yl)(p-tolyl)methanone* (**34**). Recrystallization solvent: ethanol; yield 79.7%; mp 183–184 °C. IR (KBr, ν cm^−1^) 1698.9 (C=O), 1575.0 (C-C, aromatic), 1383.0, 1161.1 (SO_2_). ^1^H-NMR (CDCl_3_): δ 2.34 (s, 3H, CH_3_), 2.59 (s, 3H, CH_3_), 2.80 (d, 3H, CH_3_), 3.93 (s, 3H, OCH_3_), 3.96 (s, 3H, OCH_3_), 5.62 (d, 1H, CH), 6.81 (d, 1H, CH), 7.16–7.70 (m, 6H, Ar-H), 11.30, (broad singlet, 1H, NH (exchangeable with D_2_O). ^13^C-NMR (CDCl_3_): δ 21.41, 29.16, 30.89, 31.66, 56.46, 56.47, 111.86, 112.71, 126.86, 129.00, 129.05, 137.50, 142.00, 148.10, 152.00, 165.00, 188.10. HR-MS (nES) *m*/*z* calcd [M + H]^+^ 405.1479: observed 405.1474.

*(4-Bromo-2-ethyl-6,7-dimethoxy-1,1-dioxido-3,4-dihydro-2H-benzo[e][1,2]thiazin-3-yl)(p-tolyl)methanone* (**35**). Recrystallization solvent: ethanol; yield 86.0%; mp 157–158 °C. IR (KBr, ν cm^−1^) 1670.8 (C=O), 1605.5 (C-C, aromatic), 1303.6, 1150.4 (SO_2_). ^1^H-NMR (CDCl_3_): δ 1.03 (t, 3H, CH_3_), 2.41 (s, 3H, CH_3_), 3.34 (q, 2H, CH_2_), 3.94 (s, 3H, OCH_3_), 3.96 (s, 3H, OCH_3_), 5.05 (d, 1H, CH), 5.37 (d, 1H, CH), 7.17–7.91 (6H, Ar-H). ^13^C-NMR (CDCl_3_): δ 12.77, 21.77, 29.68, 45.30, 47.56, 56.39, 61.73, 102.27, 109.15, 129.19, 129.20, 129.74, 130.05, 132.00, 146.00, 151.80, 153.00, 192.80. HR-MS (nES) *m*/*z* calcd [M + H]^+^ 468.0475: observed 468.0472.

*(4-Bromo-6,7-dimethoxy-1,1-dioxido-2-phenyl-3,4-dihydro-2H-benzo[e][1,2]thiazin-3-yl)(p-tolyl)methanone* (**36**). Recrystallization solvent: ethanol; yield 93.6%; mp 146–147 °C. IR (KBr, ν cm^−1^) 1670.1 (C=O), 1598.9 (C-C, aromatic), 1382.6, 1138.5 (SO_2_). ^1^H-NMR (CDCl_3_): δ 2.4 (s, 3H, CH_3_), 3.71 (d, 1H, CH), 3.75 (s, 3H, OCH_3_), 4.00 (s, 3H, OCH_3_), 5.75 (d, 1H, CH), 7.09–8.01 (m, 11H, Ar-H). ^13^C-NMR (CDCl_3_): δ 29.68, 44.00, 46.00, 56.49, 56.50, 112.00, 114.00, 122.13, 125.45, 129.14, 129.20, 129.21, 129.73, 134.05, 145.40, 148.05, 152.34, 190.10. HR-MS (nES) *m*/*z* calcd [M + H]^+^ 516.0475: observed 516.0469.

*(2-Benzyl-4-bromo-6,7-dimethoxy-1,1-dioxido-3,4-dihydro-2H-benzo[e][1,2]thiazin-3-yl)(p-tolyl)methanone* (**37**). Recrystallization solvent: ethanol; yield 94.8%; mp 151–152 °C. IR (KBr, ν cm^−1^) 1680.3 (C=O), 1606.9 (C-C, aromatic), 1315.7, 1149.1 (SO_2_). ^1^H-NMR (CDCl_3_): δ 2.36 (s, 3H, CH_3_), 3.89 (s, 3H, OCH_3_), 3.96 (s, 3H, OCH_3_), 4.36 (d, 1H, CH_2_), 4.62 (d, 1H, CH_2_), 4.98 (d, 1H), 5.35 (d, 1H), 7.12–7.75 (m, 11H, Ar-H). ^13^C-NMR (CDCl_3_): δ 21.77, 29.69, 48.07, 51.18, 56.32, 56.37, 102.24, 108.91, 127.85, 128.67, 128.92, 128.96, 129.12, 129.20, 130.10, 132.00, 135.07, 146.05, 151.10, 153.20, 192.45. HR-MS (nES) *m*/*z* calcd [M + H]^+^ 530.0631: observed 530.0626.

*(4-Bromo-6,7-dimethoxy-1,1-dioxido-3,4-dihydro-2H-benzo[e][1,2]thiazin-3-yl)(4-chlorophenyl)methanone* (**38**). Recrystallization solvent: ethanol; yield 73.1%; mp 184–185 °C. IR (KBr, ν cm^−1^) 1686.0 (C=O), 1589.0 (C-C, aromatic), 1334.4, 1152.3 (SO_2_). ^1^H-NMR (CDCl_3_): δ 3.80 (s, 1H, CH), 3.93 (s, 3H, OCH_3_), 3.95 (s, 3H, OCH_3_), 4.50 (d, 1H, CH), 7.00–8.06 (m, 6 H, Ar-H). ^13^C-NMR (CDCl_3_): δ 29.70, 45.85, 55.43, 56.51, 104.19, 106.64, 124.00, 128.00, 129.46, 130.49, 133.00, 141.00, 151.00, 154.00, 182.00. HR-MS (nES) *m*/*z* calcd [M − Br]^+^ 380.0354: observed 380.0356.

*(4-Chlorophenyl)(6,7-dimethoxy-2-methyl-4-(methylamino)-1,1-dioxido-3,4-dihydro-2H-benzo[e][1,2]thiazin-3-yl)methanone* (**39**). Recrystallization solvent: ethanol; yield 83.3%; mp 252–253 °C. IR (KBr, ν cm^−1^) 1668.1 (C=O), 1541.2 (C-C, aromatic), 1381.2, 1144.7 (SO_2_). ^1^H-NMR (CDCl_3_): δ 2.60 (s, 3H, CH_3_), 2.81 (d, 3H, NHCH_3_), 3.93 (s, 3H, OCH_3_), 3.96 (s, 3H, OCH_3_), 4.85 (broad singlet, 1H, NH (exchangeable with D_2_O), 5.57 (d, 1H, CH), 6.78 (d, 1H, CH), 7.24–7.71 (m, 6H, Ar-H). ^13^C-NMR (CDCl_3_): δ 29.60, 29.69, 32.00, 56.47, 56.49, 91.00, 111.06, 112.25, 127.00, 128.21, 128.57, 137.35, 138.56, 149.34, 152.00, 163.00, 186.25. HR-MS (nES) *m*/*z* calcd [M + H]^+^ 425.0932: observed 425.0931.

*(4-Bromo-2-ethyl-6,7-dimethoxy-1,1-dioxido-3,4-dihydro-2H-benzo[e][1,2]thiazin-3-yl)(4-chlorophenyl)methanone* (**40**). Recrystallization solvent: ethanol; yield 64.3%; mp 185–186 °C. IR (KBr, ν cm^−1^) 1681.8 (C=O), 1589.9 (C-C, aromatic), 1364.5, 1136.0 (SO_2_). ^1^H-NMR (CDCl_3_): δ 1.26 (t, 3H, CH_3_), 3.58 (q, 2H, CH_2_), 3.58 (d, 1H, CH), 3.82 (s, 3H, OCH_3_), 3.91 (s, 3H, OCH_3_), 5.13 (d, 1H, CH), 6.78–7.90 (m, 6H, Ar-H). ^13^C-NMR (CDCl_3_): δ 13.48, 29.69, 39.54, 44.29, 56.28, 56.49, 102.47, 105.91, 127.00, 129.24, 129.67, 131.85, 134.25, 140.05, 150.00, 153.75, 197.00.

*(4-Bromo-6,7-dimethoxy-1,1-dioxido-2-phenyl-3,4-dihydro-2H-benzo[e][1,2]thiazin-3-yl)(4-chlorophenyl)methanone* (**41**). Recrystallization solvent: ethanol; Yield 76.8%; mp 162–164 °C. IR (KBr, ν cm^−1^) 1689.4 (C=O), 1590.1 (C-C, aromatic), 1343.2, 1146.4 (SO_2_). ^1^H-NMR (CDCl_3_): δ 3.76 (s, 3H, OCH_3_), 4.01 (s, 3H, OCH_3_), 5.70 (d, 1H, CH), 7.10 (d, 1H, CH), 7.24–8.06 (m, 11H, Ar-H). ^13^C-NMR (CDCl_3_): δ 29.70, 43.70, 46.25, 56.02, 56.20, 112.20, 113.00, 122.00, 126.05, 129.20, 129.27, 129.42, 130.41, 136.00, 141.25, 149.10, 153.70, 189.50. HR-MS (nES) *m*/*z* calcd [M + H]^+^ 535.9929: observed 535.9926.

*(2-Benzyl-4-bromo-6,7-dimethoxy-1,1-dioxido-3,4-dihydro-2H-benzo[e][1,2]thiazin-3-yl)(4-chlorophenyl)methanone* (**42**). Recrystallization solvent: ethanol; yield 58.1%; mp 166–168 °C. IR (KBr, ν cm^−1^) 1682.5 (C=O), 1589.0 (C-C, aromatic), 1357.9, 1146.9 (SO_2_). ^1^H-NMR (CDCl_3_): δ 3.92 (s, 3H, OCH_3_), 3.95 (s, 3H, OCH_3_), 4.35 (d, H, CH_2_), 4.57 (d, 1H, CH_2_), 4.92 (d, 1H), 5.48 (d, 1H), 7.10–7.77 (m, 11H, Ar-H). ^13^C-NMR (CDCl_3_): 47.71, 51.71, 56.32, 56.37, 60.90, 102.28, 108.85, 128.12, 128.66, 128.90, 129.67, 129.40, 130.33, 131.25, 132.80, 135.20, 141.20, 150.72, 152.29. 191.73. HR-MS (nES) *m*/*z* calcd [M + H]^+^ 550.0085: observed 550.0082.

*(4-Bromo-6,7-dimethoxy-1,1-dioxido-3,4-dihydro-2H-benzo[e][1,2]thiazin-3-yl)(4-bromophenyl)methanone* (**43**). Recrystallization solvent: ethanol; yield 69.8%; mp 193–194 °C. IR (KBr, ν cm^−1^) 1686.0 (C=O), 1585.1 (C-C, aromatic), 1333.8, 1152.1 (SO_2_). ^1^H-NMR (CDCl_3_): δ 3.79 (d, 1H, CH), 3.93 (s, 3H, OCH_3_), 3.95 (s, 3H, OCH_3_), 4.50 (d, 1H, CH), 7.00–7.97 (m, 6 H, Ar-H). ^13^C-NMR (CDCl_3_): δ 29.70, 45.00, 54.50, 56.51, 106.00, 106.05, 124.00, 127.00, 130.52, 130.60, 132.46, 133.00, 133.05, 154.00, 195.00. HR-MS (nES) *m*/*z* calcd [M − Br]^+^ 423.9849: observed 423.9850.

*(4-Bromo-6,7-dimethoxy-2-methyl-1,1-dioxido-3,4-dihydro-2H-benzo[e][1,2]thiazin-3-yl)(4-bromophenyl)methanone* (**44**). Recrystallization solvent: ethanol; yield 78.8%; mp 189–190 °C. IR (KBr, ν cm^−1^) 1677.3 (C=O), 1585.3 (C-C, aromatic), 1302.6, 1150.3 (SO_2_). ^1^H-NMR (CDCl_3_): δ 2.85 (s, 3H, CH_3_), 3.94 (s, 3H, OCH_3_), 3.96 (s, 3H, OCH_3_), 4.88 (d, 1H, CH), 5.31 (d, 1H, CH), 7.24–7.86 (m, 6 H, Ar-H). ^13^C-NMR (CDCl_3_): δ 29.69, 37.38, 46.81, 56.45, 56.56, 102.66, 109.42128.50, 129.00, 129.50, 130.47, 132.29, 133.10, 151.70, 152.00, 192.00.

*(4-Bromo-2-ethyl-6,7-dimethoxy-1,1-dioxido-3,4-dihydro-2H-benzo[e][1,2]thiazin-3-yl)(4-bromophenyl)methanone* (**45**). Recrystallization solvent: ethanol; yield 86.3%; mp 171–172 °C. IR (KBr, ν cm^−1^) 1676.6 (C=O), 1583.7 (C-C, aromatic), 1306.2, 1149.4 (SO_2_). ^1^H-NMR (CDCl_3_): δ 1.02 (t, 3H, CH_3_), 3.33 (q, 2H, CH_2_), 3.95 (s, 3H, OCH_3_), 3.96 (s, 3H, OCH_3_), 5.02 (d, 1H, CH), 5.32 (d, 1H, CH), 7.11–7.87 (m, 6H, Ar-H). ^13^C-NMR (CDCl_3_): δ 29.69, 45.20, 47.75, 56.90, 57.00, 62.00, 102.10, 108.80, 110.50, 112.30, 130.20, 130.45, 132.40, 132.60, 151.80, 151.80, 152.10, 192.00. HR-MS (nES) *m*/*z* calcd [M + H]^+^ 517.9267: observed 517.9264.

*(4-Bromo-6,7-dimethoxy-1,1-dioxido-2-phenyl-3,4-dihydro-2H-benzo[e][1,2]thiazin-3-yl)(4-bromophenyl)methanone* (**46**). Recrystallization solvent: ethanol; yield 89.5%; mp 218–219 °C. IR (KBr, ν cm^−1^) 1688.7 (C=O), 1585.0 (C-C, aromatic), 1382.3, 1145.6 (SO_2_). ^1^H-NMR (CDCl_3_): δ 3.76 (s, 3H, OCH_3_), 4.01 (s, 3H, OCH_3_), 5.70 (d, 1H, CH), 7.05 (d, 1H, CH), 7.11–7.98 (m, 11H, Ar-H). ^13^C-NMR (CDCl_3_): δ 29.70, 43.45, 56.05, 56.40, 11.30, 112.10, 120.30, 120.45, 125.37, 127.35, 128.10, 128.45, 129.30, 130.10, 132.10, 132.89, 148.37, 152.35, 189.75. HR-MS (nES) *m*/*z* calcd [M + H]^+^ 579.9423: observed 579.9416.

*(2-Benzyl-4-bromo-6,7-dimethoxy-1,1-dioxido-3,4-dihydro-2H-benzo[e][1,2]thiazin-3-yl)(4-bromophenyl)methanone* (**47**). Recrystallization solvent: ethanol; yield 80.6%; mp 176–177 °C. IR (KBr, ν cm^−1^) 1685.5 (C=O), 1585.3 (C-C, aromatic), 1293.6, 1146.6 (SO_2_). ^1^H-NMR (CDCl_3_): δ 3.89 (s, 3H, OCH_3_), 3.96 (s, 3H, OCH_3_), 4.33 (d, 1H, CH_2_), 4.58 (d, 1H, CH_2_), 4.91 (d, 1H, CH), 5.28 (d, 1H, CH), 7.09–7.70 (m, 11H, Ar-H). ^13^C-NMR (CDCl_3_): δ 29.95, 47.75, 51.45, 56.00, 60.24, 102.00, 108.50, 128.20, 128.69, 128.94, 128.98, 130.39, 132.31, 132.70, 135.10, 135.20, 136.50, 150.50, 152.34, 192.00. HR-MS (nES) *m*/*z* calcd [M + H]^+^ 593.9580: observed 593.9579.

*(6,7-Dimethoxy-1,1-dioxido-2H-benzo[e][1,2]thiazin-3-yl)(4-fluorophenyl)methanone* (**48**). Recrystallization solvent: ethanol; yield 58.0%; mp 249–250 °C. IR (KBr, ν cm^−1^) 1638.2 (C=O), 1597.3 (C-C, aromatic), 1363.4, 1152.7 (SO_2_). ^1^H-NMR (CDCl_3_): δ 3.93 (s, 3H, OCH_3_), 4.01 (s, 3H, OCH_3_), 6.86–7.84 (m, 7H, Ar-H), 8.2 (broad singlet, 1H, NH (exchangeable with D_2_O). HR-MS (nES) *m*/*z* calcd [M + H]^+^ 534.0381: observed 534.0376.

*(4-Bromo-6,7-dimethoxy-2-methyl-1,1-dioxido-3,4-dihydro-2H-benzo[e][1,2]thiazin-3-yl)(4-fluorophenyl)methanone* (**49**). Recrystallization solvent: ethanol; yield 85.7%; mp 195–196 °C. IR (KBr, ν cm^−1^) 1685.3 (C=O), 1598.0 (C-C, aromatic), 1325.1, 1146.5 (SO_2_). ^1^H-NMR (CDCl_3_): δ 2.10 (s, 3H, CH_3_), 3.90 (s, 3H, OCH_3_), 3.91 (s, 3H, OCH_3_), 4.84 (d, 1H, CH), 5.30 (d, 1H, CH), 7.10–8.00 (m, 6H, Ar-H). ^13^C-NMR (CDCl_3_): δ 29.70, 37.25, 46.25, 56.44, 56.50, 103.56, 110.65, 116.15, 117.50, 128.00, 129.67, 131.45, 132.00, 150.50, 152.00, 192.00. HR-MS (nES) *m*/*z* calcd [M + H]^+^ 458.0068: observed 458.0063.

*(4-Bromo-2-ethyl-6,7-dimethoxy-1,1-dioxido-3,4-dihydro-2H-benzo[e][1,2]thiazin-3-yl)(4-fluorophenyl)methanone* (**50**). Recrystallization solvent: ethanol; yield 95.3%; mp 190–191 °C. IR (KBr, ν cm^−1^) 1693.4 (C=O), 1598.3 (C-C, aromatic), 1345.8, 1185.0 (SO_2_). ^1^H-NMR (CDCl_3_): δ 3.92 (s, 3H, OCH_3_), 3.97 (s, 3H, OCH_3_), 5.50 (d, 1H, CH), 6.50 (d, 1H, CH), 6.85–8.10 (m, 6H, Ar-H). ^13^C-NMR (CDCl_3_): δ 16.00, 25.00, 29.70, 38.00, 43.50, 57.00, 110.05, 111.00, 117.00, 117.03, 117.05, 124.00, 131.76, 131.790, 132.00, 164.00, 192.00. HR-MS (nES) *m*/*z* calcd [M + H]^+^ 472.0224: observed 472.0212.

*(4-Bromo-6,7-dimethoxy-1,1-dioxido-2-phenyl-3,4-dihydro-2H-benzo[e][1,2]thiazin-3-yl)(4-fluorophenyl)methanone* (**51**). Recrystallization solvent: ethanol; yield 85.2%; mp 170–171 °C. IR (KBr, ν cm^−1^) 1688.9 (C=O), 1598.8 (C-C, aromatic), 1382.7, 1146.8 (SO_2_). ^1^H-NMR (CDCl_3_): δ 3.76 (s, 3H, OCH_3_), 3.94 (s, 3H, OCH_3_), 5.71 (d, 1H, CH), 7.12 (d, 1H, CH), 7.24–8.16 (m, 11H, Ar-H). ^13^C-NMR (CDCl3): δ 29.45, 43.75, 56.34, 56.78, 11.10, 112.25, 116.10, 122.00, 122.30, 125.95, 129.30, 129.46, 130.00, 130.80, 131.30, 131.57, 132.00, 152.40, 189.75. HR-MS (nES) *m*/*z* calcd [M + H]^+^ 520.0224: observed 520.0218.

*(2-Benzyl-4-bromo-6,7-dimethoxy-1,1-dioxido-3,4-dihydro-2H-benzo[e][1,2]thiazin-3-yl)(4-fluorophenyl)methanone* (**52**). Recrystallization solvent: ethanol; yield 87.7%; mp 173–174 °C. IR (KBr, ν cm^−1^) 1683.6 (C=O), 1596.9 (C-C, aromatic), 1356.5, 1147.3 (SO_2_). ^1^H-NMR (CDCl_3_): δ 3.90 (s, 3H, OCH_3_), 3.96 (s, 3H, OCH_3_), 4.38 (d, 1H, CH_2_), 4.60 (d, 1H, CH_2_), 4.98 (d, 1H, CH), 5.31 (d, 1H, CH), 7.10–7.89 (m, 11H, Ar-H). ^13^C-NMR (CDCl_3_): δ 29.95, 47.96, 51.87, 56.21, 56.35, 102.35, 109.00, 116.20, 116.25, 128.00, 128.67, 128.94, 130.05, 131.00, 131.50, 132.00, 135.10, 151.58, 152.00, 192.00. HR-MS (nES) *m*/*z* calcd [M + H]^+^ 534.0381: observed 534.0376.

*(4-Bromo-6,7-dimethoxy-1,1-dioxido-3,4-dihydro-2H-benzo[e][1,2]thiazin-3-yl)(3-chlorophenyl)methanone* (**53**). Recrystallization solvent: ethanol; yield 53.5%; mp 181–182 °C. IR (KBr, ν cm^−1^) 1686.0 (C=O), 1594.0 (C-C, aromatic), 1328.8, 1149.9 (SO_2_). ^1^H-NMR (CDCl_3_): δ 3.81 (d, 1H, CH), 3.94 (s, 3H, OCH_3_), 3.96 (s, 3H, OCH_3_), 4.50 (d, 1H, CH), 7.01–8.04 (m, 6H, Ar-H). ^13^C-NMR (CDCl_3_): δ 45.95, 55.22, 56.51, 56.85, 104.19, 106.65, 127.33, 128.33, 128.84, 129.50, 130.41, 134.51, 135.49, 136.40, 151.44, 154.07, 188.26.

*(3-Chlorophenyl)(6,7-dimethoxy-2-methyl-4-(methylamino)-1,1-dioxido-3,4-dihydro-2H-benzo[e][1,2]thiazin-3-yl)methanone* (**54**). Recrystallization solvent: ethanol; yield 77.0%; mp 176–177 °C. IR (KBr, ν cm^−1^) 1600.9 (C=O), 1505.3 (C-C, aromatic), 1381.1, 1136.4 (SO_2_). ^1^H-NMR (CDCl_3_): δ 2.63 (s, 3H,=3), 2.82 (s, 3H, CH_3_), 3.93 (s, 3H, OCH_3_), 3.97 (s, 3H, OCH_3_), 5.11 (d, 1H, CH), 5.55 (s, 1H, CH), 6.77–7.66 (m, 6H, Ar-H), 11.28, (broad singlet, 1H, NH (exchangeable with D_2_O). ^13^C-NMR (CDCl_3_): δ 29.06, 30.25, 31.84, 56.48, 56.48, 90.98, 111.71, 112.81, 124.82, 126.97, 126.97, 129.57, 130.79, 135.25, 142.00, 149.40, 153.45, 166.00, 186.00. HR-MS (nES) *m*/*z* calcd [M + H]^+^ 425.0932: observed 425.0927.

*(4-Bromo-2-ethyl-6,7-dimethoxy-1,1-dioxido-3,4-dihydro-2H-benzo[e][1,2]thiazin-3-yl)(3-chlorophenyl)methanone* (**55**). Recrystallization solvent: ethanol; yield 67.8%; mp 141–142 °C. IR (KBr, ν cm^−1^) 1688.6 (C=O), 1590.7 (C-C, aromatic), 1300.1, 1143.6 (SO_2_). ^1^H-NMR (CDCl_3_): δ 1.04 (t, 3H, CH_3_), 3.35 (q, 2H, CH_2_), 3.94 (s, 3H, OCH_3_), 3.96 (s, 3H, OCH_3_), 5.02 (d, 1H, CH), 5.32 (d, 1H, CH), 7.18–7.98 (m, 6H, Ar-H). ^13^C-NMR (CDCl_3_): δ 12.65, 45.75, 47, 38, 56.43, 56.75, 61.71, 102.37, 109.07, 127.06, 129.03, 129.91, 130.33, 131.50, 132.50, 134.20, 134.70, 150.87, 152.34, 191.82. HR-MS (nES) *m*/*z* calcd [M + H]^+^ 487.9929: observed 487.9923.

*(4-Bromo-6,7-dimethoxy-1,1-dioxido-2-phenyl-3,4-dihydro-2H-benzo[e][1,2]thiazin-3-yl)(3-chlorophenyl)methanone* (**56**). Recrystallization solvent: ethanol; yield 78.9%; mp 229–230 °C. IR (KBr, ν cm^−1^) 1701.3 (C=O), 1595.8 (C-C, aromatic), 1337.2, 1140.8 (SO_2_). ^1^H-NMR (CDCl_3_): δ 3.84 (s, 3H, OCH_3_), 3.96 (s, 3H, OCH_3_), 5.40 (d, 1H, CH), 5.80 (d, 1H, CH), 6.90–7.42 (m, 11H, Ar-H). ^13^C-NMR (CDCl_3_): δ 47.90, 56.37, 56.90, 63.54, 102.97, 107.62, 125.74, 126.78, 128.74, 128.90, 129.78, 130.28, 131.50, 132.00, 134.10, 134.20, 136.50, 138.00, 151.20, 153.12. HR-MS (nES) *m*/*z* calcd [M + H]^+^ 535.9929: observed 535.9925.

*(2-Benzyl-4-bromo-6,7-dimethoxy-1,1-dioxido-3,4-dihydro-2H-benzo[e][1,2]thiazin-3-yl)(3-chlorophenyl)methanone* (**57**). Recrystallization solvent: ethanol; yield 74.4%; mp 154–155 °C. IR (KBr, ν cm^−1^) 1691.2 (C=O), 1591.8 (C-C, aromatic), 1335.9, 1138.2 (SO_2_). ^1^H-NMR (CDCl_3_): δ 3.90 (s, 3H, OCH_3_), 3.96 (s, 3H, OCH_3_), 4.40 (d, 1H, CH_2_), 4.57 (d, 1H, CH_2_), 4.97 (d, 1H, CH), 5.27 (d, 1H, CH), 7.10–7.85 (m, 11H, Ar-H). ^13^C-NMR (CDCl_3_): δ 47.75, 51.78, 56.35, 56.39, 60.95, 102.31, 108.85, 126.50, 127.69, 128.22, 128.70, 128.93, 130.19, 131.50, 134.17, 134.50, 134.70, 135.30, 135.50, 151.50, 152.10, 192.00. HR-MS (nES) *m*/*z* calcd [M + NH_4_]^+^ 567.0351: observed 567.0345.

*(4-Bromo-6,7-dimethoxy-1,1-dioxido-3,4-dihydro-2H-benzo[e][1,2]thiazin-3-yl)(3-bromophenyl)methanone* (**58**). Recrystallization solvent: ethanol; yield 44.5%; mp 177–178 °C. IR (KBr, ν cm^−1^) 1685.9 (C=O), 1587.6 (C-C, aromatic), 1328.8, 1149.3 (SO_2_). ^1^H-NMR (CDCl_3_): δ 3.80 (d, 1H, CH), 3.93 (s, 3H, OCH_3_), 3.95 (s, 3H, OCH_3_), 4.49 (d, 1H, CH), 7.00–8.19 (m, 6H, Ar-H). ^13^C-NMR (CDCl_3_): δ 29.95, 46.10, 55.10, 56.51, 104.10, 106.80, 123.50, 126.40, 127.50, 128.10, 131.35, 131.86, 136.40, 137.24, 151.75, 153.95, 188.10. HR-MS (nES) *m*/*z* calcd [M + HBr]^+^ 423.9849: observed 423.9850.

*(3-Bromophenyl)(6,7-dimethoxy-2-methyl-4-(methylamino)-1,1-dioxido-3,4-dihydro-2H-benzo[e][1,2]thiazin-3-yl)methanone* (**59**). Recrystallization solvent: ethanol; yield 76.4%; mp 178–179 °C. IR (KBr, ν cm^−1^) 1678.8 (C=O), 1570.4 (C-C, aromatic), 1311.2, 1136.4 (SO_2_). ^1^H-NMR (CDCl_3_): δ 2.63 (s, 3H, CH_3_), 2.82 (d, 3H, NHCH_3_), 3.93 (s, 3H, OCH_3_), 3.97 (s, 3H, OCH_3_), 5.55 (d, 1H, CH), 6.77 (d, 1H, CH), 7.21–7.84 (m, 6H, Ar-H), 11.31 (broad singlet, 1H, NH (exchangeable with D_2_O). ^13^C-NMR (CDCl_3_): δ 29.68, 29.80, 31.83, 56.49, 56.90, 91.20, 111.69, 112.20, 123.10, 125.32, 126.23, 128.10, 129.93, 133.74, 141.35, 149.45, 152.00, 159.56, 186.34. HR-MS (nES) *m*/*z* calcd [M + H]^+^ 469.0427: observed 469.0419.

*(4-Bromo-2-ethyl-6,7-dimethoxy-1,1-dioxido-3,4-dihydro-2H-benzo[e][1,2]thiazin-3-yl)(3-bromophenyl)methanone* (**60**). Recrystallization solvent: ethanol; yield 85.2%; mp 130–131 °C. IR (KBr, ν cm^−1^) 1689.3 (C=O), 1590.2 (C-C, aromatic), 1350.4, 1142.9 (SO_2_). ^1^H-NMR (CDCl_3_): δ 1.02 (t, 3H, CH_3_), 3.36 (q, 2H, CH_2_), 3.94 (s, 3H, OCH_3_), 3.96 (s, 3H, OCH_3_), 5.03 (d, 1H, CH), 5.32 (d, 1H, CH), 7.17–8.14 (m, 6H, Ar-H). ^13^C-NMR (CDCl_3_): δ 12.64, 29.69, 45.74, 47.35, 56.43, 61.70, 101.70, 108.00, 122.10, 126.20, 128.20, 130.05, 131.15, 136.05, 137.25, 138.15, 150.25, 152.15, 192.15. HR-MS (nES) *m*/*z* calcd [M + H]^+^ 531.9423: observed 531.9421.

*(4-Bromo-6,7-dimethoxy-1,1-dioxido-2-phenyl-3,4-dihydro-2H-benzo[e][1,2]thiazin-3-yl)(3-bromophenyl)methanone* (**61**). Recrystallization solvent: ethanol; yield 80.2%; mp 232–233 °C. IR (KBr, ν cm^−1^) 1701.6 (C=O), 1595.3 (C-C, aromatic), 1284.2, 1141.5 (SO_2_). ^1^H-NMR (CDCl_3_): δ 3.85 (s, 3H, OCH_3_), 3.91 (s, 3H, OCH_3_), 5.45 (d, 1H, CH), 5.80 (d, 1H, CH), 6.80–7.94 (m, 11H, Ar-H). ^13^C-NMR (CDCl_3_): δ 51.93, 56.24, 56.38, 56.48, 62.06, 102.53, 108.04, 127.33, 127.43, 128.17, 129.18, 129.40, 130.03, 132.67, 133.84, 134.86, 138.10, 150.96, 152.97, 196.11. HR-MS (nES) *m*/*z* calcd [M + NH_4_]^+^ 596.9689: observed 596.9685.

*(2-Benzyl-4-bromo-6,7-dimethoxy-1,1-dioxido-3,4-dihydro-2H-benzo[e][1,2]thiazin-3-yl)(3-bromophenyl)methanone* (**62**). Recrystallization solvent: ethanol; Yield 96.9%; mp 167–168 °C. IR (KBr, ν cm^−1^) 1690.8 (C=O), 1590.8 (C-C, aromatic), 1334.6, 1137.9 (SO_2_). ^1^H-NMR (CDCl_3_): δ 3.90 (s, 3H, OCH_3_), 3.91 (s, 3H, OCH_3_), 4.40 (d, 1H, CH_2_), 4.56 (d, 1H, CH_2_), 4.97 (d, 1H, CH), 5.26 (d, 1H, CH), 7.10–7.96 (m, 11H, Ar-H). ^13^C-NMR (CDCl_3_): δ 29.69, 47.72, 56.35, 56.39, 60.97, 102.31, 108.85, 122.80, 127.40, 127.75, 128.24, 128.60, 128.71, 128.93, 130.41, 131.89, 135.10, 136.20, 137.25, 151.45, 152.10, 192.23. HR-MS (nES) *m*/*z* calcd [M + NH_4_]^+^ 610.9845: observed 610.9842.

*(4-Bromo-6,7-dimethoxy-1,1-dioxido-3,4-dihydro-2H-benzo[e][1,2]thiazin-3-yl)(2-chlorophenyl)methanone* (**63**). Recrystallization solvent: ethanol; yield 72.8%; mp 185–186 °C. IR (KBr, ν cm^−1^) 1696.5 (C=O), 1677.0 (C-C, aromatic), 1337.3, 1153.7 (SO_2_). ^1^H-NMR (CDCl_3_): δ 3.80 (d, 1H, CH), 3.91 (s, 3H, OCH_3_), 3.95 (s, 3H, OCH_3_), 4.41 (d, 1H, CH), 6.97–7.53 (m, 6H, Ar-H). ^13^C-NMR (CDCl_3_): δ 29.70, 47.50, 56.49, 56.51, 104.00, 106.00, 122.00, 125.00, 126.50, 130.79, 133.90, 147.50, 153.50, 160.0, 161.90, 192.00.

*(2-Chlorophenyl)(6,7-dimethoxy-2-methyl-4-(methylamino)-1,1-dioxido-3,4dihydro-2H-benzo[e][1,2]thiazin-3-yl)methanone* (**64**). Recrystallization solvent: ethanol; yield 65.4%; mp 118–119 °C. IR (KBr, ν cm^−1^) 1602.0 (C=O), 1568.8 (C-C, aromatic), 1309.7, 1147.6 (SO_2_). ^1^H-NMR (CDCl_3_): δ 2.56 (s, 3H, CH_3_), 2.81 (s, 3H, CH_3_), 3.92 (s, 3H, OCH_3_), 3.95 (s, 3H, OCH_3_), 4.91 (d, 1H, CH), 5.26 (s, 1H, CH), 7.24–7.52 (m, 6H, Ar-H), 6.76, (broad singlet, 1H, NH. ^13^C-NMR (CDCl_3_): δ 27.80, 32.00, 56.50, 57.50, 95.00, 101.00, 101.90, 111.00, 112.00, 126.00, 127.00, 129.92, 130.00, 131.00, 132.00, 164.00, 190.00. HR-MS (nES) *m*/*z* calcd [M + H]^+^ 425.0932: observed 425.0928.

*(4-Bromo-2-ethyl-6,7-dimethoxy-1,1-dioxido-3,4-dihydro-2H-benzo[e][1,2]thiazin-3-yl)(2-chlorophenyl)methanone* (**65**). Recrystallization solvent: ethanol; yield 48.2%; mp 129–131 °C. IR (KBr, ν cm^−1^) 1701.3 (C=O), 1589.9 (C-C, aromatic), 1383.4, 1141.1 (SO_2_). ^1^H-NMR (CDCl_3_): δ 1.31 (t, 3H, CH_3_), 3.44 (q, 2H, CH_2_), 3.82 (s, 3H, OCH_3_), 3.95 (s, 3H, OCH_3_), 5.29 (d, 1H, CH), 5.44 (d, 1H, CH), 6.86–7.74 (m, 6H, Ar-H). ^13^C-NMR (CDCl_3_): δ 45.04, 51.63, 52.98, 56.01, 61.49, 62.44, 102.36, 10258, 108.08, 108.72, 127.04, 127.09, 127.68, 130.93, 132.95, 150.85, 152.44, 152.92, 193.34. HR-MS (nES) *m*/*z* calcd [M + H]^+^ 487.9927: observed 487.9918.

*(4-Bromo-6,7-dimethoxy-1,1-dioxido-2-phenyl-3,4-dihydro-2H-benzo[e][1,2]thiazin-3-yl)(2-chlorophenyl)methanone* (**66**). Recrystallization solvent: ethanol; yield 84.2%; mp 209–210 °C. IR (KBr, ν cm^−1^) 1710.0 (C=O), 1587.7 (C-C, aromatic), 1304.6, 1144.7 (SO_2_). ^1^H-NMR (CDCl_3_): δ 3.96 (s, 3H, OCH_3_), 3.98 (s, 3H, OCH_3_), 5.52 (d, 1H, CH), 5.80 (d, 1H, CH), 6.80–7.94 (m, 11H, Ar-H). ^13^C-NMR (CDCl_3_): δ 29.70, 52.05, 56.42, 56.46, 102.45, 108.20, 126.36, 126.78, 127.14, 127.56, 127.85, 128.45, 129.56, 130.04, 132.05, 133.56, 137.34, 136.00, 150.78, 153.45, 196.67. HR-MS (nES) *m*/*z* calcd [M + H]^+^ 535.9929 535: observed 535.9924.

*(2-Benzyl-4-bromo-6,7-dimethoxy-1,1-dioxido-3,4-dihydro-2H-benzo[e][1,2]thiazin-3-yl)(2-chlorophenyl)methanone* (**67**). Recrystallization solvent: ethanol; yield 90.8%; mp 156–157 °C. IR (KBr, ν cm^−1^) 1692.5 (C=O), 1592.0 (C-C, aromatic), 1342.2, 1140.2 (SO_2_). ^1^H-NMR (CDCl_3_): δ 3.89 (s, 3H, OCH_3_), 3.95 (s, 3H, OCH_3_), 4.45 (d, 1H, CH_2_), 4.70 (d, 1H, CH_2_), 5.05 (d, 1H, CH), 5.28 (d, 1H, CH), 7.01–7.42 (m, 11H, Ar-H). ^13^C-NMR (CDCl_3_): δ 29.70, 50.75, 52.52, 56.38, 60.45, 102.34, 108.25, 126.50, 127.34, 128.50, 128.78, 129.04, 129.42, 130.87, 131.50, 132.23, 135.60, 135.80, 136.00, 150.70, 152.10, 194.00. HR-MS (nES) *m*/*z* calcd [M + NH_4_]^+^ 567.0351: observed 567.0342.

*(4-Bromo-6,7-dimethoxy-1,1-dioxido-3,4-dihydro-2H-benzo[e][1,2]thiazin-3-yl)(2-bromophenyl)methanone* (**68**). Recrystallization solvent: ethanol; yield 56.6%; mp 183–184 °C. IR (KBr, ν cm^−1^) 1696.7 (C=O), 1676.3 (C-C, aromatic), 1336.3, 1153.2 (SO_2_). ^1^H-NMR (CDCl_3_): δ 3.85 (s, 3H, OCH_3_), 3.91 (s, 3H, OCH_3_), 5.45 (d, 1H, CH), 5.78 (d, 1H, CH) 6.98–7.93 (m, 6H, Ar-H). ^13^C-NMR (CDCl_3_): δ 29.70, 47.96, 56.41, 63.53, 102.97, 107.62, 124.10, 125.74, 127.21, 128.00, 129.79, 130.50, 131.65, 137.26. 138.05, 150.12, 190.45. HR-MS (nES) *m*/*z* calcd [M − Br]^+^ 423.9849: observed 423.9849.

*(2-Bromophenyl)(6,7-dimethoxy-2-methyl-4-(methylamino)-1,1-dioxido-3,4-dihydro-2H-benzo[e][1,2]thiazin-3-yl)methanone* (**69**). Recrystallization solvent: ethanol; yield 75.2%; mp 122–123 °C. IR (KBr, ν cm^−1^) 1737.9 (C=O), 1655.7 (C-C, aromatic), 1383.0, 1147.1 (SO_2_). ^1^H-NMR (CDCl_3_): δ 2.55 (s, 3H, CH_3_), 2.83 (s, 3H, CH_3_), 3.93 (s, 3H, OCH_3_), 3.95 (s, 3H, OCH_3_), 4.80 (d, 1H, CH), 5.16 (s, 1H, CH), 6.77–7.53 (m, 6H, Ar-H), 11.35, (broad singlet, 1H, NH (exchangeable with D_2_O). ^13^C-NMR (CDCl_3_): δ 29.31, 29.78, 31.82, 56.51, 56.58, 94.73, 11.70, 112.76, 118.00, 127.44, 128.79, 129.40, 130.21, 130.75, 133.01, 143.45, 148.12, 151.75, 164.91, 192.30. HR-MS (nES) *m*/*z* calcd [M + H]^+^ 469.0427: observed 469.0418.

*(4-Bromo-2-ethyl-6,7-dimethoxy-1,1-dioxido-3,4-dihydro-2H-benzo[e][1,2]thiazin-3-yl)(2-bromophenyl)methanone* (**70**). Recrystallization solvent: ethanol; yield 69.3%; mp 129–130 °C. IR (KBr, ν cm^−1^) 1689.5 (C=O), 1590.1 (C-C, aromatic), 1350.4, 1142.8 (SO_2_). ^1^H-NMR (CDCl_3_): δ 1.05 (t, 3H, CH_3_), 3.35 (q, 2H, CH_2_), 3.89 (s, 3H, OCH_3_), 3.95 (s, 3H, OCH_3_), 5.03 (d, 1H, CH), 5.29 (d, 1H, CH), 7.19–8.15 (m, 6H, Ar-H). ^13^C-NMR (CDCl_3_): 12.64, 45.73, 47.34, 56.35, 56.41, 61.69, 102.35, 109.05, 123.37, 127.48, 129.89, 130.53, 131.94, 132.20, 136.00, 137.08, 150.85, 152.32, 191.72.

*(4-Bromo-6,7-dimethoxy-1,1-dioxido-2-phenyl-3,4-dihydro-2H-benzo[e][1,2]thiazin-3-yl)(2-bromophenyl)methanone* (**71**). Recrystallization solvent: ethanol; yield 96.8%; mp 210–211 °C. IR (KBr, ν cm^−1^) 1709.5 (C=O), 1588.1 (C-C, aromatic), 1305.1, 1144.7 (SO_2_). ^1^H-NMR (CDCl_3_): δ 3.96 (s, 3H, OCH_3_), 3.98 (s, 3H, OCH_3_), 5.52 (d, 1H, CH), 5.79 (d, 1H, CH), 7.24–7.62 (m, 11H, Ar-H). ^13^C-NMR (CDCl_3_): δ 29.69, 51.96, 56.42, 56.51, 102.45, 108.23, 119.50, 126.60, 126.85, 127.25, 127.45, 128.85, 128.95, 130.06, 132.32, 132.85, 134.23, 138.45, 151.50, 151.80, 196.10. HR-MS (nES) *m*/*z* calcd [M + H]^+^ 579.9423: observed 579.9423.

*(2-Benzyl-4-bromo-6,7-dimethoxy-1,1-dioxido-3,4-dihydro-2H-benzo[e][1,2]thiazin-3-yl)(2-bromophenyl)methanone* (**72**). Recrystallization solvent: ethanol; yield 81.6%; mp 123–125 °C. IR (KBr, ν cm^−1^) 1690.6 (C=O), 1590.4 (C-C, aromatic), 1334.3, 1137.9 (SO_2_). ^1^H-NMR (CDCl_3_): δ 3.89 (s, 3H, OCH_3_), 3.96 (s, 3H, OCH_3_), 4.39 (d, 1H, CH_2_), 4.55 (d, 1H, CH_2_), 4.97 (d, 1H, CH), 5.27 (d, 1H, CH), 7.10–7.96 (m, 11H, Ar-H). ^13^C-NMR (CDCl_3_): 47.72, 51.74, 56.34, 56.38, 60.97, 102.30, 108.83, 123.80, 127.37, 128.21, 128.68, 128.89, 130.40, 131.85, 135.00, 135.60, 140.10, 150.74, 152.32, 191.62. HR-MS (nES) *m*/*z* calcd [M + H]^+^ 593.9580: observed 593.9578.

### 4.6. Antimicrobial Activity

For determining the antibacterial activities, the synthesized compounds were dissolved in DMSO (20 mg/mL). Broth dilution method in a 96 wells micro-titre plate was used to test the compounds (**27**–**72**) [6]. Mueller Hinton Broth was used as diluents in the wells of the micro-titre plate. The dilutions were prepared at the required quantities of 600, 500, 400, 200, 100, 50, 25, 12.5 µg/mL. Equal volume of bacterial culture was added to it after comparing it with 0.5 McFarland standard and incubated at 37 °C for 16 to 18 h on a rotary shaker at 150 rpm [39]. Plates were then examined for the turbidity as an indicator of growth and the absorbance was taken with the help of ELISA reader at 590 nm [40]. First well that appeared having zero absorbance was taken to be the MIC (Minimum inhibitory concentration) [8]. This was followed by an MTT assay for confirmation. Streptomycin was used as a reference control at concentrations of 50, 25, 12.5, 6.25, 3.12, 1.56, 0.78, 0.39 µg/mL. Streptomycin is a broad spectrum antibiotic which inhibits the growth of both Gram-positive and Gram-negative bacteria and has been widely used as a reference [41,42]. In order to ensure that the solvent had no effect on bacterial growth, a control test was also performed containing inoculated broth supplemented with different concentrations (1%–16%) of DMSO. All the tests were performed in triplicates.

The MBC (minimum bactericidal concentration) was determined by sub culturing the preparations that have shown no evidence of growth on Mueller Hinton Agar in neat and dilutions of 1:10 and 1:100. A colony-forming unit (CFU) count was done for the agar plates and the dilution that gave 99.9% reduction to the original inoculum was considered to be the MBC.

All the compounds were tested for their in vitro growth inhibitory activity against different bacteria. The bacterial strains used were *Bacillus subtilis* ATCC 6633 and *Staphylococcus aureus* ATCC 6538 as Gram-positive bacteria and *Salmonella typhimurium* ATCC 14028 (*Salmonella enterica*) and *Proteus vulgaris* ATCC 13315 as Gram-negative bacteria. *Staphylococcus aureus* is a clinically important pathogen as it causes various types of infections [43] and *Bacillus subtilis* was used as a model organism as it belongs to the same group as pathogenic *Bacillus anthracis* species [44]. *Salmonella typhimurium* is clinically important as it can cause enteritis. *Proteus vulgaris* has been isolated from long term care facilities and hospitals and from immunocompromised patients. The strains used in this study were obtained from culture collection of University of Hertfordshire.

## Figures and Tables

**Table 1:** The general structure of compounds **28**–**72** where R^1^, R^2^ and R^3^ refer to substituents attached to the benzoyl moiety while R^4^ and R^5^ refer to the substituents attached to the thiazine ring.

Compound	R^1^	R^2^	R^3^	R^4^	R^5^
**28**	H	H	H	H	Br
**29**	H	H	H	CH_3_	NHCH_3_
**30**	H	H	H	CH_2_CH_3_	Br
**31**	H	H	H	Ph	Br
**32**	H	H	H	PhCH_2_	Br
**33**	CH_3_	H	H	H	Br
**34**	CH_3_	H	H	CH_3_	NHCH_3_
**35**	CH_3_	H	H	CH_2_CH_3_	Br
**36**	CH_3_	H	H	Ph	Br
**37**	CH_3_	H	H	PhCH_2_	Br
**38**	Cl	H	H	H	Br
**39**	Cl	H	H	CH_3_	NHCH_3_
**40**	Cl	H	H	CH_2_CH_3_	Br
**41**	Cl	H	H	Ph	Br
**42**	Cl	H	H	PhCH_2_	Br
**43**	Br	H	H	H	Br
**44**	Br	H	H	CH_3_	Br
**45**	Br	H	H	CH_2_CH_3_	Br
**46**	Br	H	H	Ph	Br
**47**	Br	H	H	PhCH_2_	Br
**48**	F	H	H	H	H
**49**	F	H	H	CH_3_	Br
**50**	F	H	H	CH_2_CH_3_	Br
**51**	F	H	H	Ph	Br
**52**	F	H	H	PhCH_2_	Br
**53**	H	Cl	H	H	Br
**54**	H	Cl	H	CH_3_	NHCH_3_
**55**	H	Cl	H	CH_2_CH_3_	Br
**56**	H	Cl	H	Ph	Br
**57**	H	Cl	H	PhCH_2_	Br
**58**	H	Br	H	H	Br
**59**	H	Br	H	CH_3_	NHCH_3_
**60**	H	Br	H	CH_2_CH_3_	Br
**61**	H	Br	H	Ph	Br
**62**	H	Br	H	PhCH_2_	Br
**63**	H	H	Cl	H	Br
**64**	H	H	Cl	CH_3_	NHCH_3_
**65**	H	H	Cl	CH_2_CH_3_	Br
**66**	H	H	Cl	Ph	Br
**67**	H	H	Cl	PhCH_2_	Br
**68**	H	H	Br	H	Br
**69**	H	H	Br	CH_3_	NHCH_3_
**70**	H	H	Br	CH_2_CH_3_	Br
**71**	H	H	Br	Ph	Br
**72**	H	H	Br	PhCH_2_	Br

**Table 2:** MIC and MBC (μg/mL) of selected compounds and streptomycin against Gram-positive bacteria *B. subtilis*, *S. aureus* and Gram-negative bacteria *P. vulgaris*, *S. typhimurium* and their Clog *p*-values (For MIC and MBC values in μmol/mL see Supplementary Materials Table S1).

Compound	*B. subtilis*		*S. aureus*		*P. vulgaris*		*S. typhimurium*		Clog P
	MIC	MBC	MIC	MBC	MIC	MBC	MIC	MBC	
**31**	500	500	500	>600	>600	>600	>600	>600	4.668
**33**	50	50	100	>600	>600	>600	>600	>600	3.316
**38**	50	50	100	>600	>600	>600	>600	>600	3.596
**43**	25	50	100	-	>600	>600	>600	>600	3.746
**45**	500	500	500	-	>600	>600	>600	>600	4.811
**50**	500	600	400	>600	>600	>600	>600	>600	4.091
**53**	100	100	100	200	>600	>600	>600	>600	3.596
**55**	100	100	200	>600	>600	>600	>600	>600	4.661
**58**	25	50	100	200	>600	>600	>600	>600	3.746
**60**	600	>600	400	400	>600	>600	>600	>600	4.811
**63**	>600	>600	400	>600	>600	>600	>600	>600	3.336
**68**	>600	>600	500	>600	>600	>600	>600	>600	3.486
Streptomycin	12.5	12.5	12.5	25	6.25	6.25	100	100	

## Supplementary Materials

Supplementary materials can be accessed at: http://www.mdpi.com/1420-3049/21/7/861/s1.
