# Supplementary material for: Synthesis and Antimicrobial Activity of 1,2-Benzothiazine Derivatives
Source: Molecules. 2016 Jun 30;21(7):861. doi: 10.3390/molecules21070861 (PMC6274075; doi:10.3390/molecules21070861)
Supplement: Supplementary file 1 [file molecules-21-00861-s001.pdf]

Supplementary Materials: Synthesis and Antimicrobial Activity of 1,2-Benzothiazine Derivatives

Chandani Patel, Jatinder P. Bassin, Mark Scott, Jenna Flye, Ann P. Hunter Lee Martin and Madhu Goyal

Content

^{1}H-NMR and ^{13}C-NMR and HSMS data of representative compounds and micro data.

^{1}H-NMR spectrum for **30** in $CDCl_3$
^{13}C-NMR spectrum for **30** in $CDCl_3$
^{1}H-NMR spectrum for **33** in $CDCl_3$
^{13}C-NMR spectrum for **33** in $CDCl_3$
^{1}H-NMR spectrum for **39** in $CDCl_3$
^{13}C-NMR spectrum for **39** in $CDCl_3$
^{1}H-NMR spectrum for **47** in $CDCl_3$
^{13}C-NMR spectrum for **47** in $CDCl_3$
^{1}H-NMR spectrum for **52** in $CDCl_3$
^{13}C-NMR spectrum for **52** in $CDCl_3$
^{1}H-NMR spectrum for **56** in $CDCl_3$
^{13}C-NMR spectrum for **56** in $CDCl_3$
^{1}H-NMR spectrum for **62** in $CDCl_3$
^{13}C-NMR spectrum for **62** in $CDCl_3$
^{1}H-NMR spectrum for **67** in $CDCl_3$
^{13}C-NMR spectrum for **67** in $CDCl_3$
^{1}H-NMR spectrum for **71** in $CDCl_3$
^{13}C-NMR spectrum for **71** in $CDCl_3$
^{1}H-NMR spectrum for **31** in $CDCl_3$
^{13}C-NMR spectrum for **31** in $CDCl_3$
^{1}H-NMR spectrum for **35** in $CDCl_3$
^{13}C-NMR spectrum for **35** in $CDCl_3$
^{1}H-NMR spectrum for **44** in $CDCl_3$
^{13}C-NMR spectrum for **44** in $CDCl_3$
^{1}H-NMR spectrum for **49** in $CDCl_3$
^{13}C-NMR spectrum for **49** in $CDCl_3$
^{1}H-NMR spectrum for **54** in $CDCl_3$
^{13}C-NMR spectrum for **54** in $CDCl_3$
^{1}H-NMR spectrum for **60** in $CDCl_3$
^{13}C-NMR spectrum for **60** in $CDCl_3$
^{1}H-NMR spectrum for **66** in $CDCl_3$
^{13}C-NMR spectrum for **66** in $CDCl_3$
^{1}H-NMR spectrum for **69** in $CDCl_3$
^{13}C-NMR spectrum for **69** in $CDCl_3$

HS-MS for **28**
HS-MS for **31**
HS-MS for **35**
HS-MS for **38**
HS-MS for **42**
HS-MS for **46**
HS-MS for **50**
HS-MS for **54**
HS-MS for **57**
HS-MS for **60**
HS-MS for **64**
HS-MS for **67**
HS-MS for **29**
HS-MS for **32**
HS-MS for **36**
HS-MS for **39**
HS-MS for **43**
HS-MS for **47**
HS-MS for **51**
HS-MS for **55**
HS-MS for **58**
HS-MS for **61**
HS-MS for **65**
HS-MS for **69**
HS-MS for **30**
HS-MS for **34**
HS-MS for **37**
HS-MS for **41**
HS-MS for **45**
HS-MS for **49**
HS-MS for **52**
HS-MS for **56**
HS-MS for **59**
HS-MS for **62**
HS-MS for **66**
HS-MS for **71**

Figure 1
Figure 2
Figure 3
Figure 4
Figure 5
Figure 6

Table S1

Compound **30** Proton NMR in $CDCl_3$

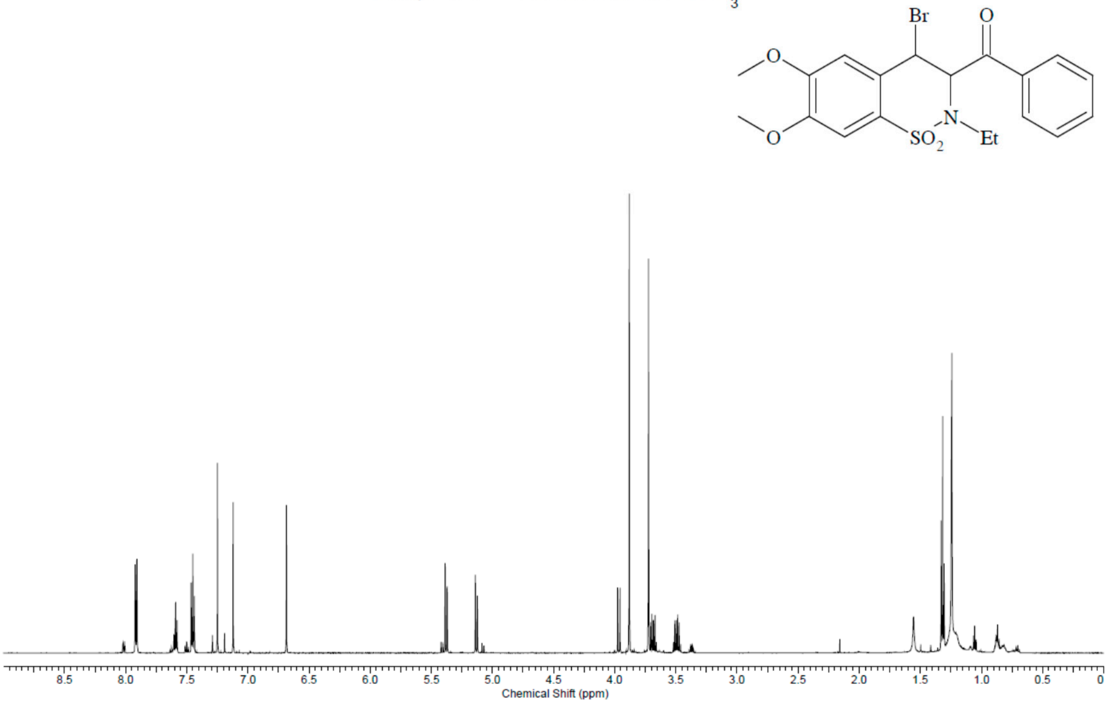

Compound **30** Carbon NMR in $CDCl_3$

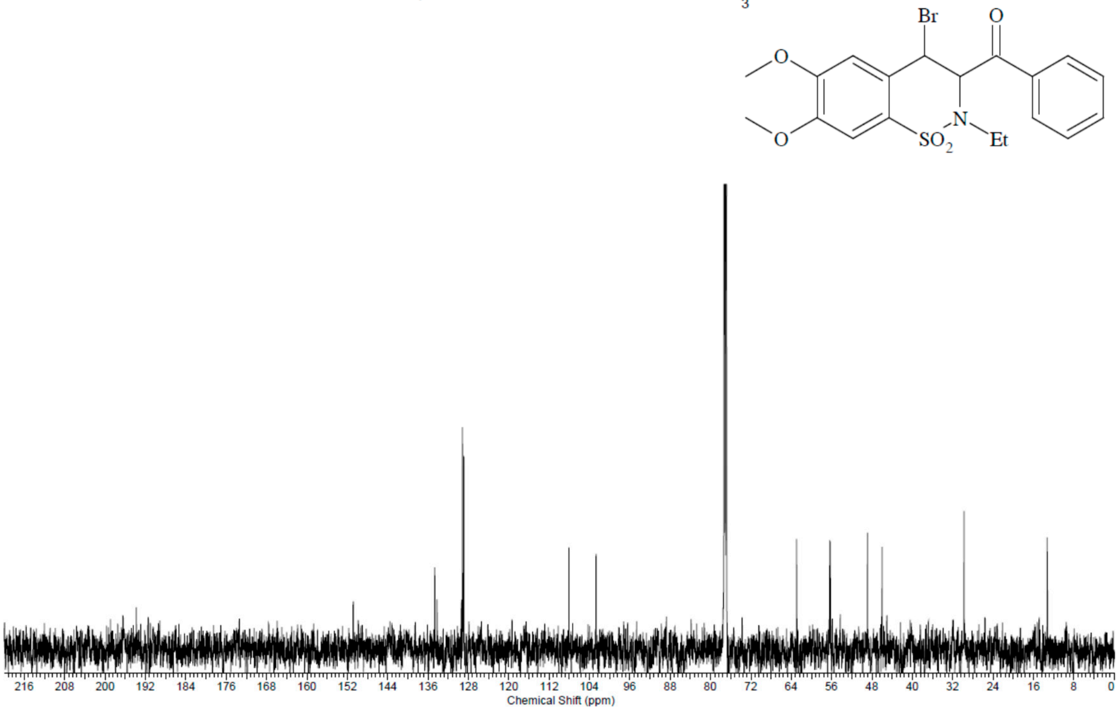

Compound **31** Proton NMR in CDCl3

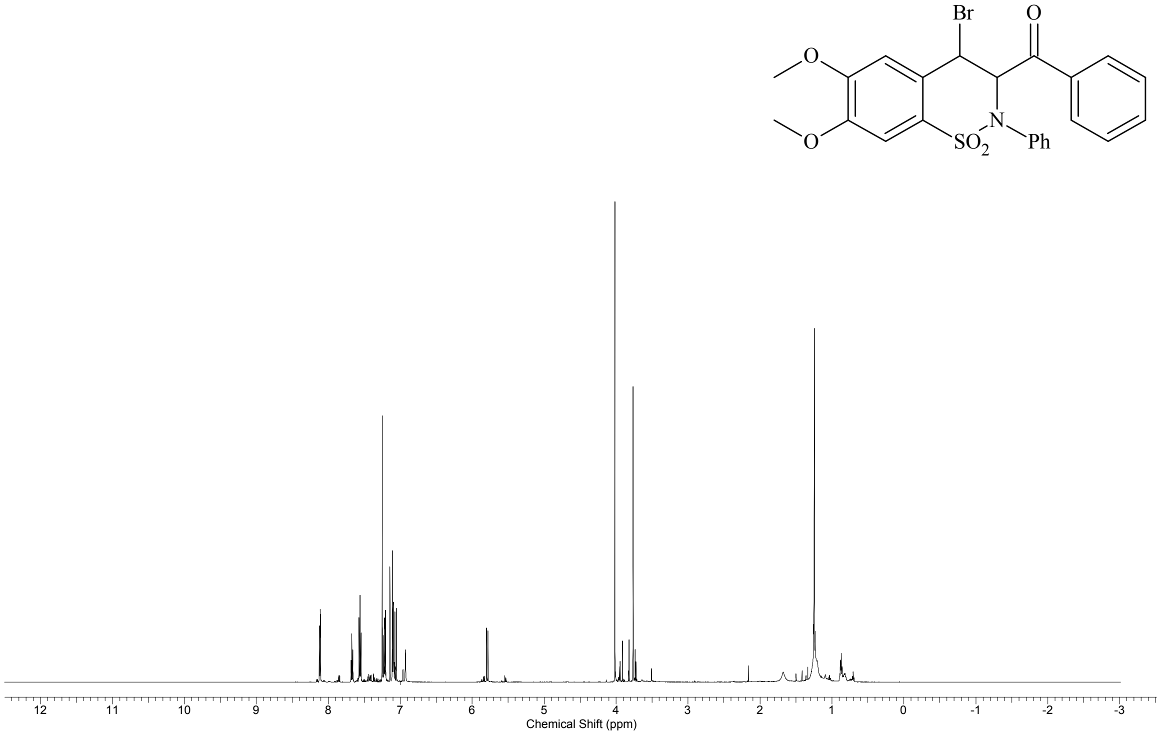

Compound 31 Carbon NMR in CDCl3

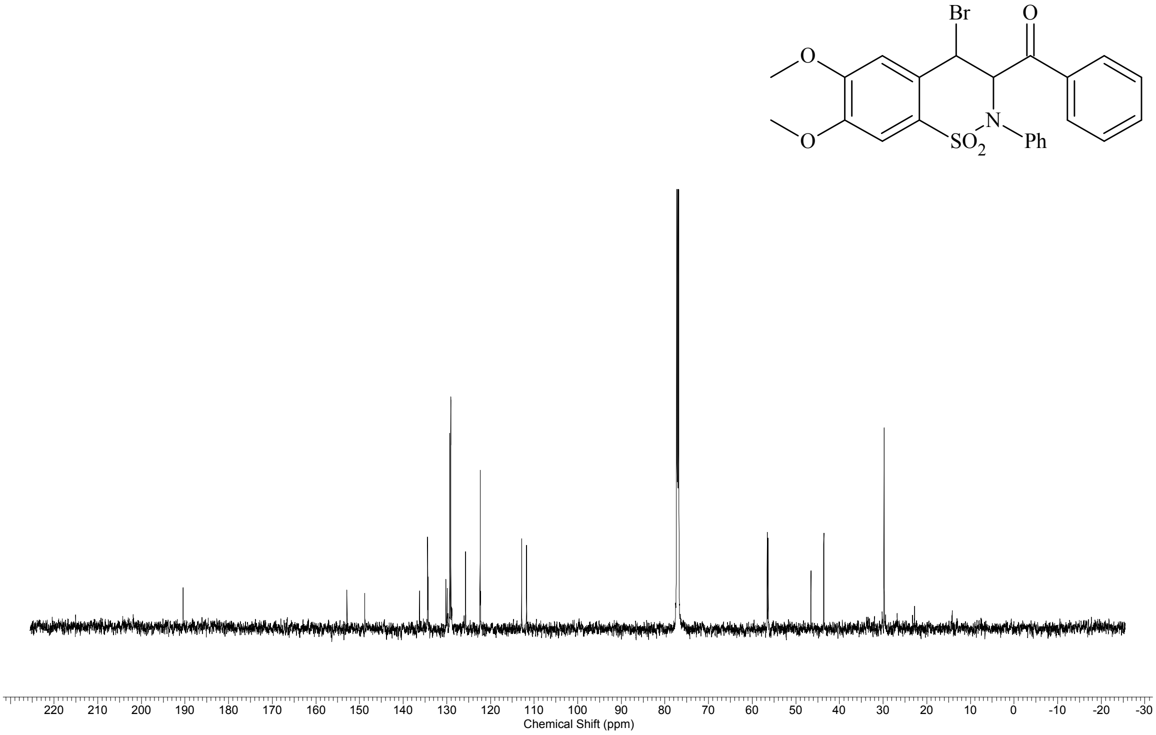

Compound **33** Proton NMR in $CDCl_3$

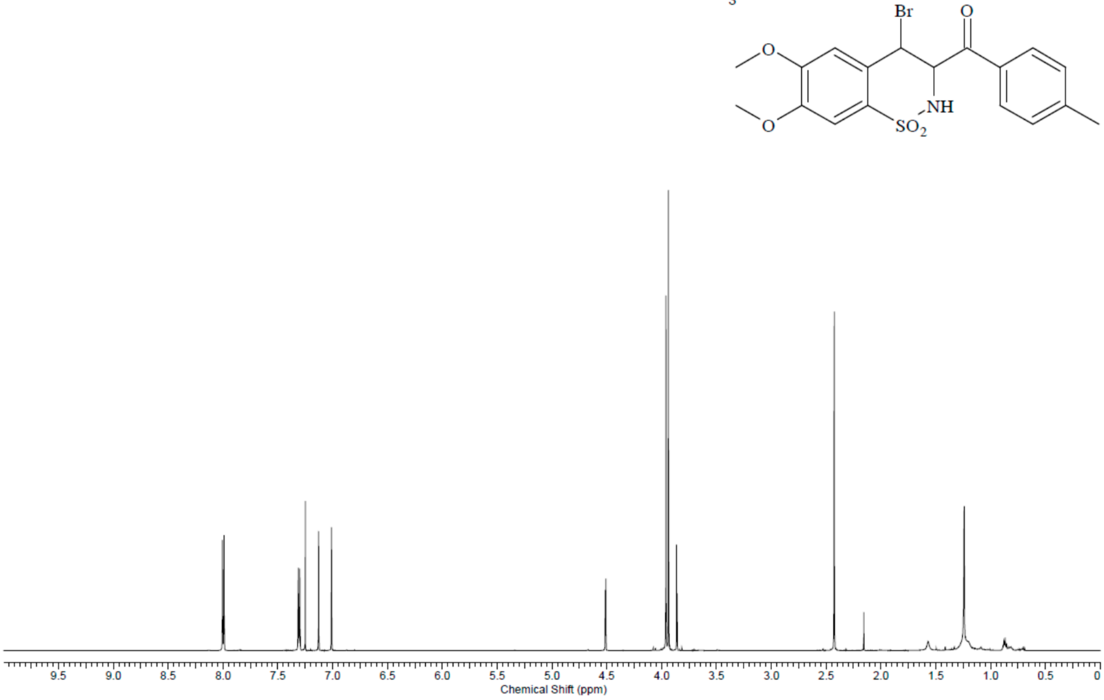

Compound **33** Carbon NMR in $CDCl_3$

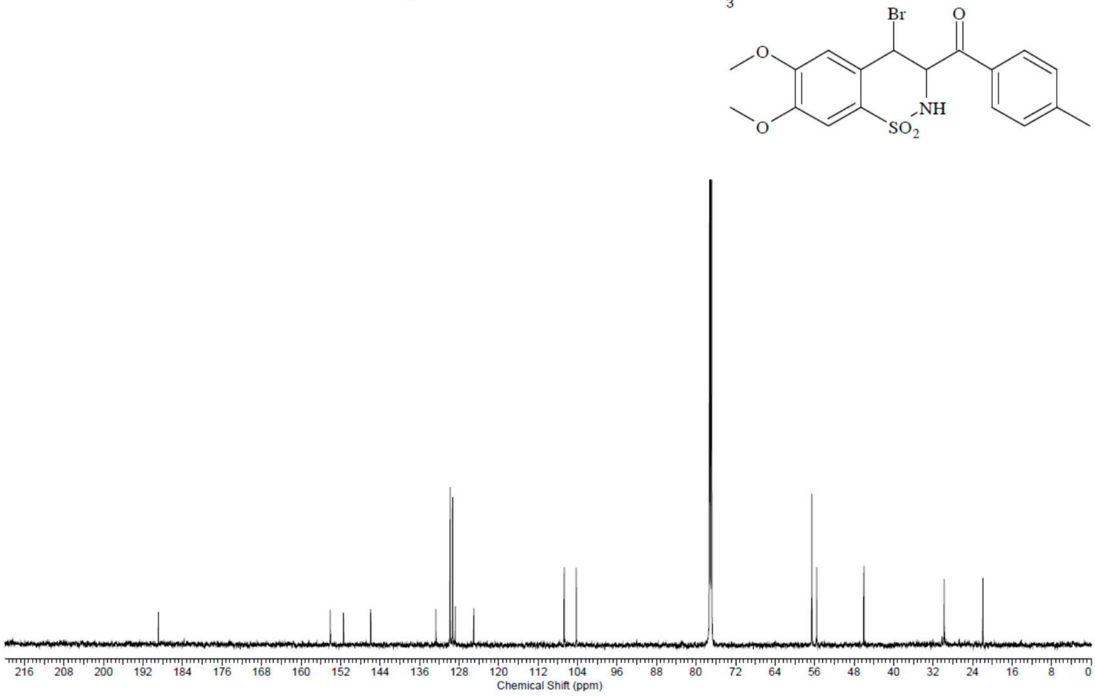

Compound **35** Proton NMR in $CDCl_3$

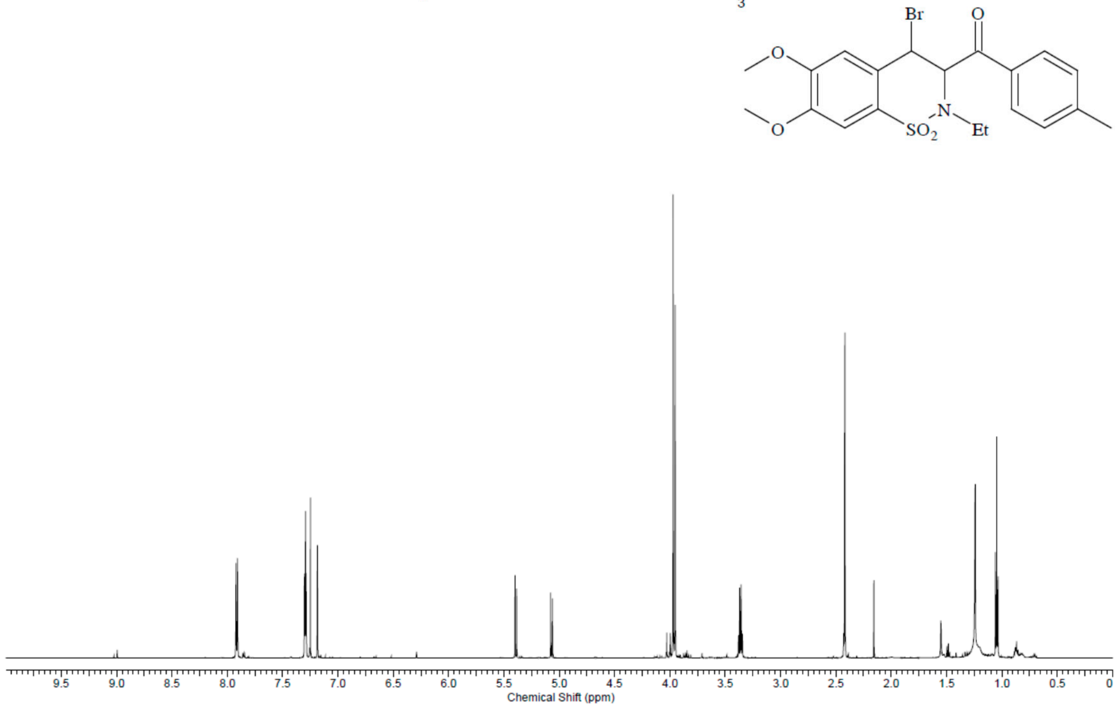

Compound **35** Carbon NMR in $CDCl_3$

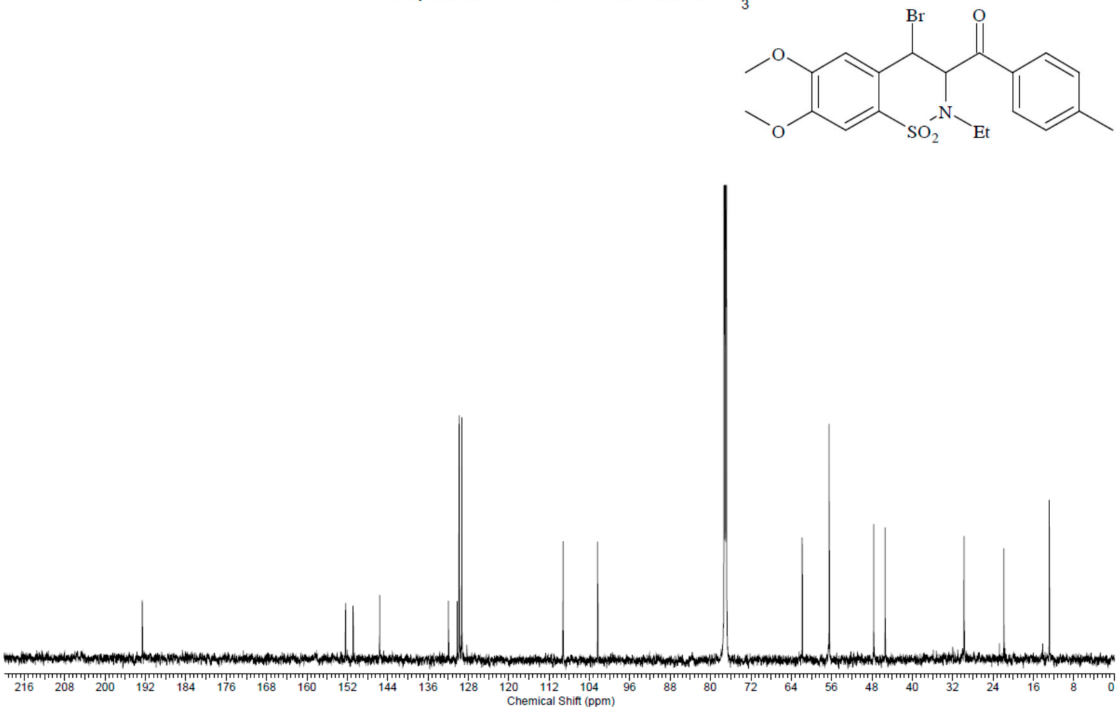

Compound 39 Proton NMR in CDCl3

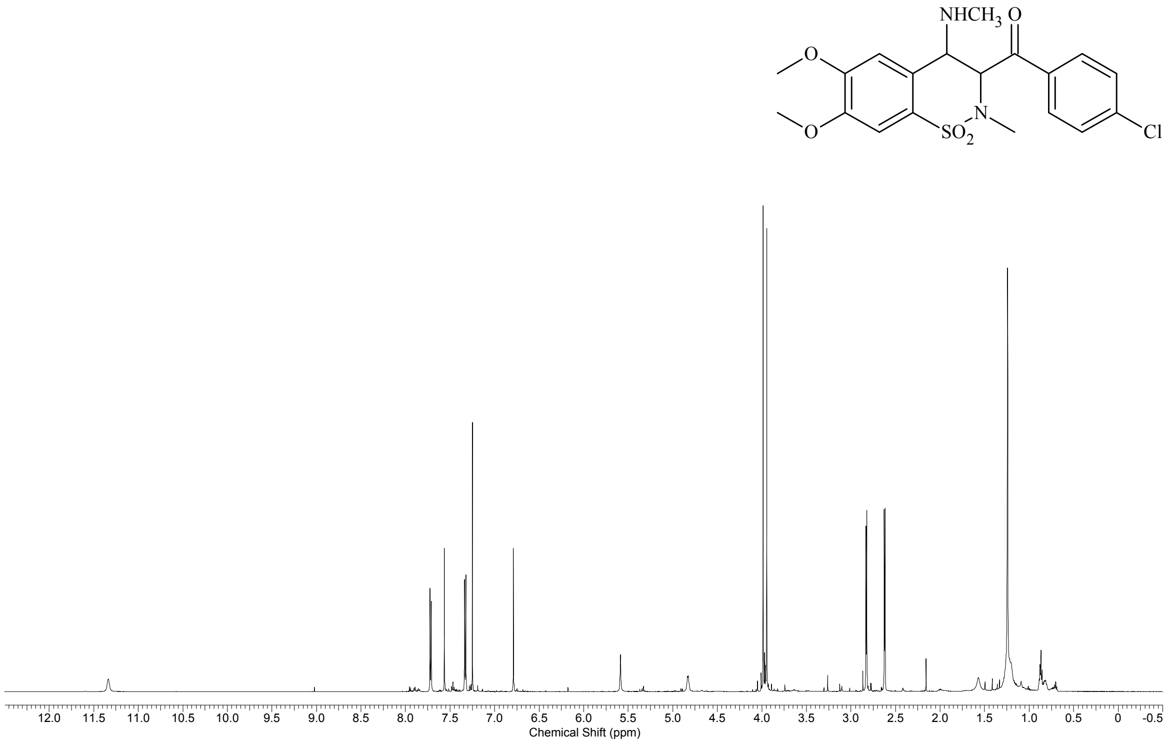

Compound 39 Carbon NMR in CDCl3

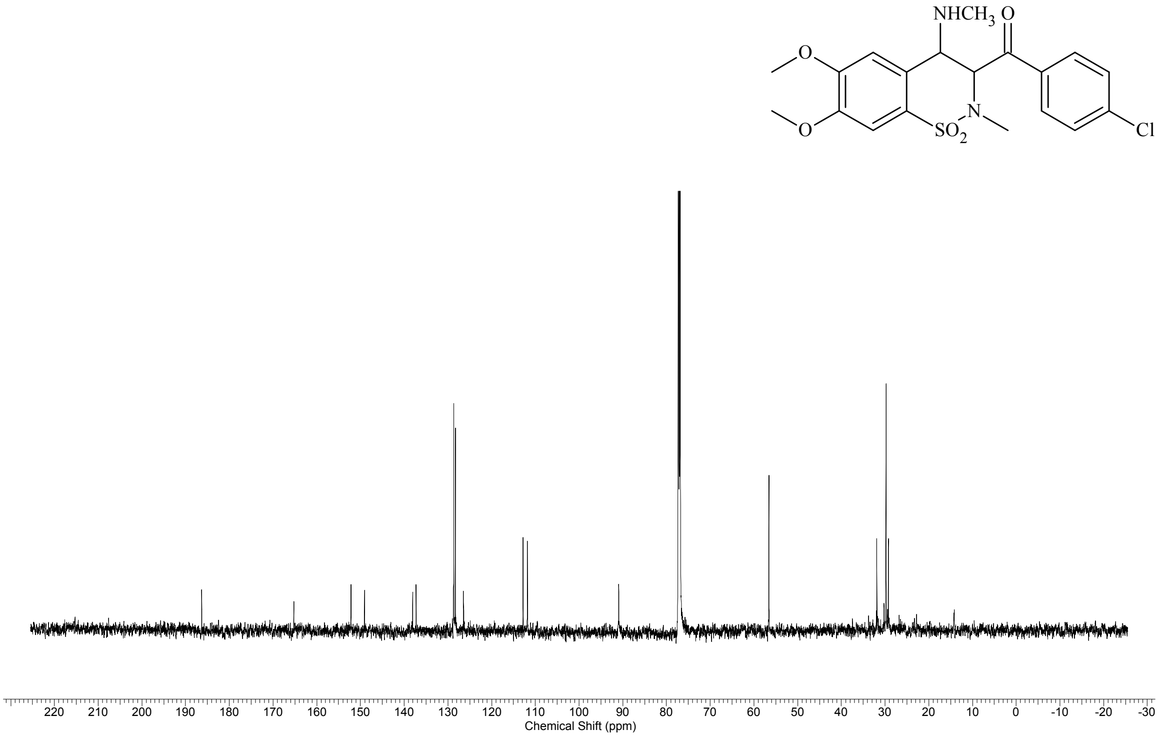

Compound **44** Proton NMR in $CDCl_3$

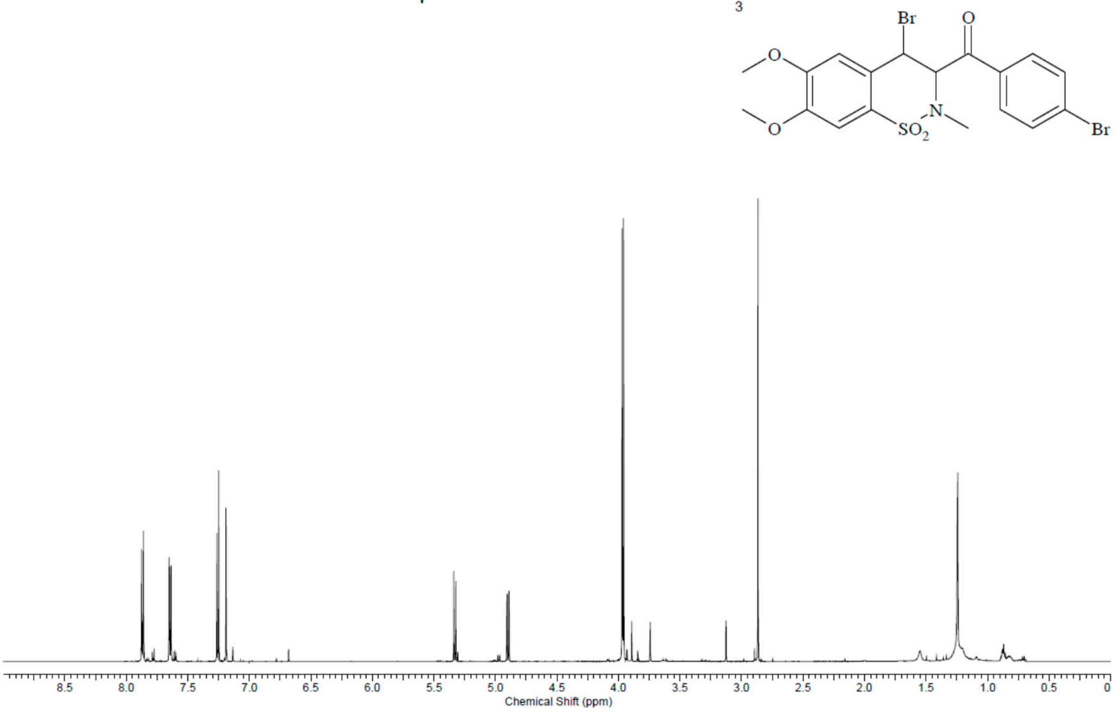

Compound **44** Carbon NMR in $CDCl_3$

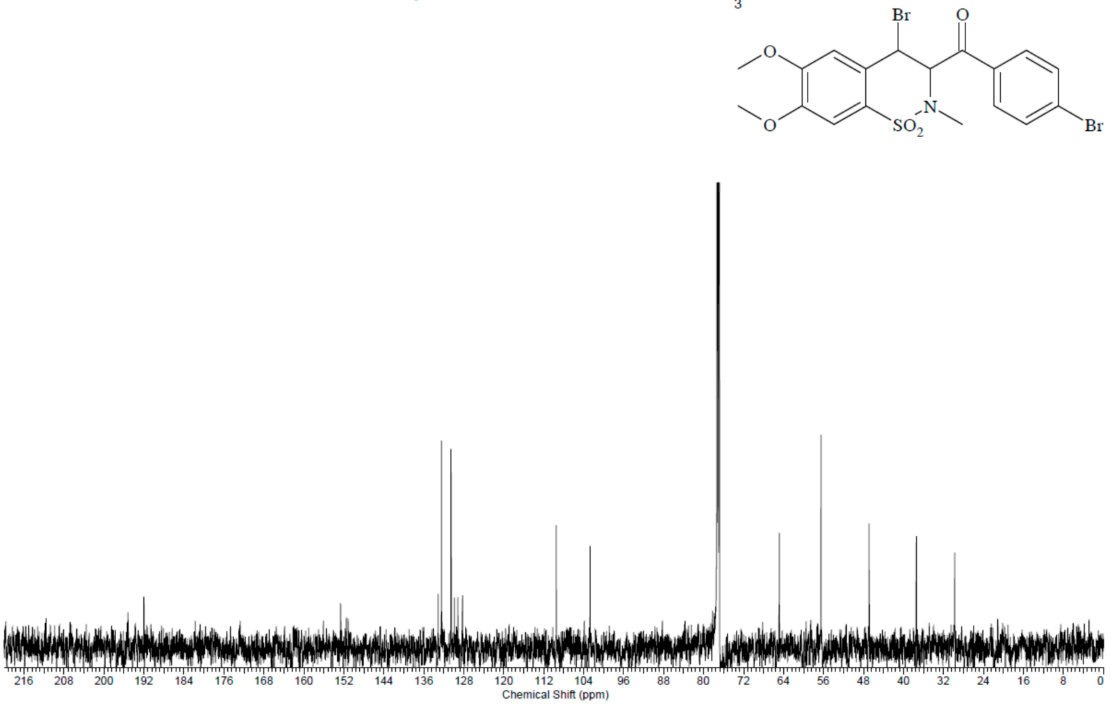

Compound **47** Proton NMR in $CDCl_3$

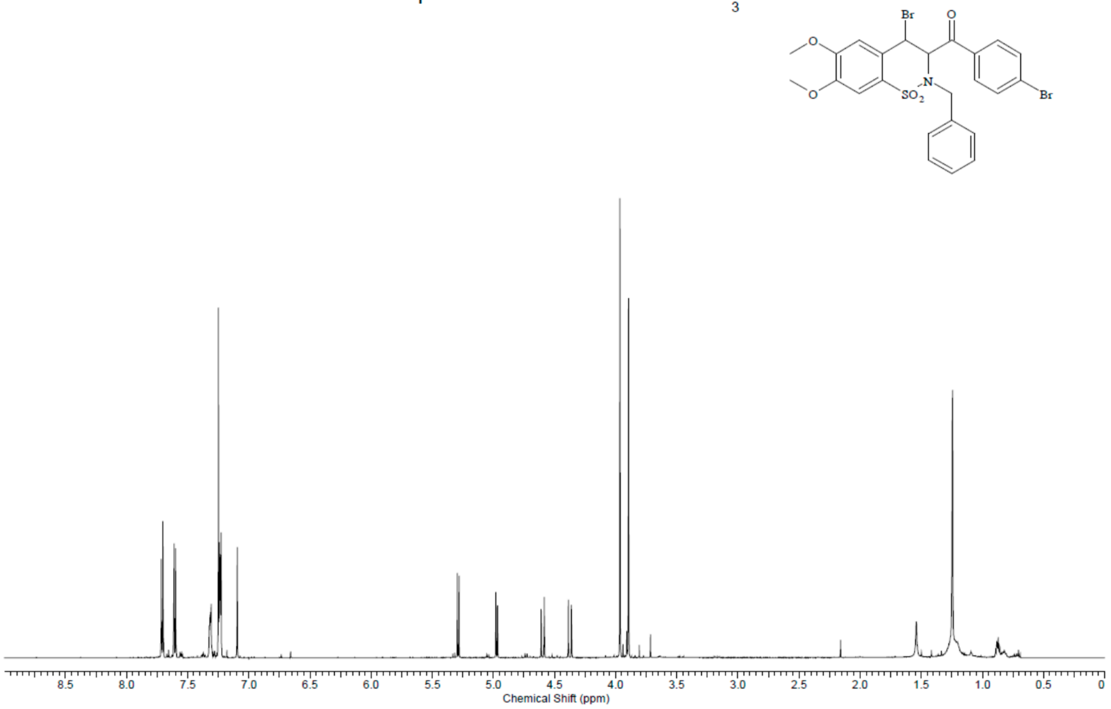

Compound **47** Carbon NMR in $CDCl_3$

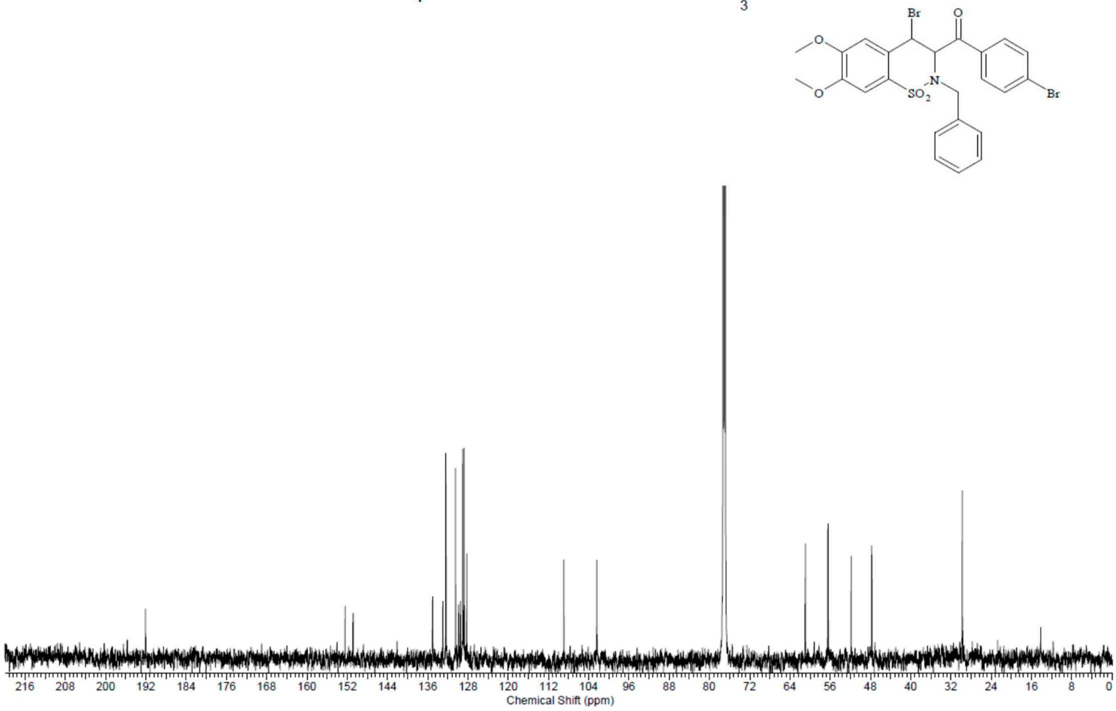

Compound **49** Proton NMR in $CDCl_3$

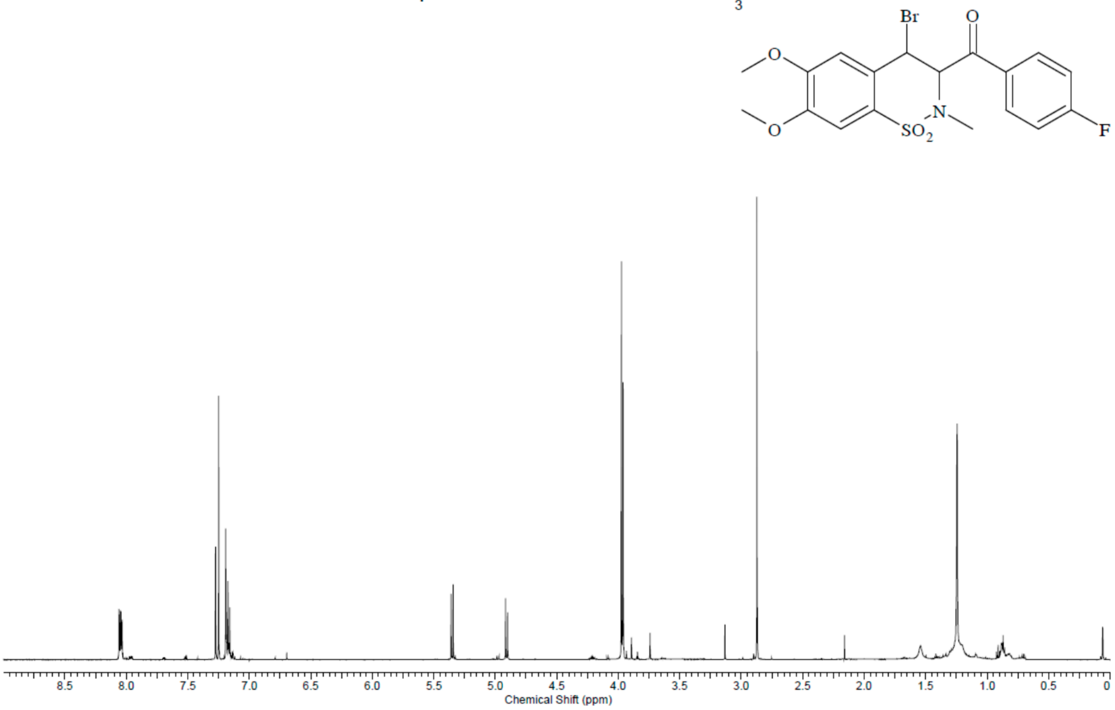

Compound **49** Carbon NMR in $CDCl_3$

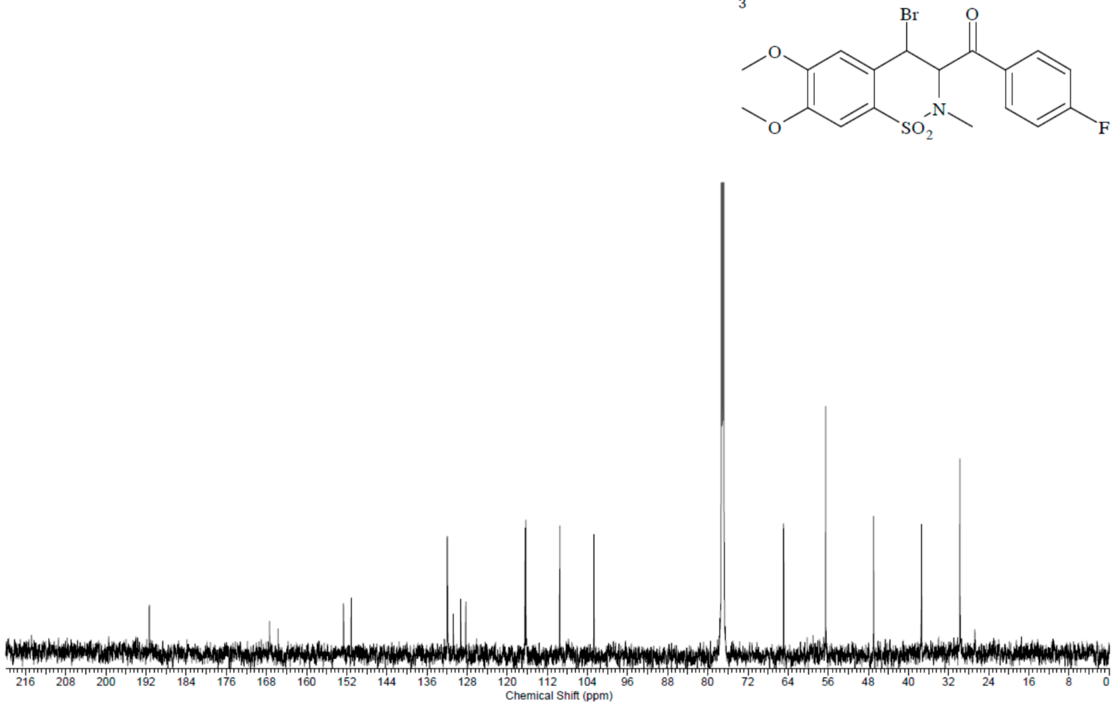

Compound **52** Proton NMR in CDCl3

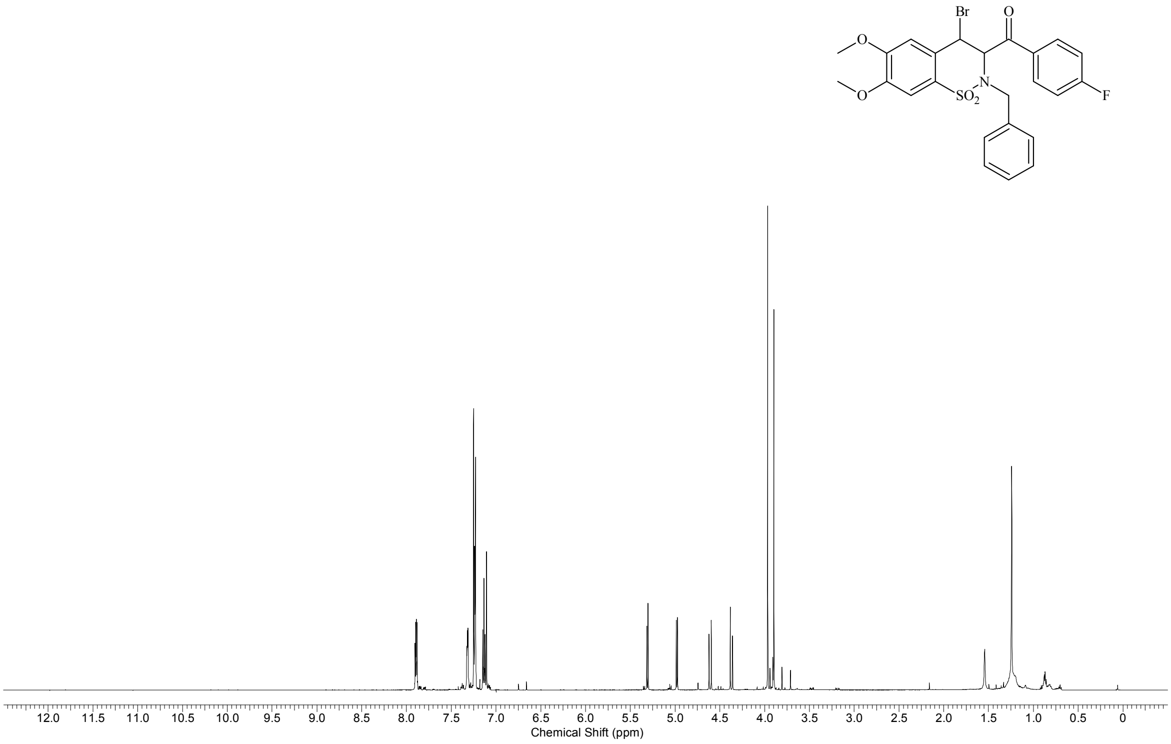

Compound **52** Carbon NMR in $CDCl_3$

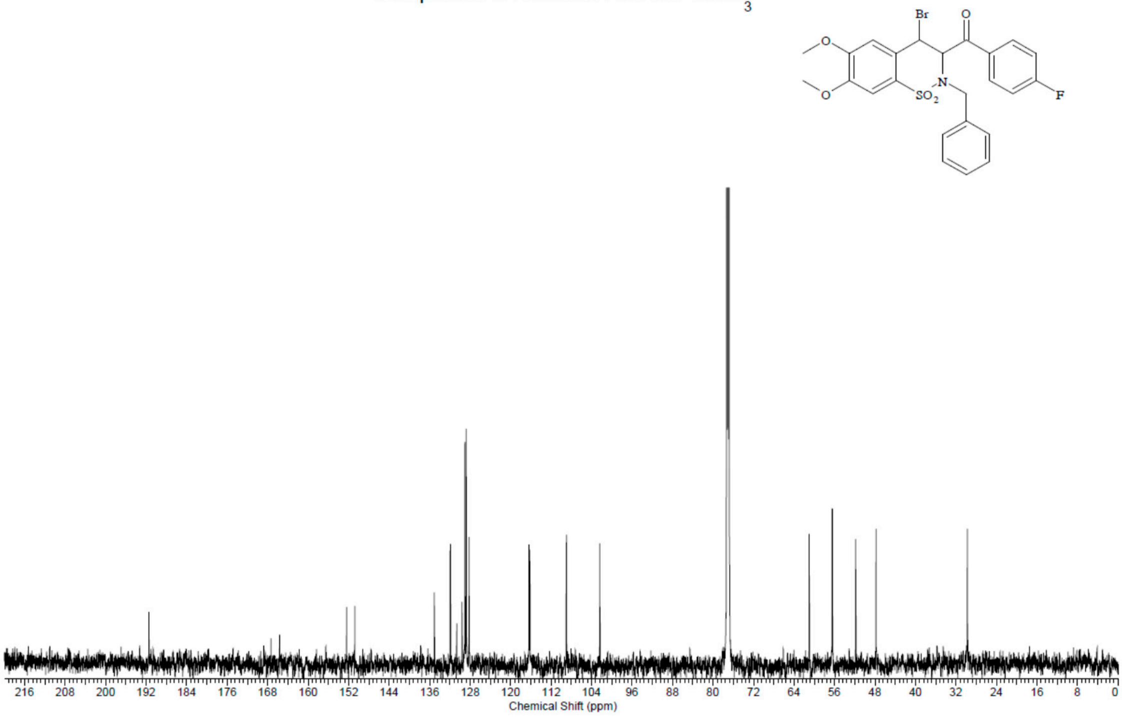

Compound **54** Proton NMR in CDCl3

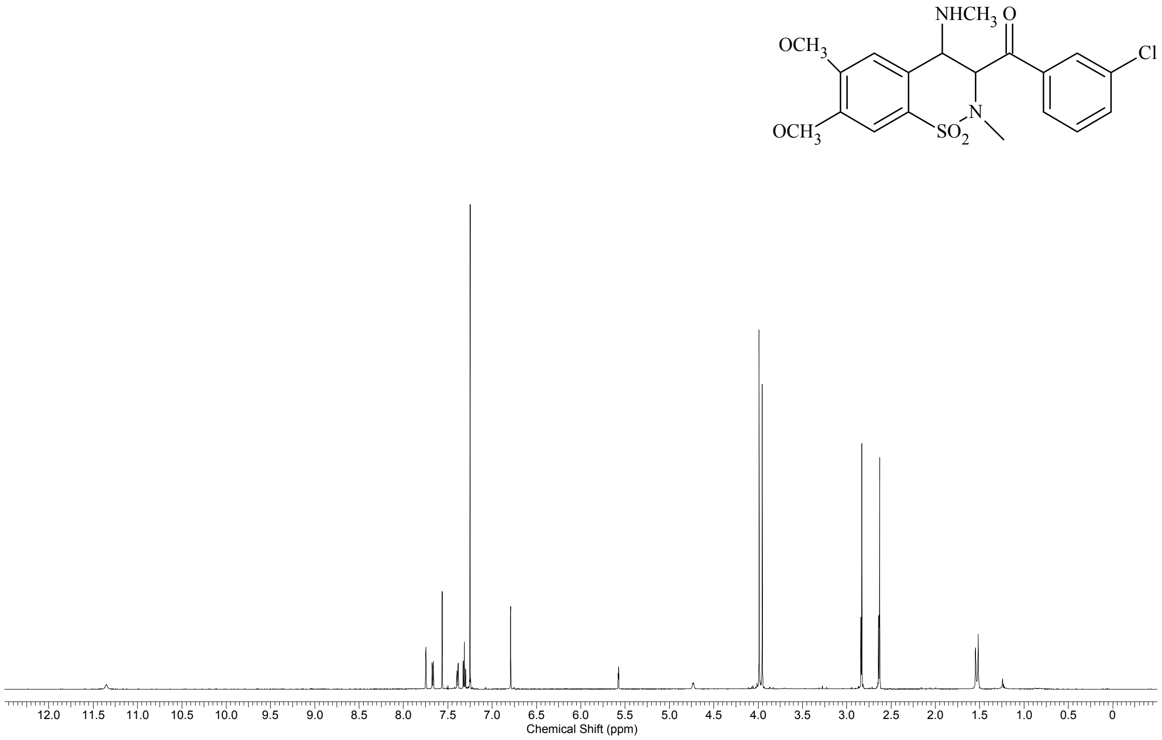

Compound 54 **Carbon NMR in CDCl3**

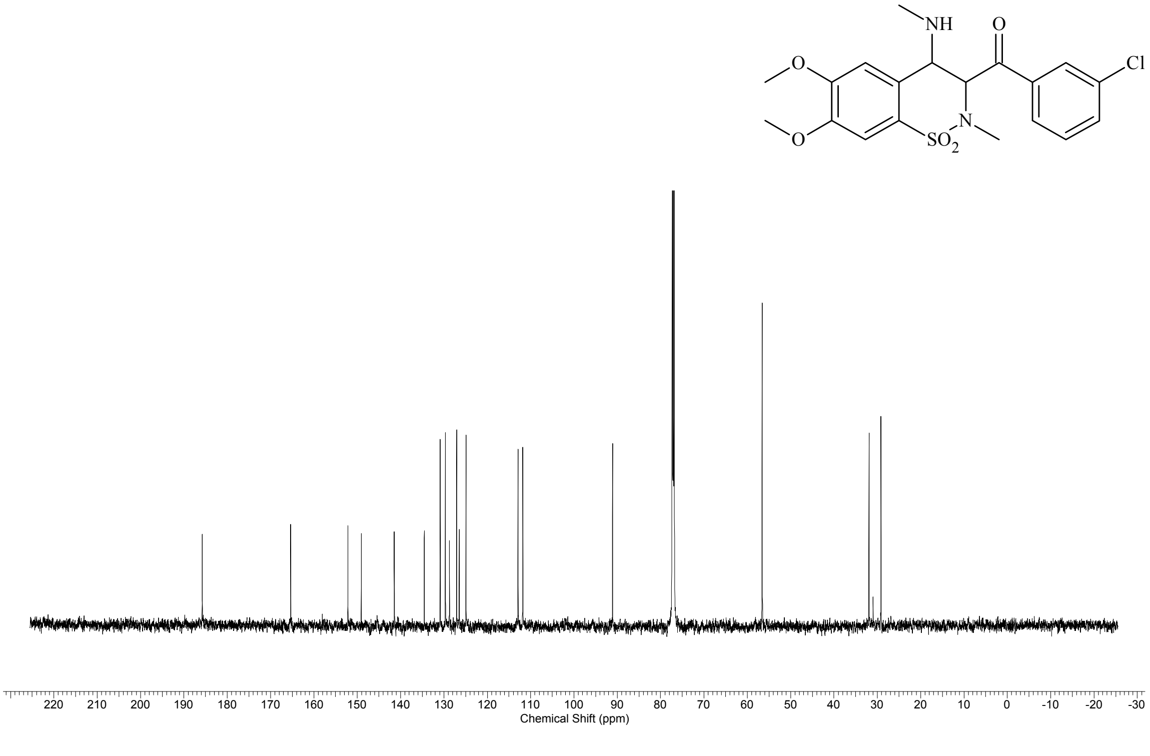

Compound **56** Proton NMR in $CDCl_3$

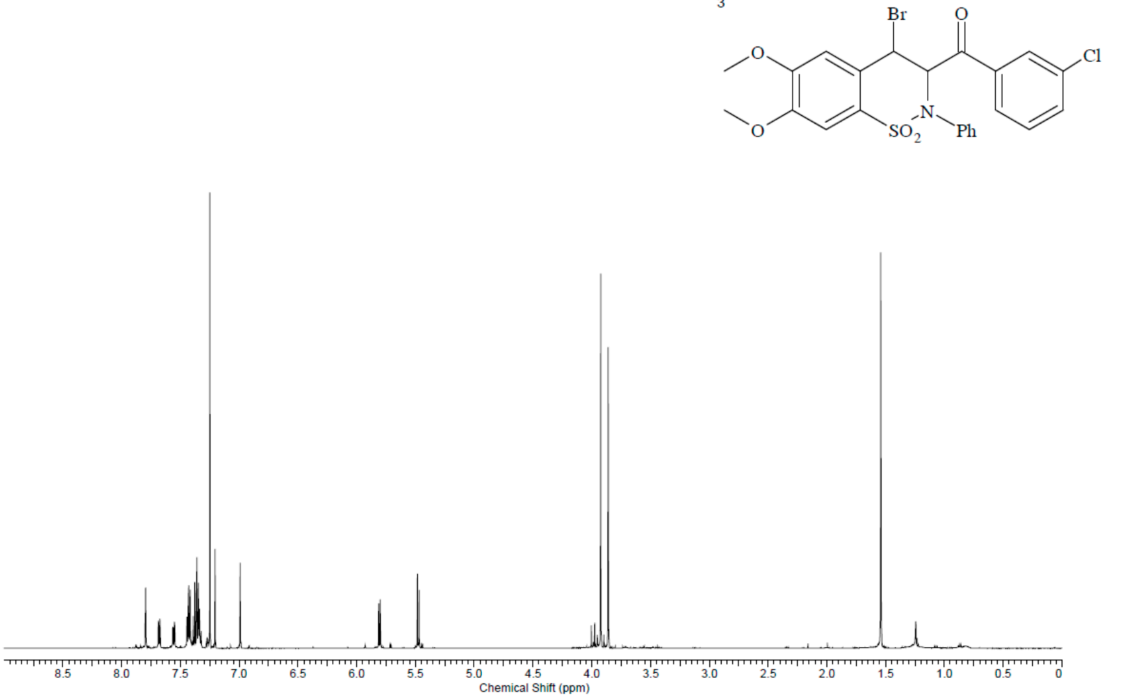

Compound **56** Carbon NMR in $CDCl_3$

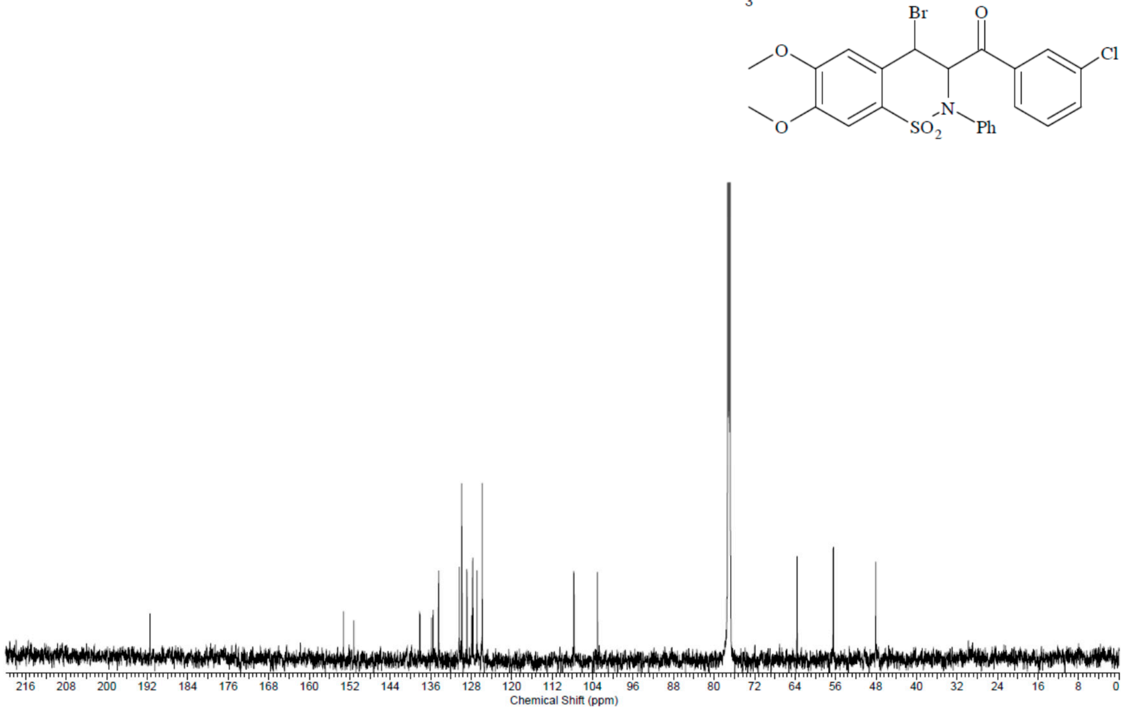

Compound **60** Proton NMR in $CDCl_3$

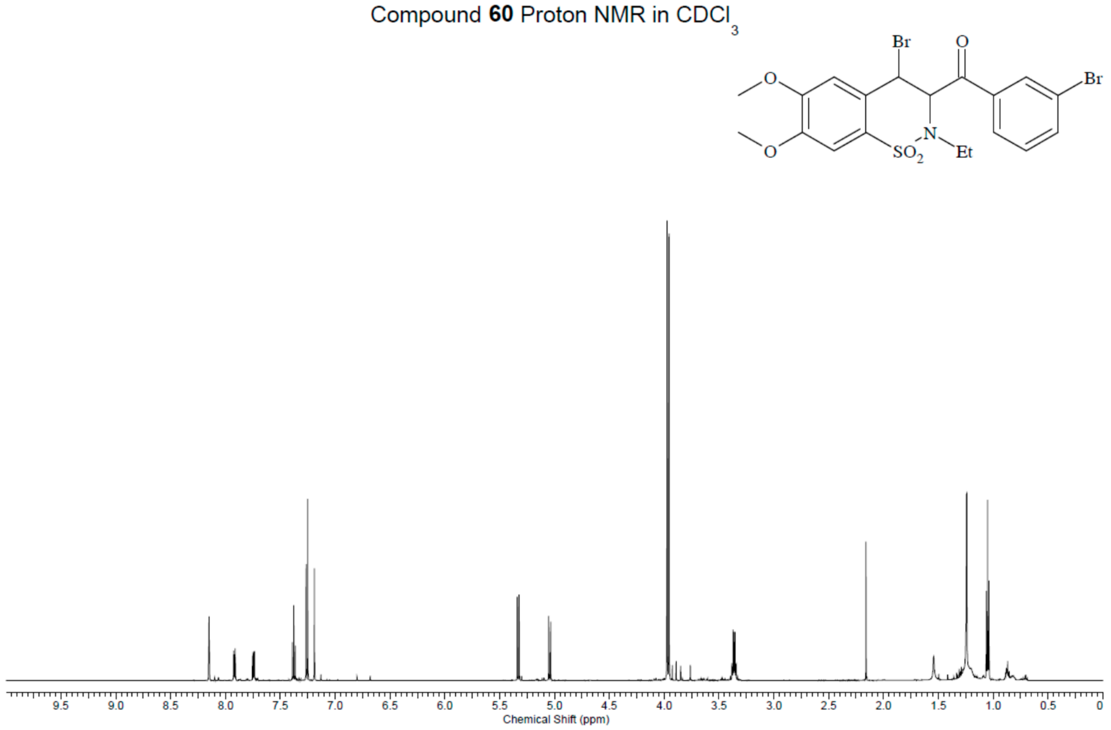

Compound **60** Carbon NMR in $CDCl_3$

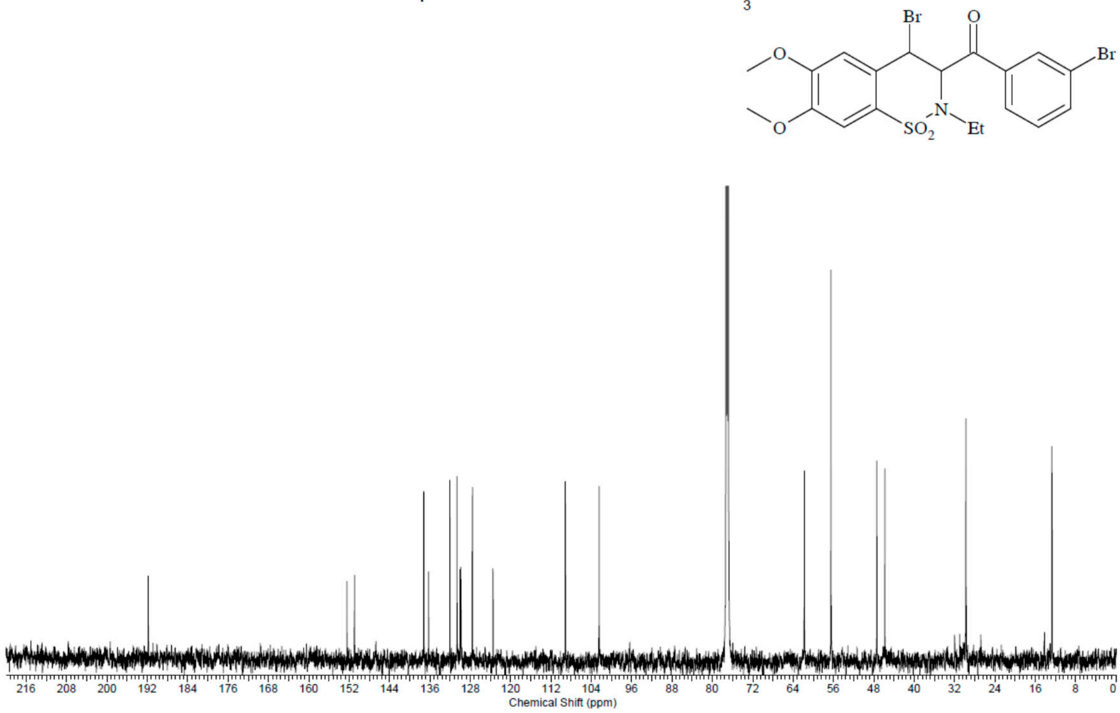

Compound **62** Proton NMR in $CDCl_3$

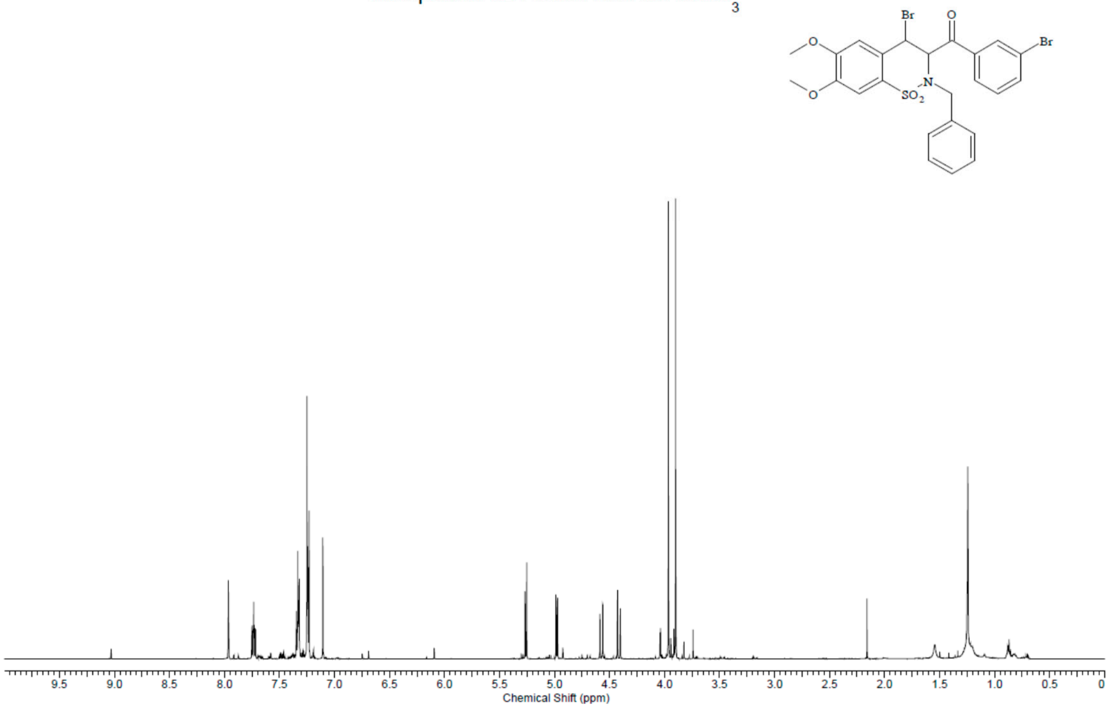

Compound **62** Carbon NMR in $CDCl_3$

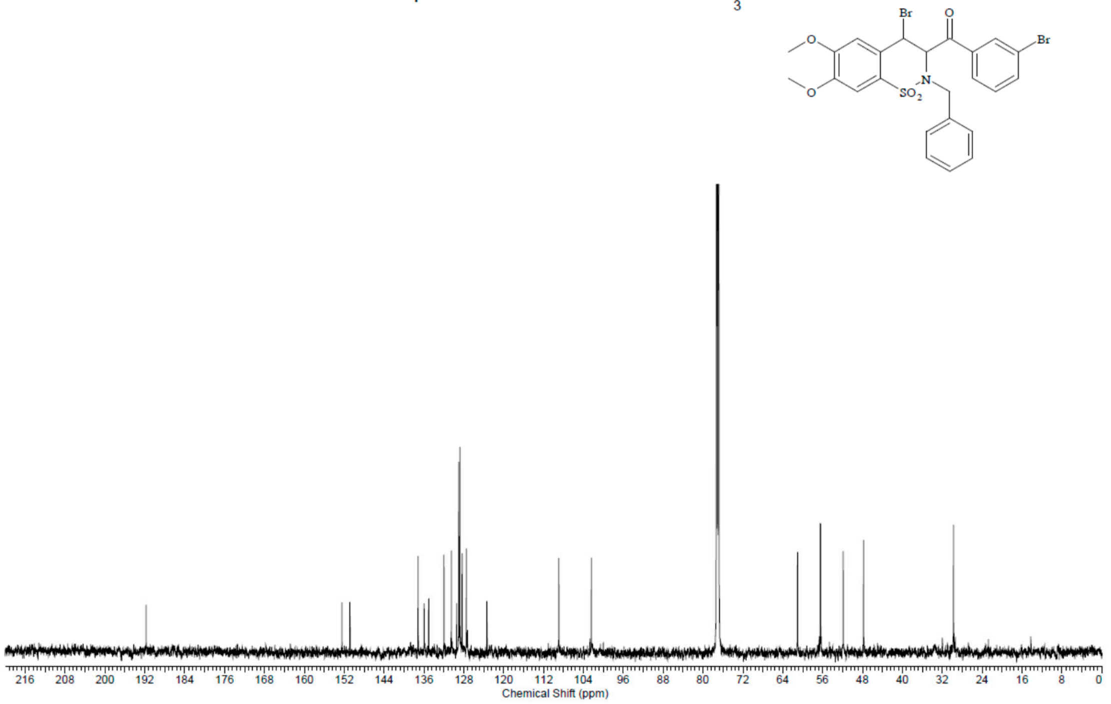

Compound **66** Proton NMR in $CDCl_3$

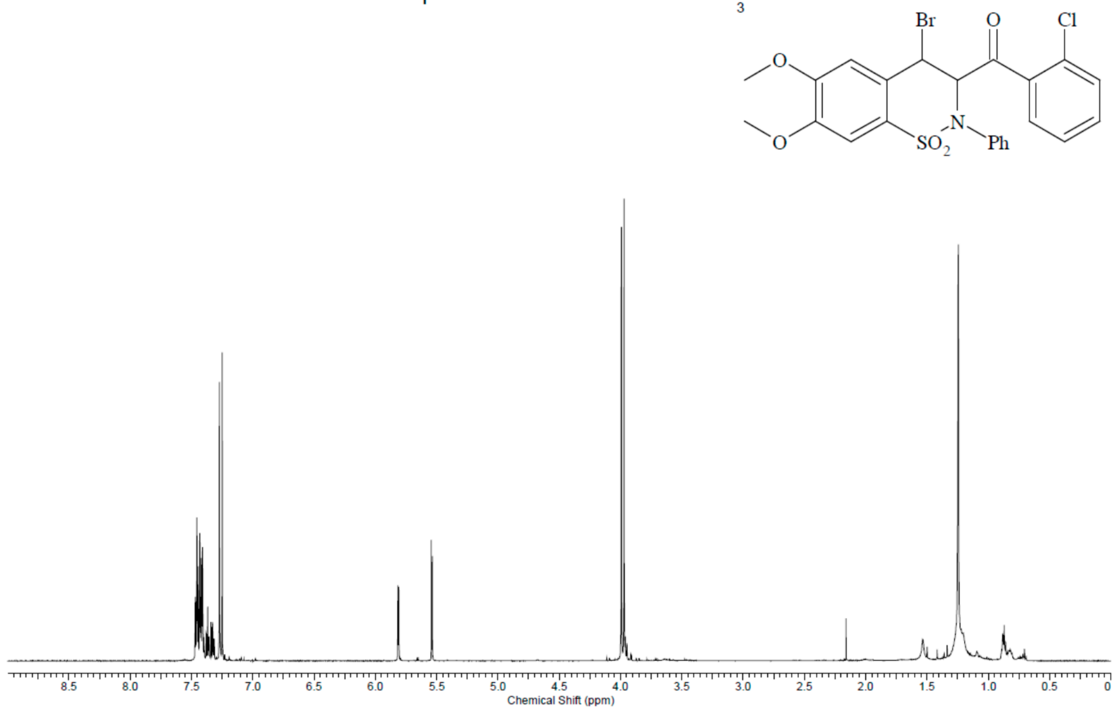

Compound **66** Carbon NMR in $CDCl_3$

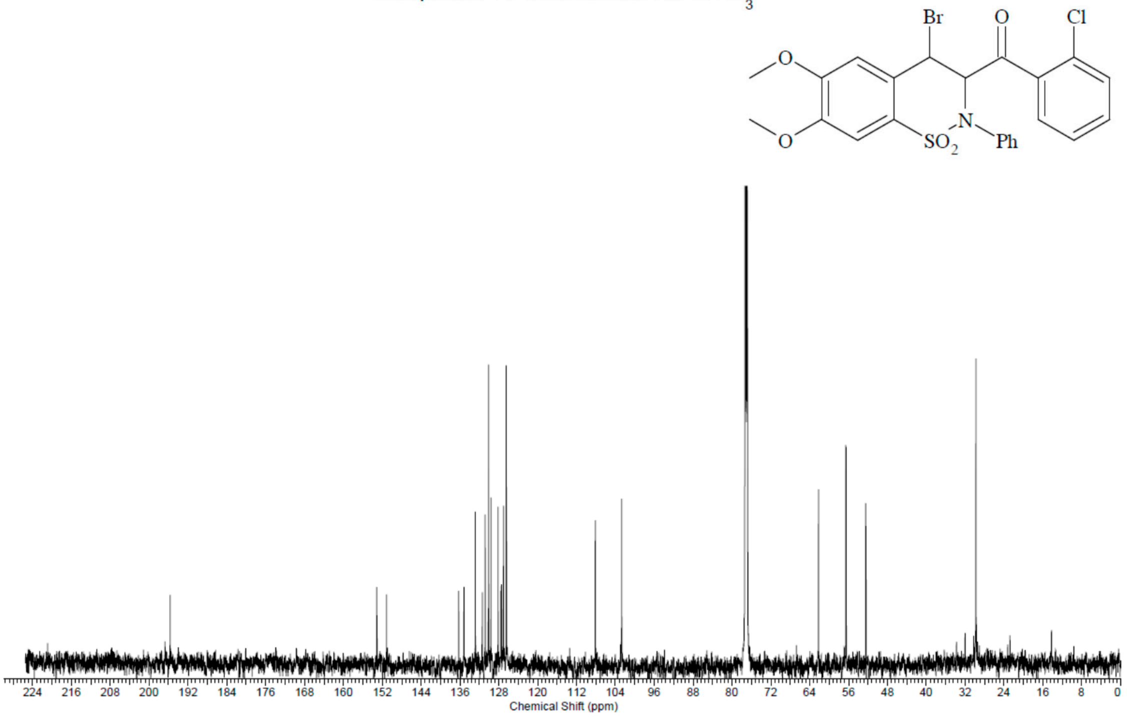

Compound **67** Proton NMR in $CDCl_3$

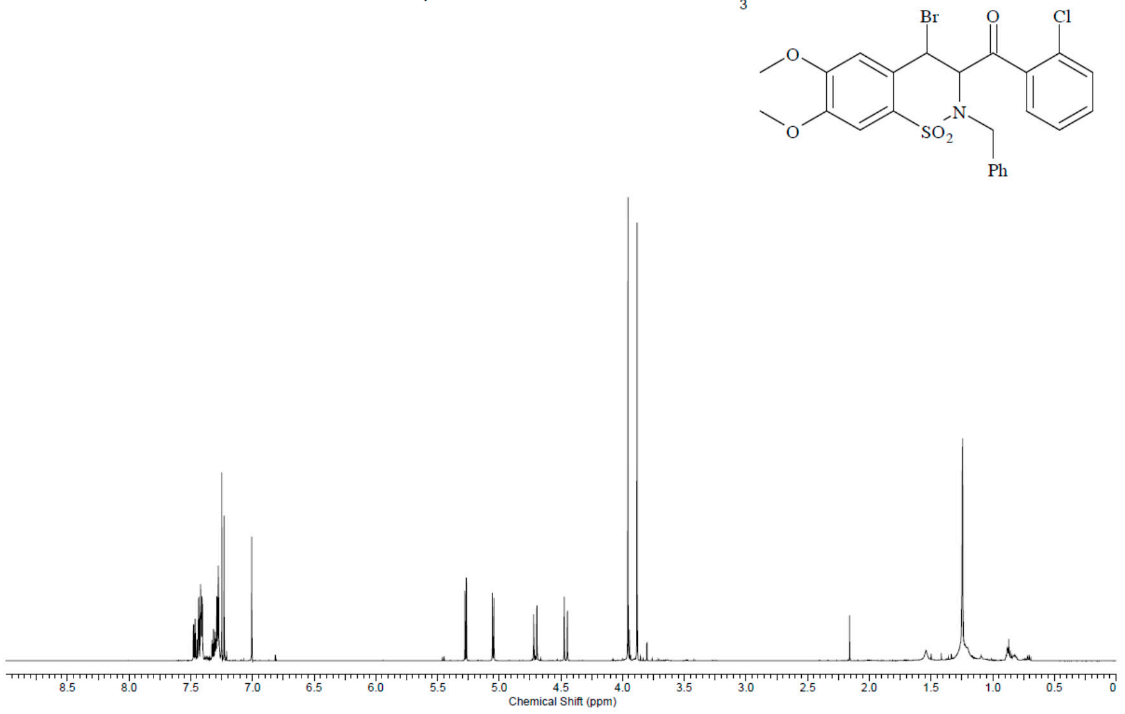

Compound **67** Carbon NMR in $CDCl_3$

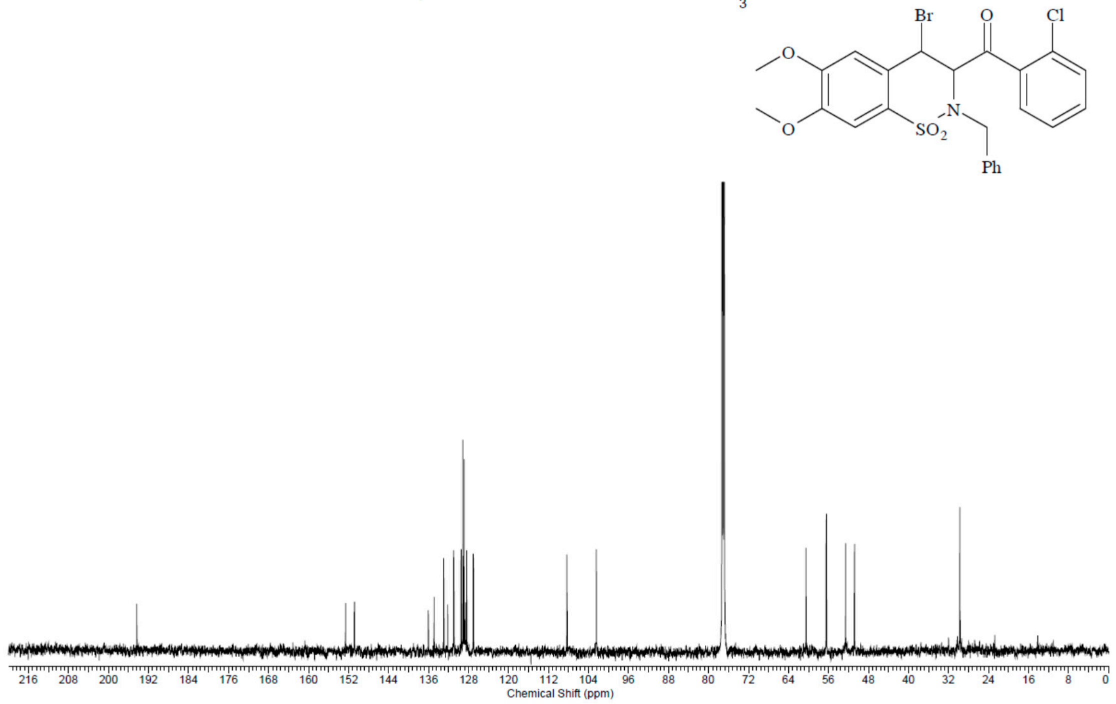

Compound **69** Proton NMR in $CDCl_3$

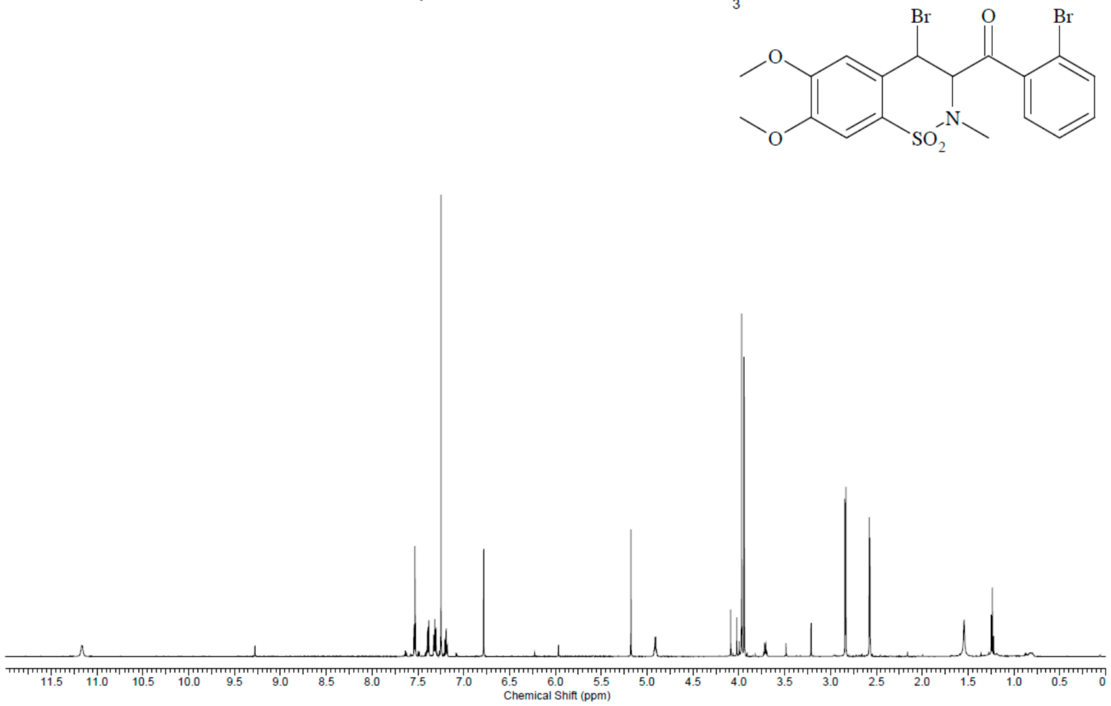

Compound **69** Carbon NMR in $CDCl_3$

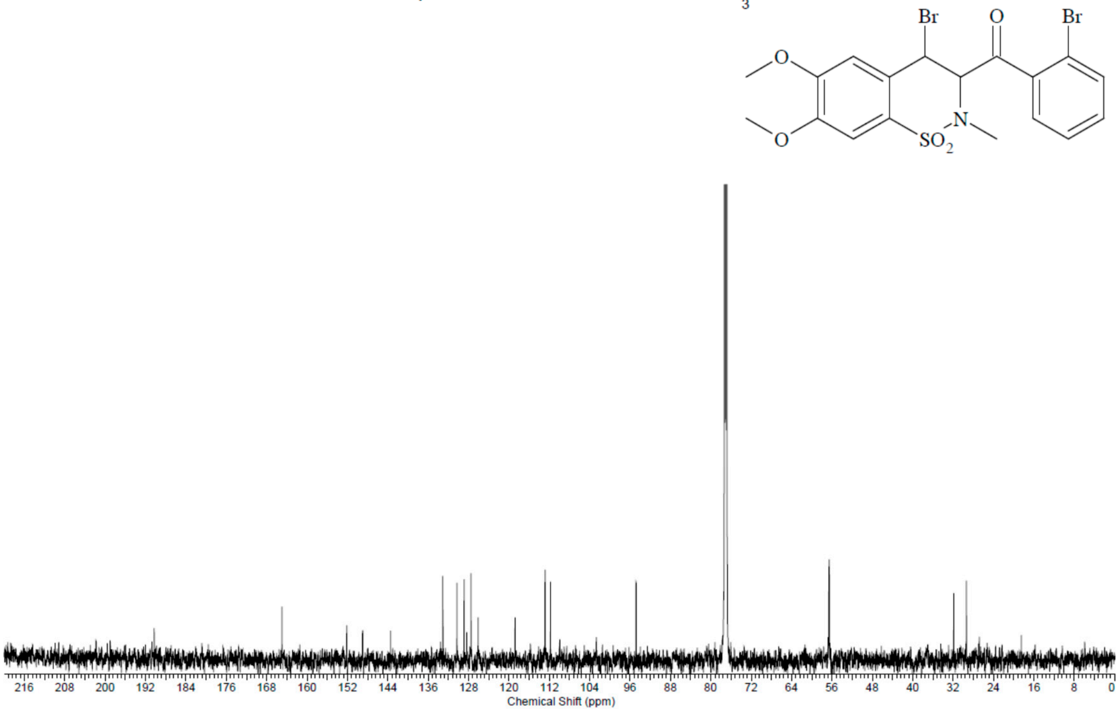

Compound **71** Proton NMR in $CDCl_3$

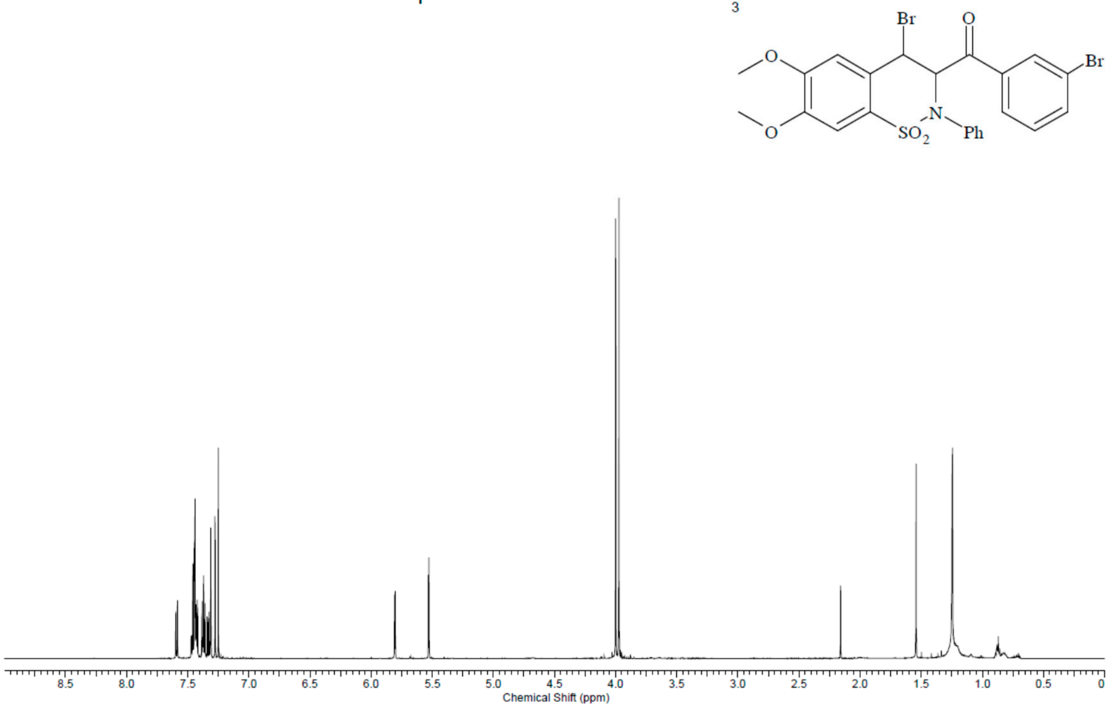

Compound **71** Carbon NMR in $CDCl_3$

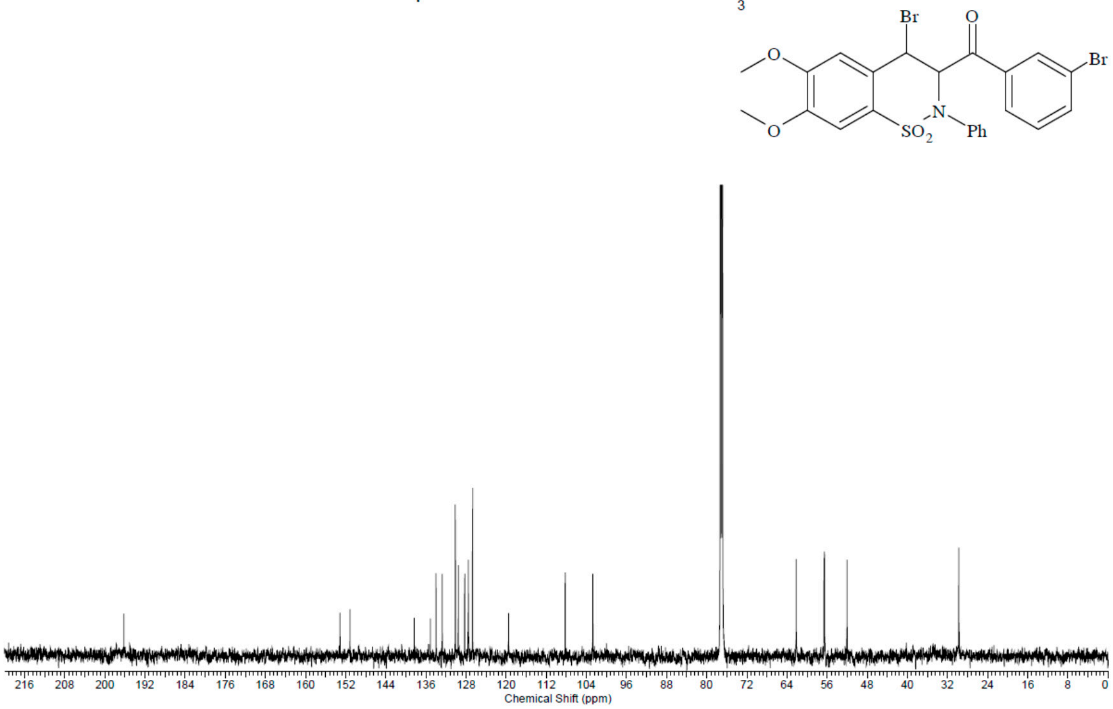

Compound **28** [M + H]+

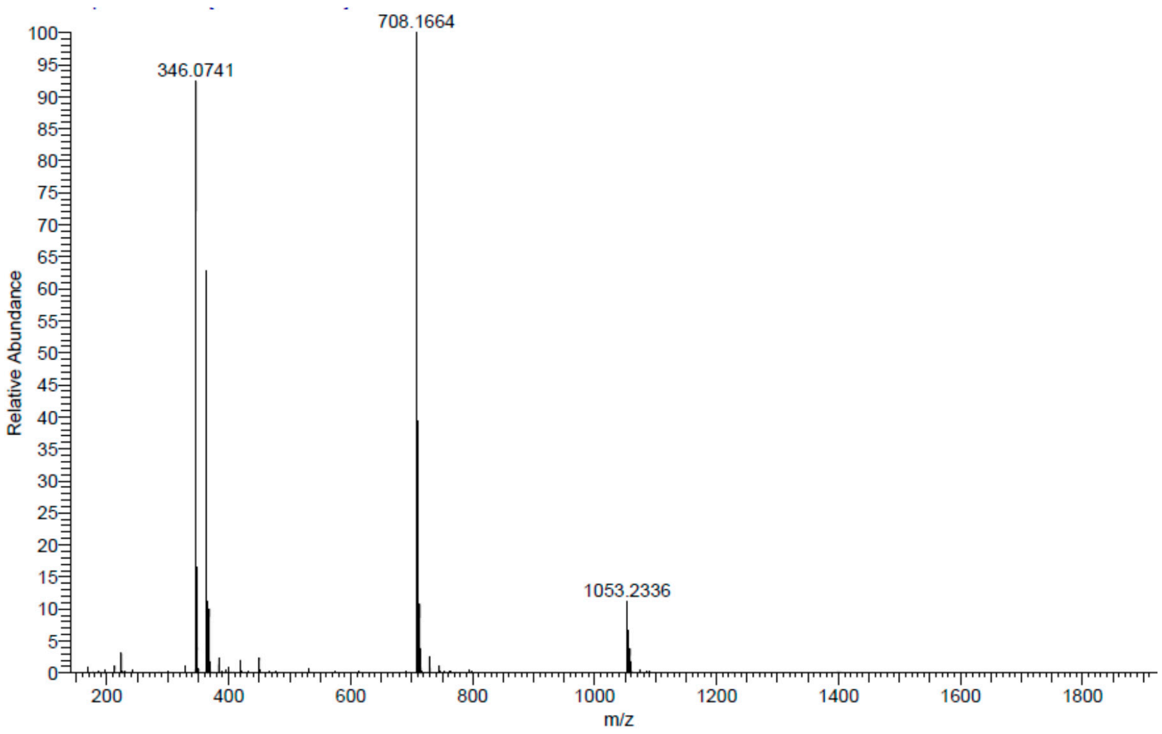

Compound **29** [M + H]+

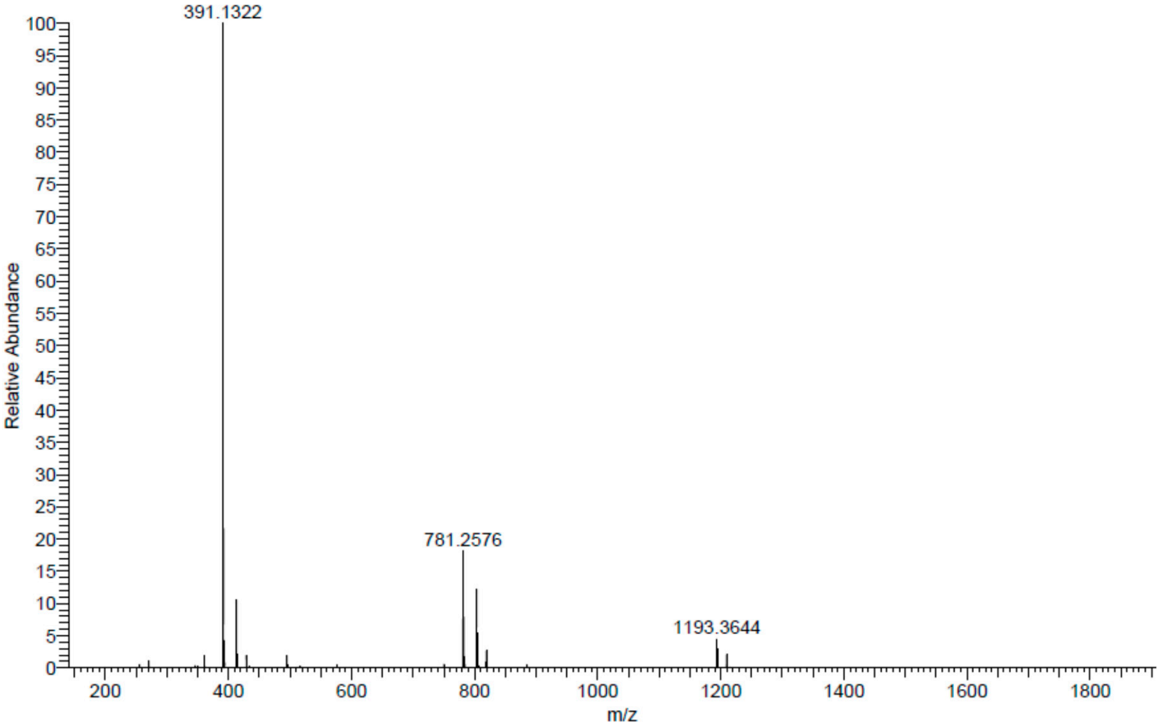

Compound **30** [M + H]+

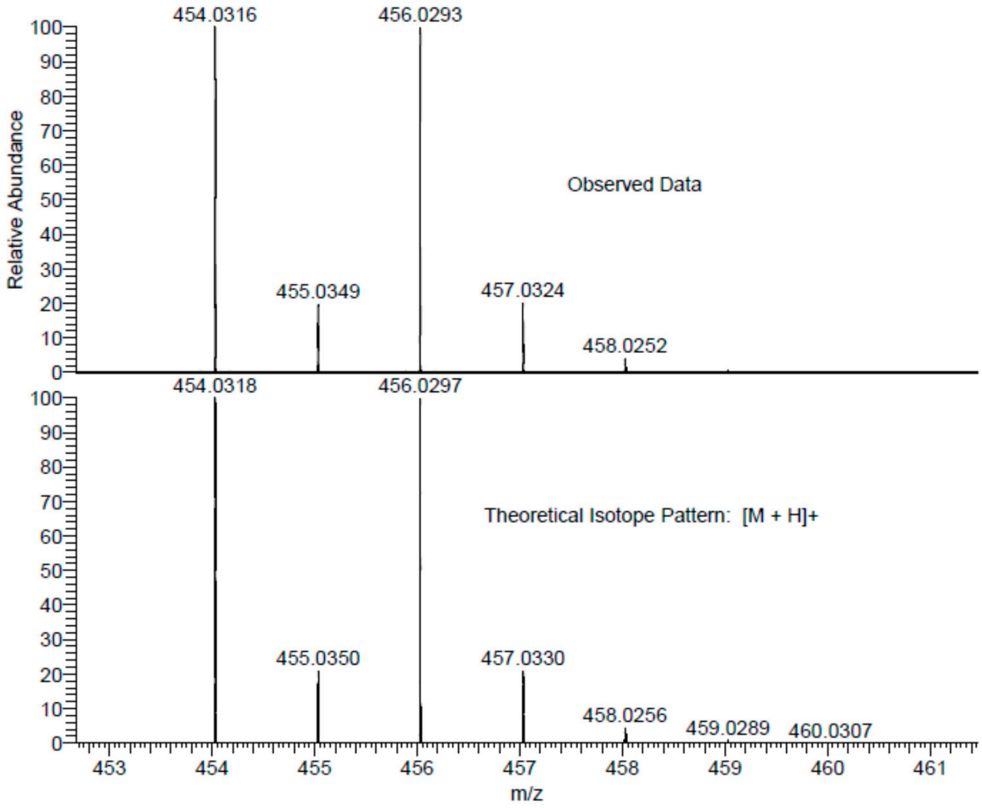

Compound **31** [M + H]+

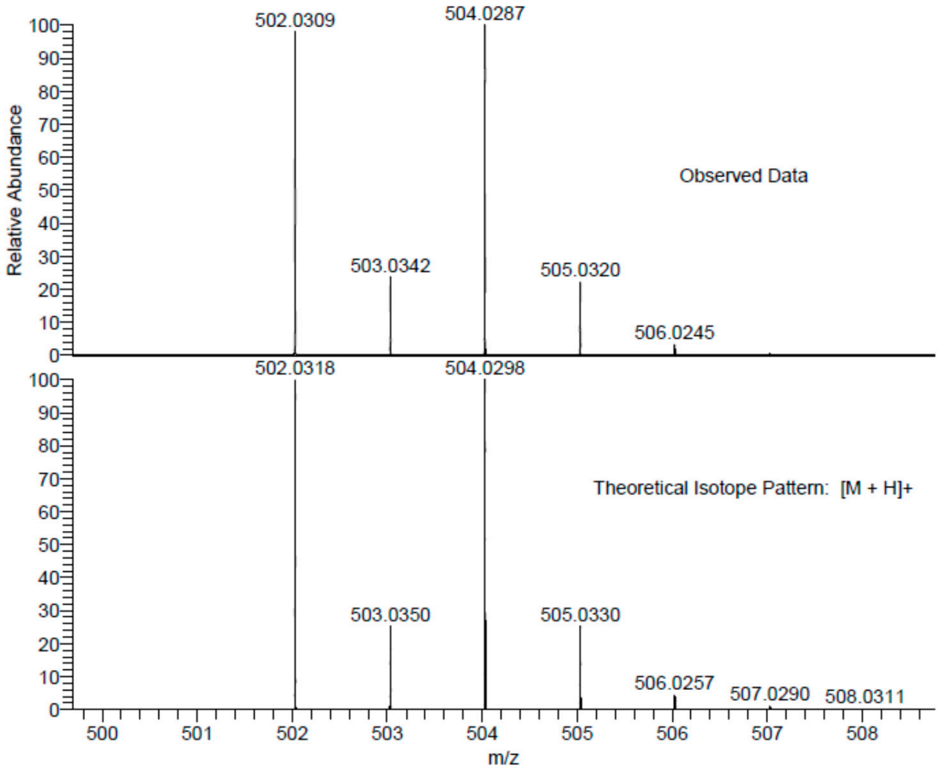

Compound **32** [M + NH_4]$^+$

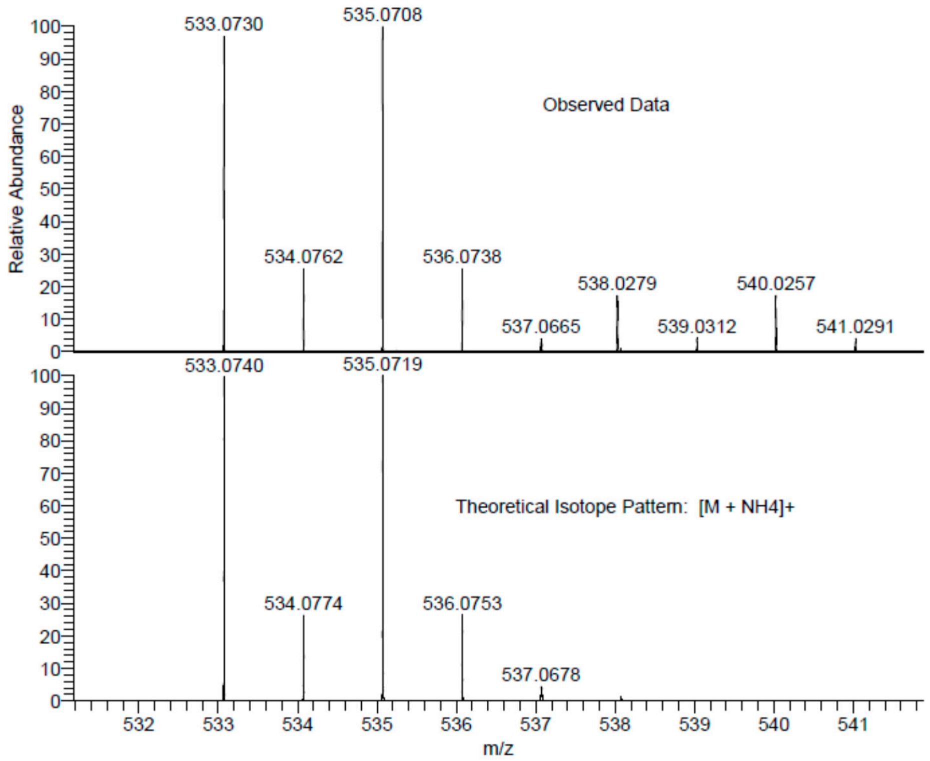

Compound **34** [M + H]$^+$

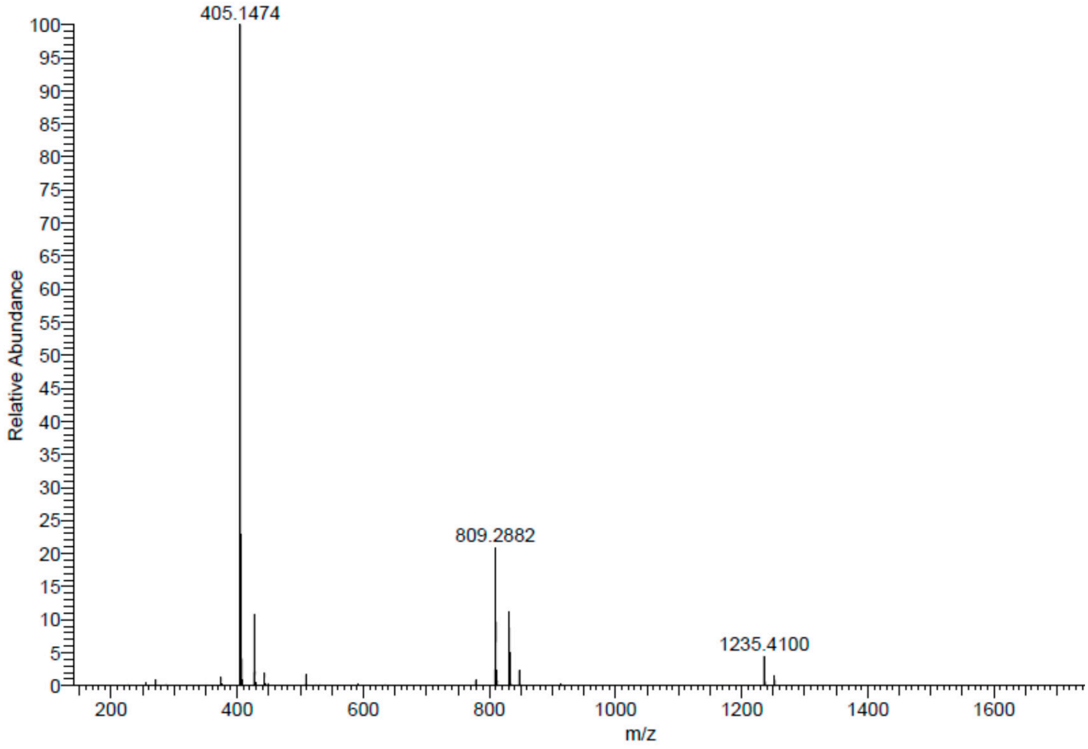

Compound **35** [M + H]+

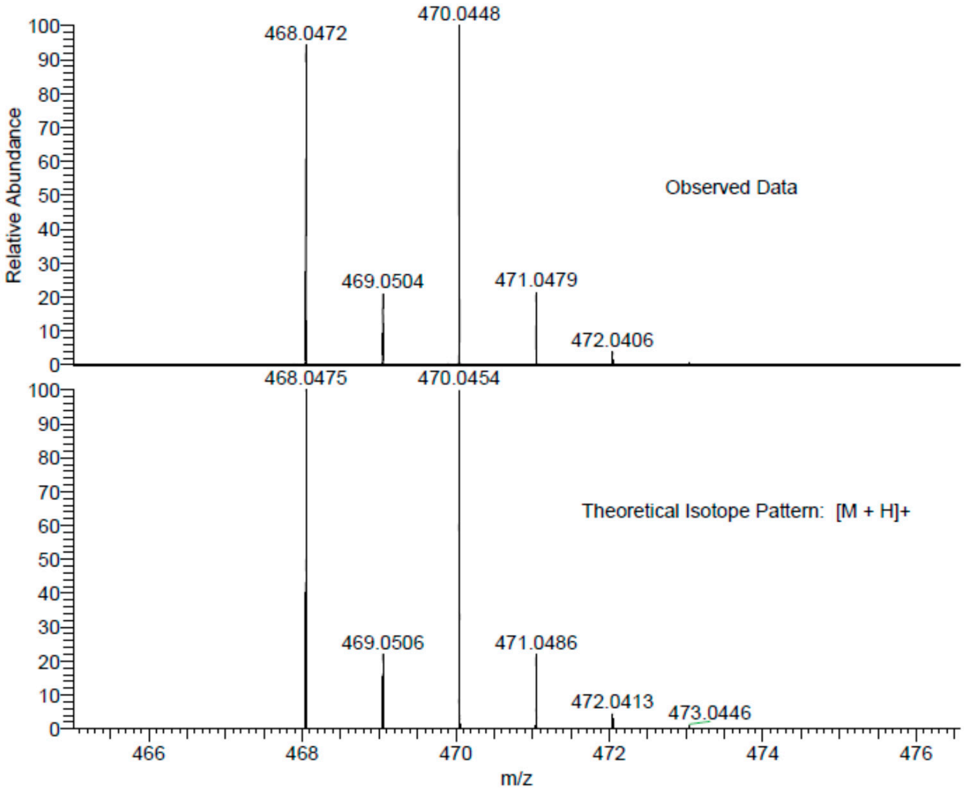

Compound **36** [M + H]+

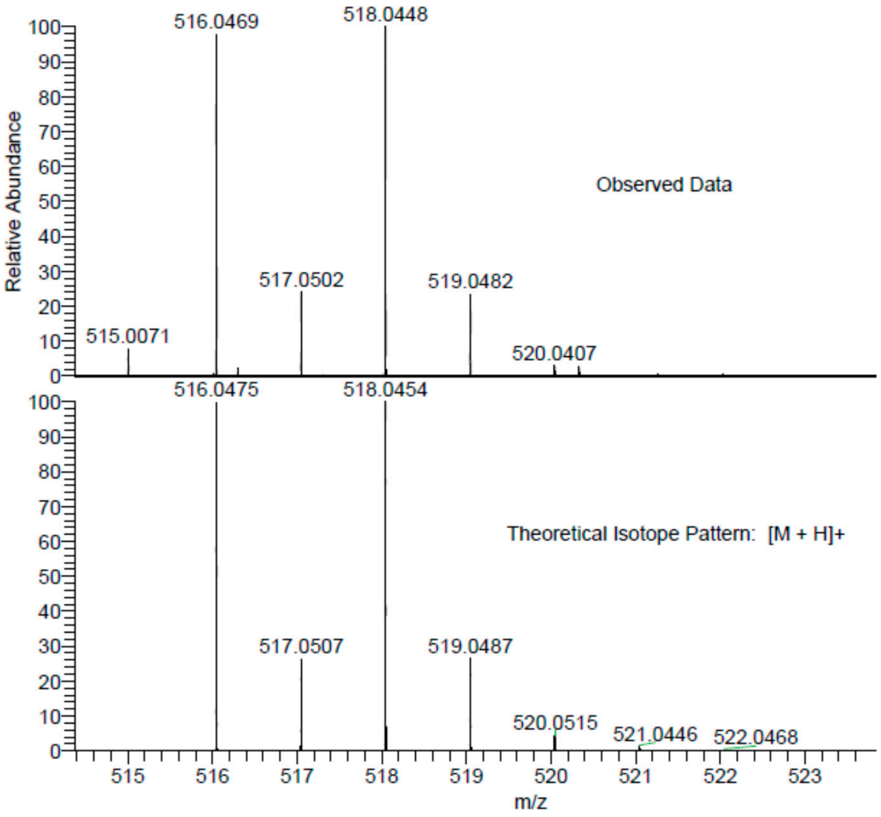

Compound **37** [M + H]⁺

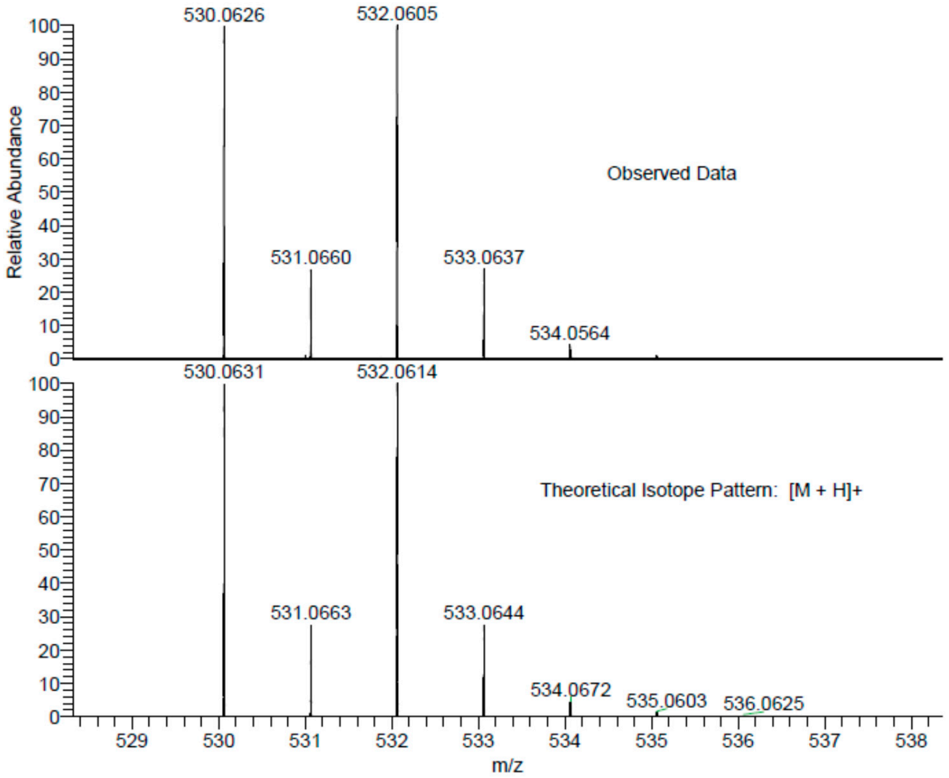

Compound **38** [M − Br]⁺

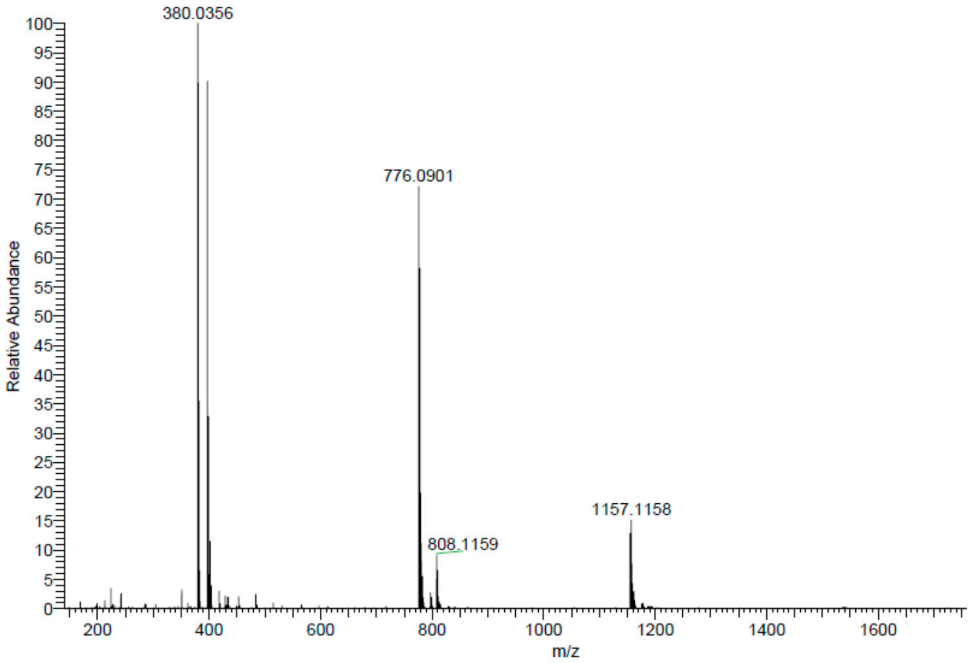

Compound **39** [M + H]+

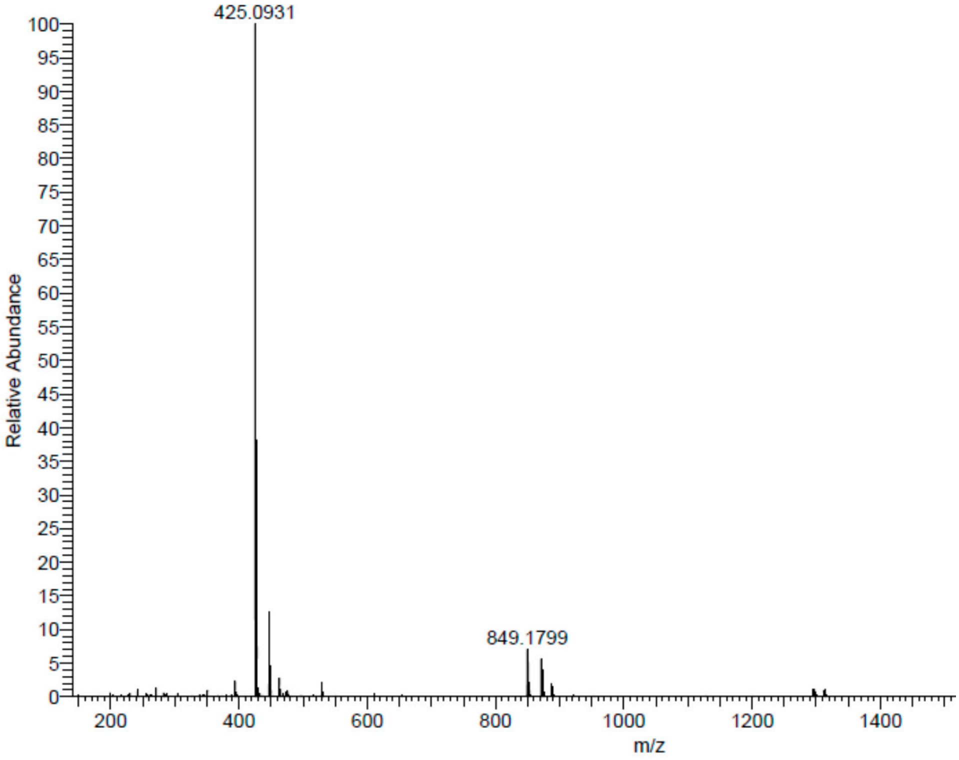

Compound **41** [M + H]+

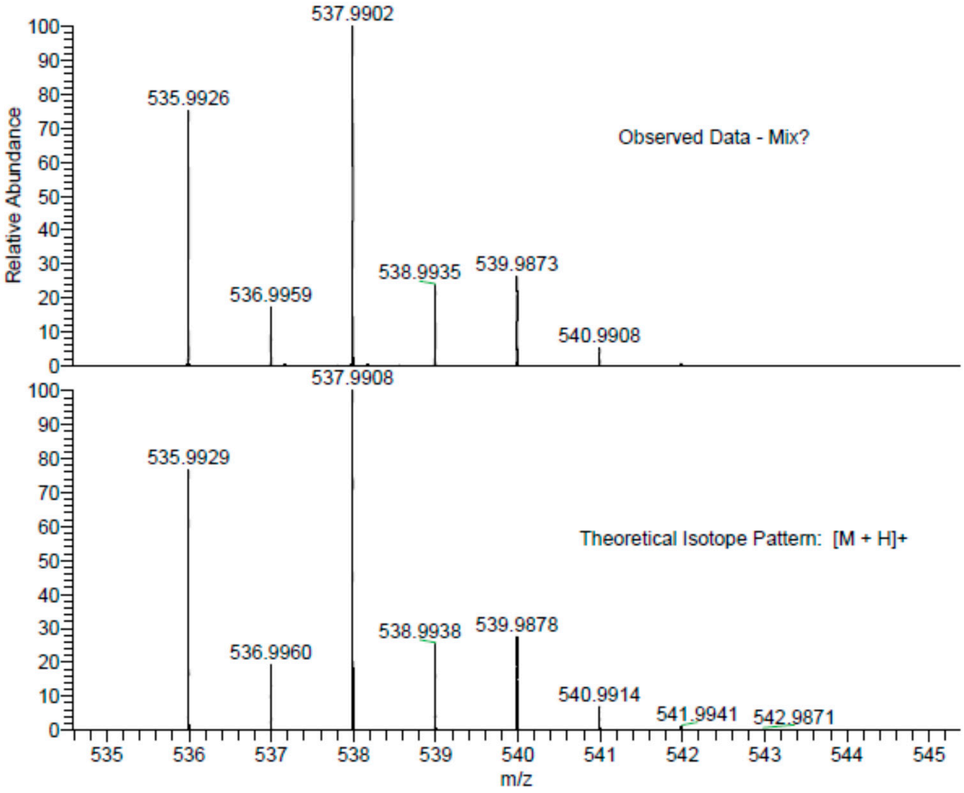

Compound **42** [M + H]+

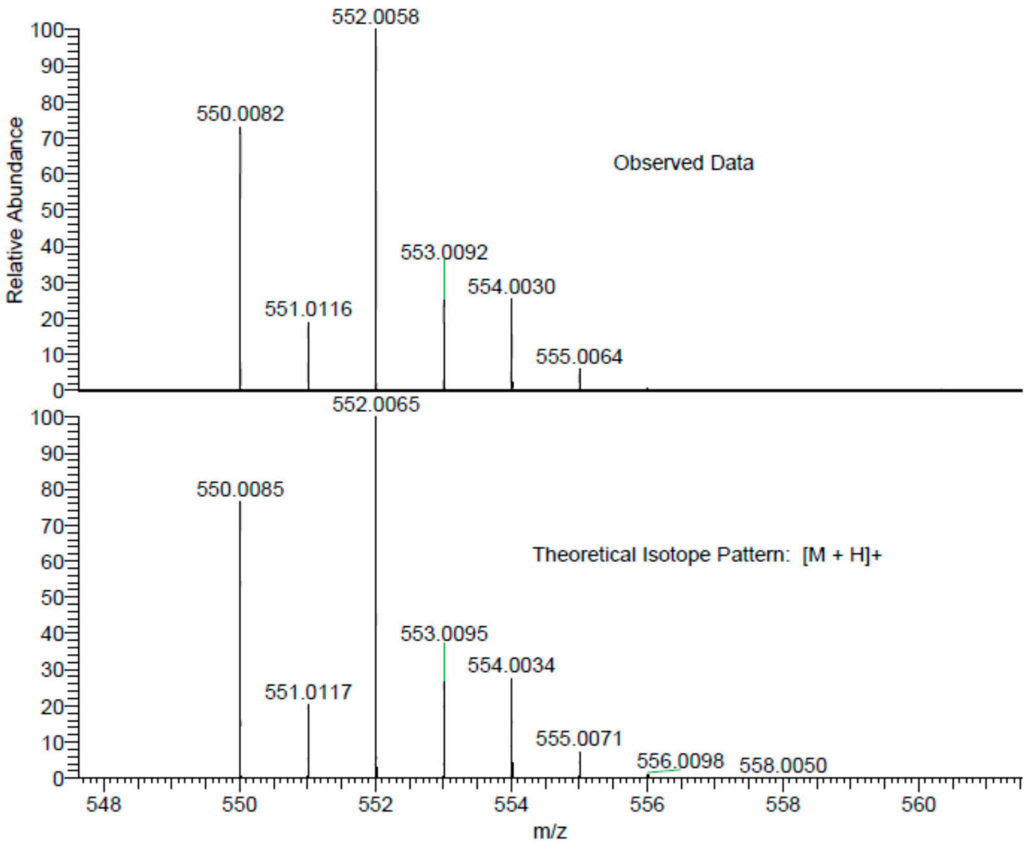

Compound **43** [M − Br]+

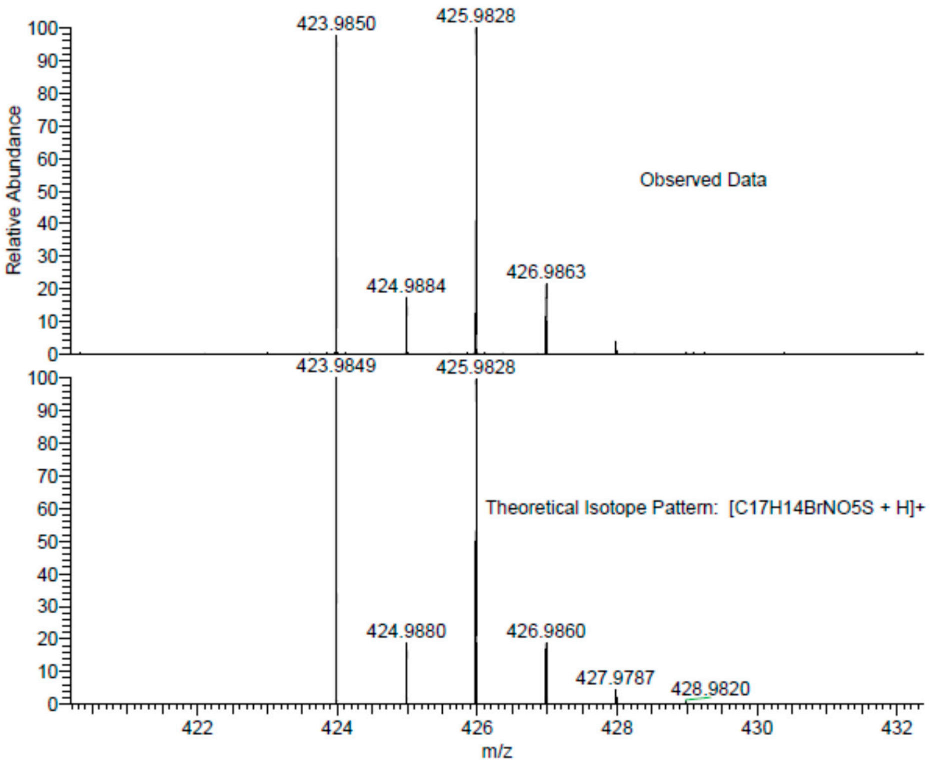

Compound **45** [M + H]$^+$

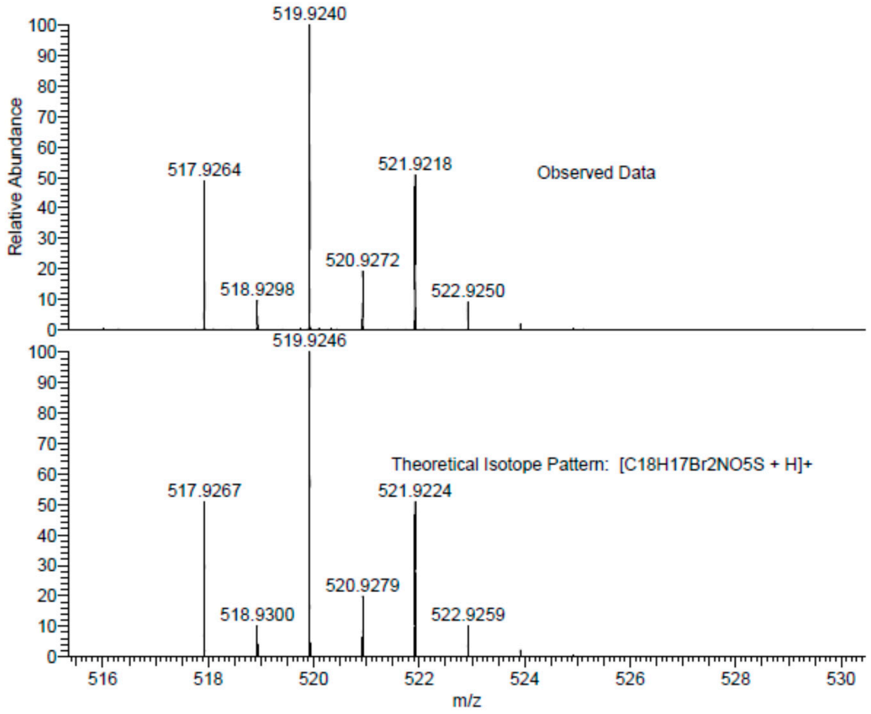

Compound **46** [M + H]$^+$

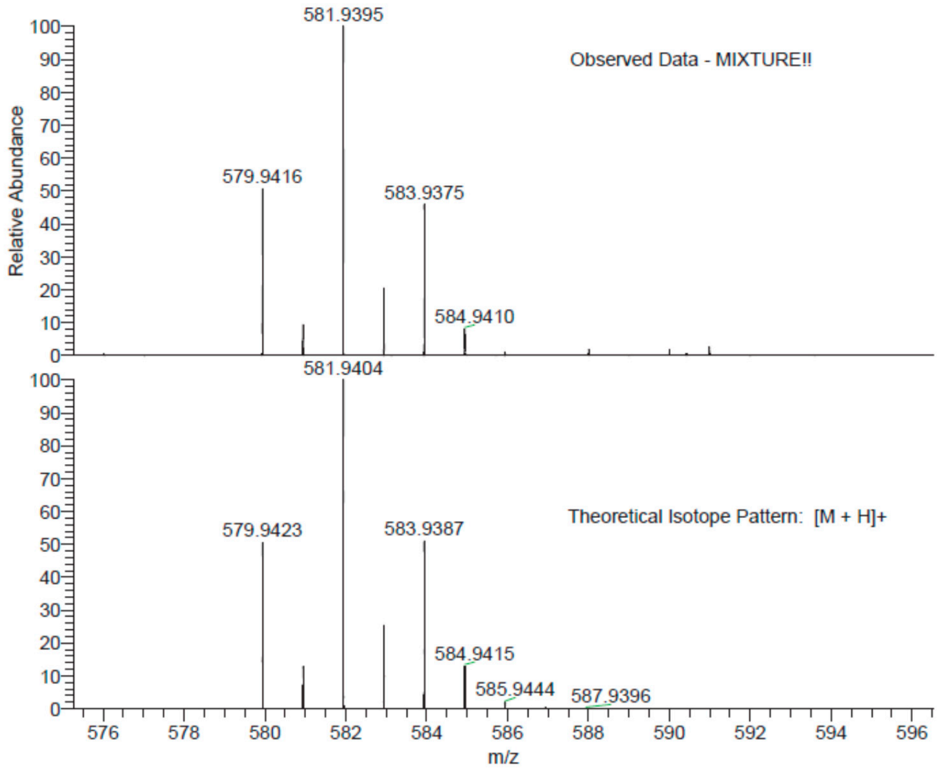

Compound **47** $[M + H]^+$

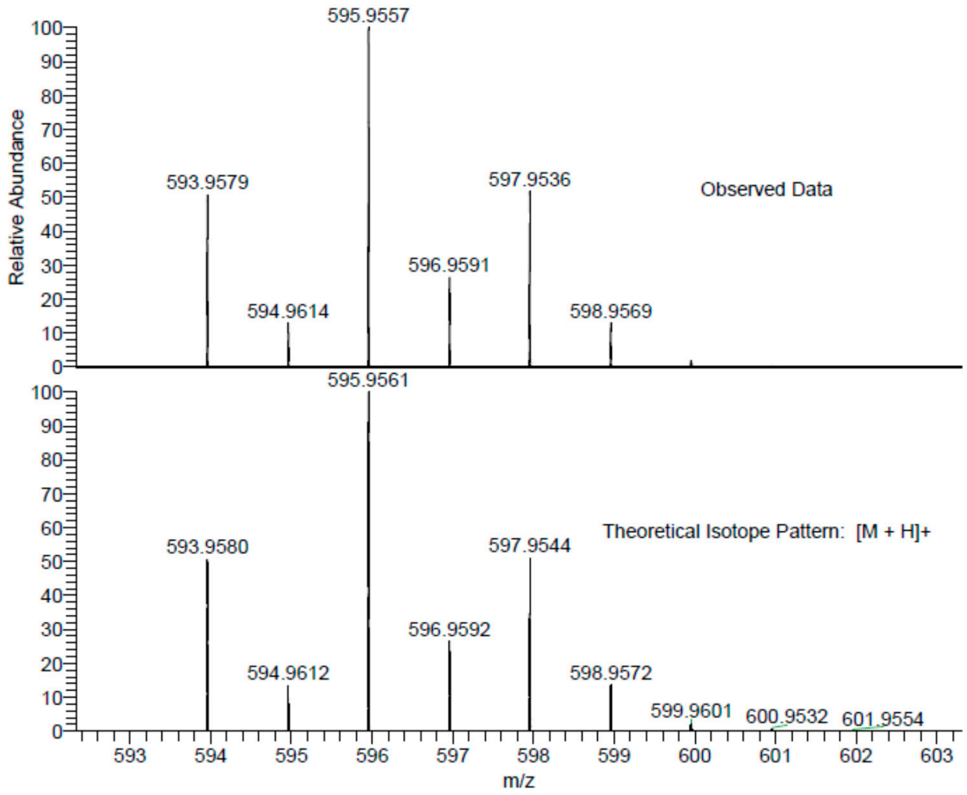

Compound **49** $[M + H]^+$

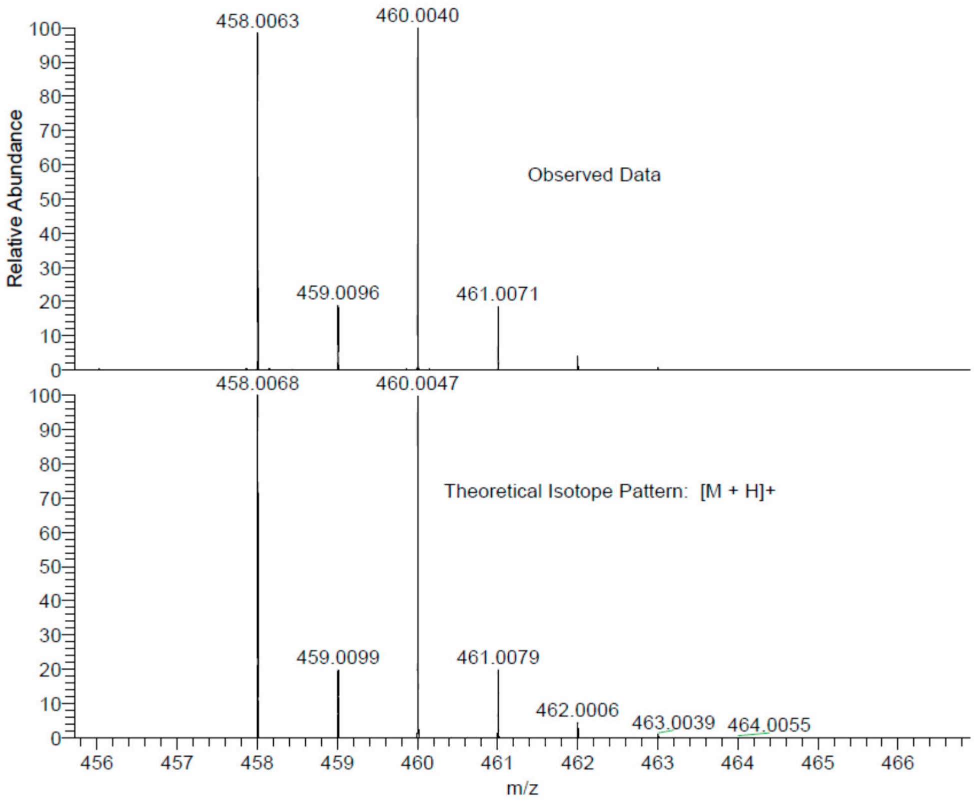

Compound **50** [M + H]+

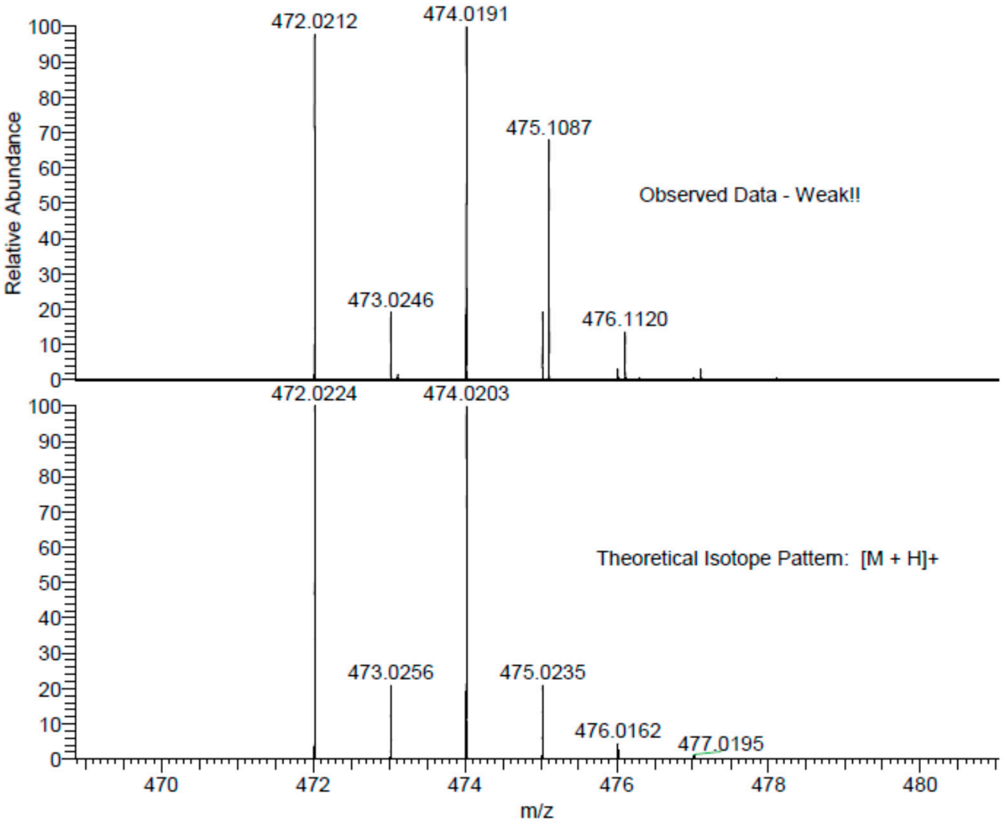

Compound **51** [M + H]+

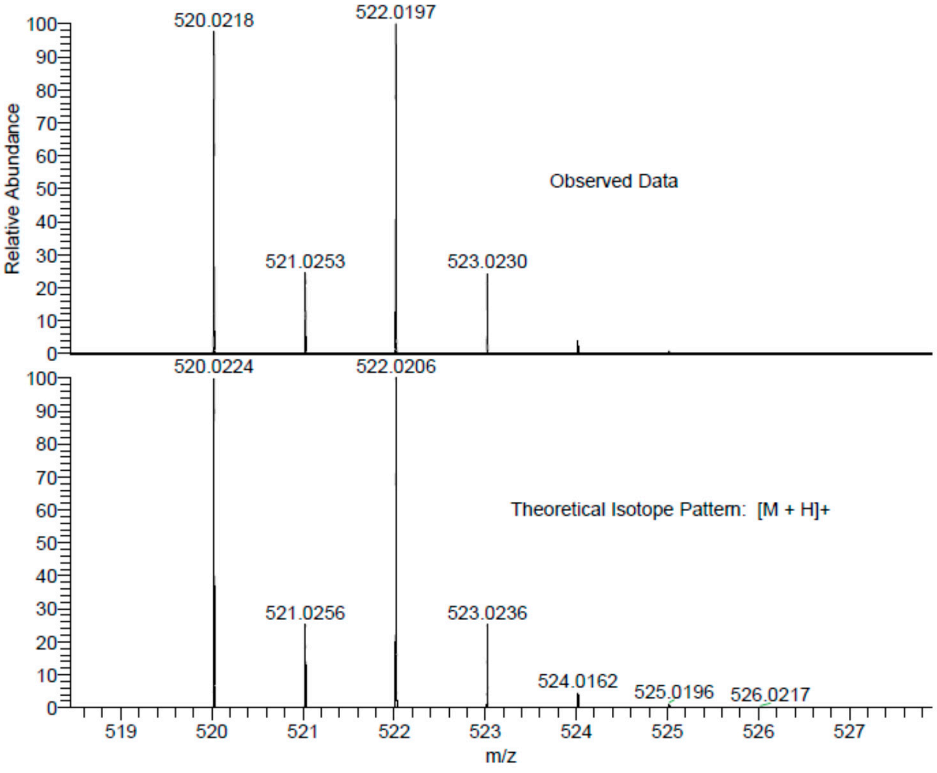

Compound **52** [M + H]+

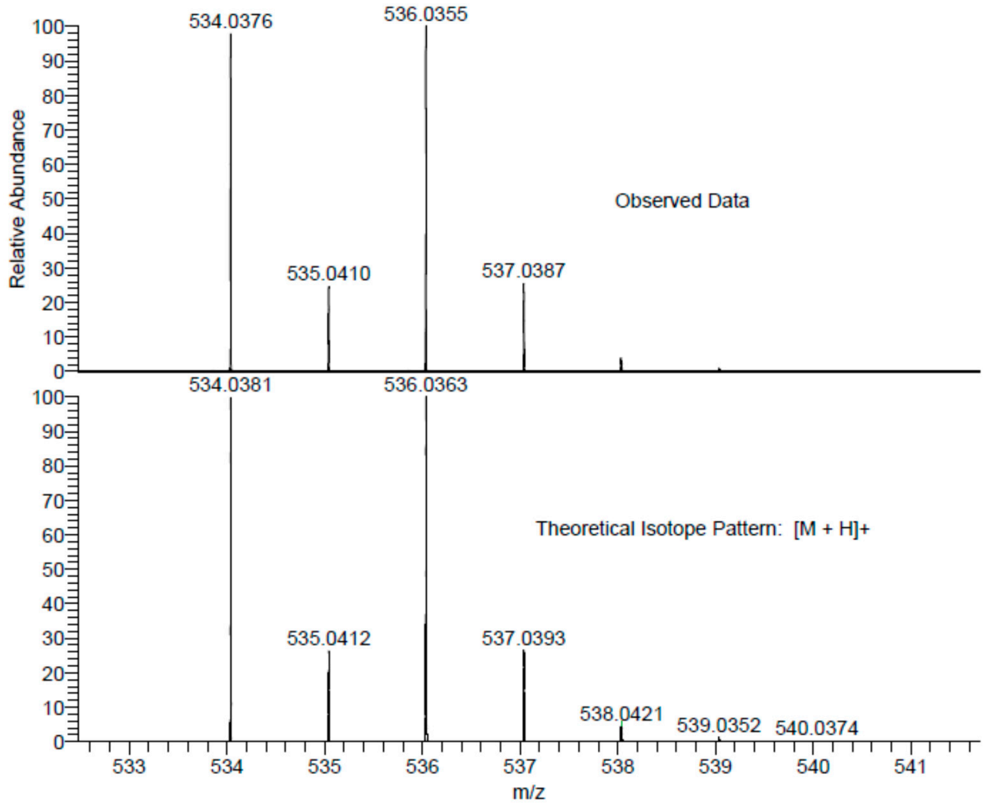

Compound **54** [M + H]+

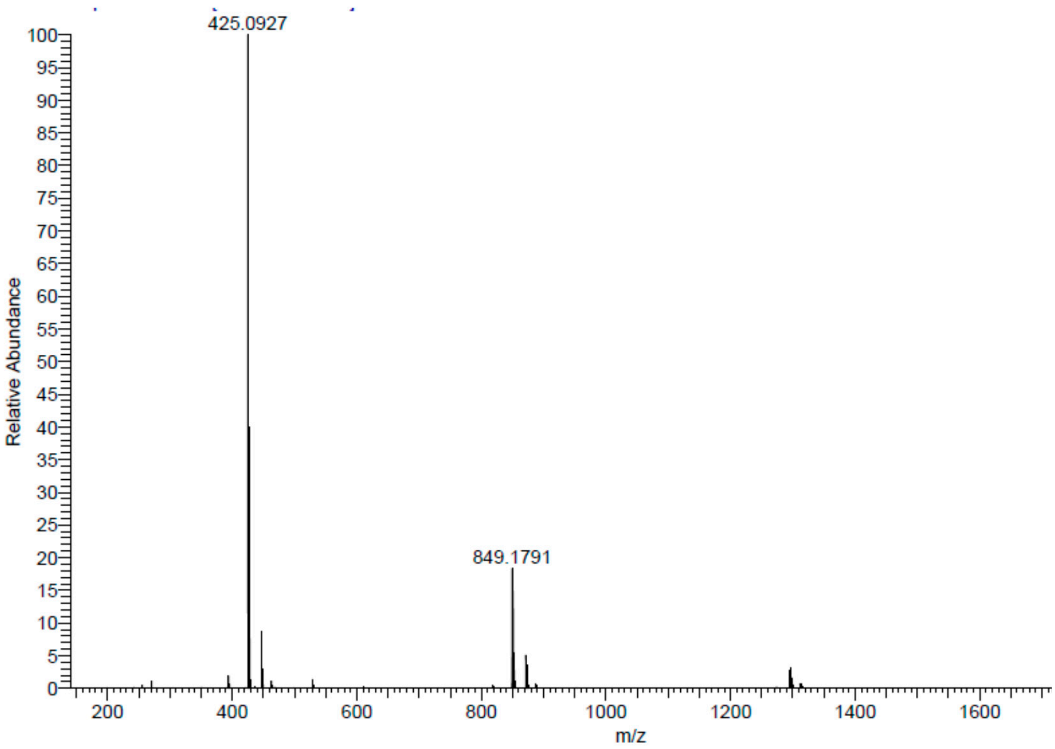

Compound **55** [M + H]+

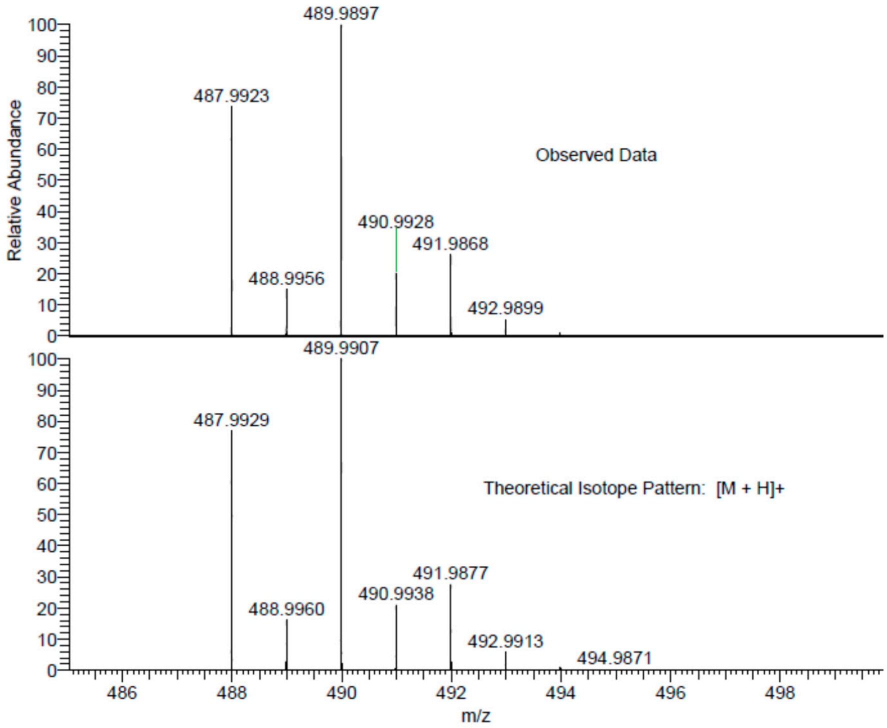

Compound **56** [M + H]+

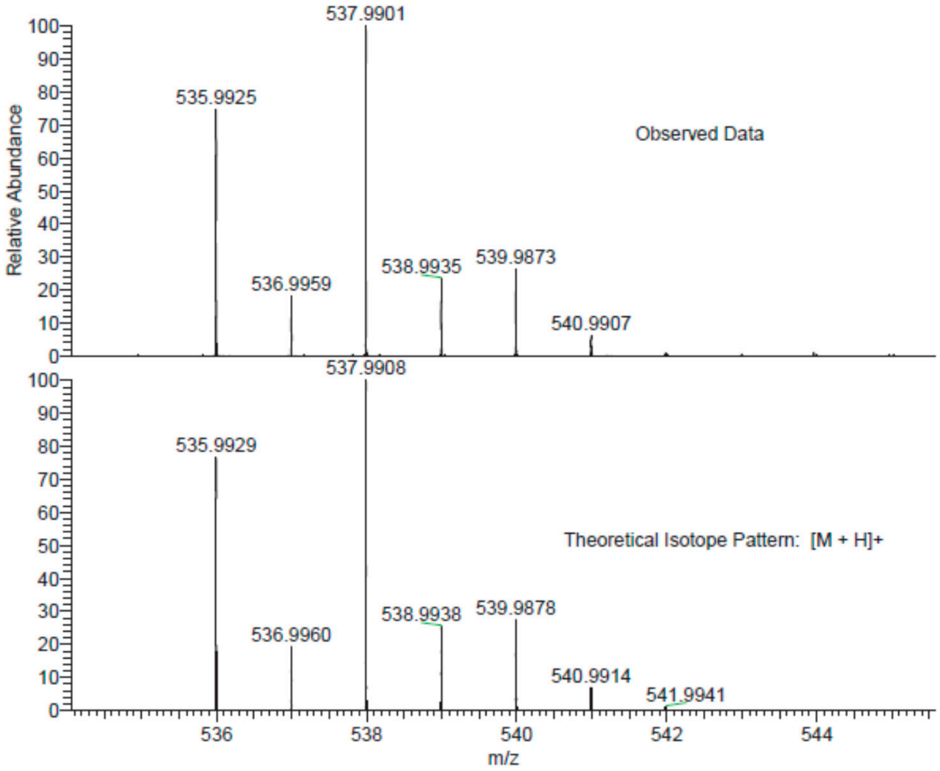

Compound **57** $[M + NH_4]^+$

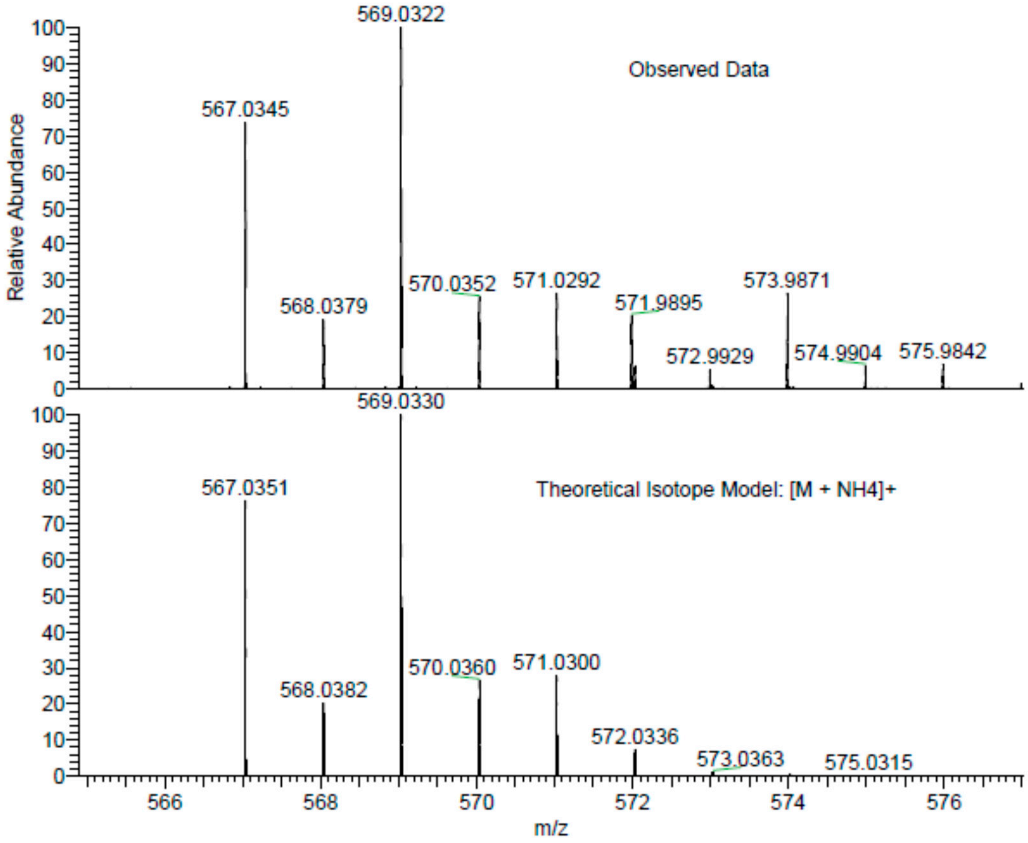

Compound **58** $[M - HBr]^+$

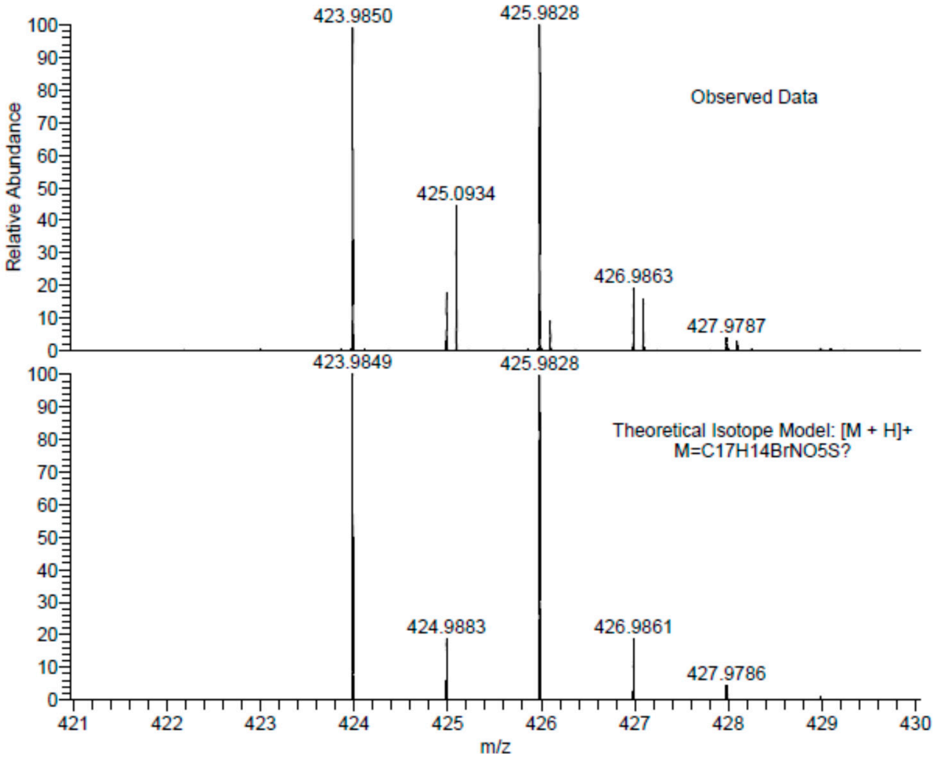

Compound **59** [M + H]+

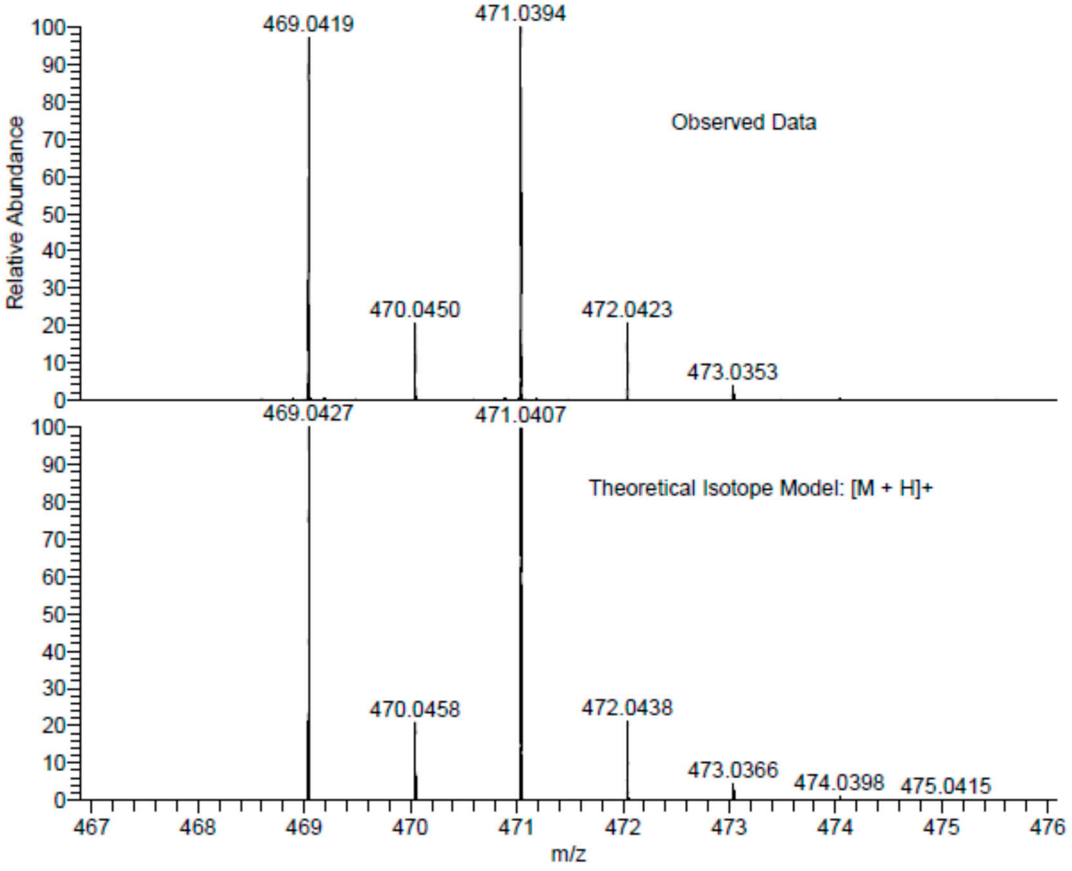

Compound **60** [M + H]+

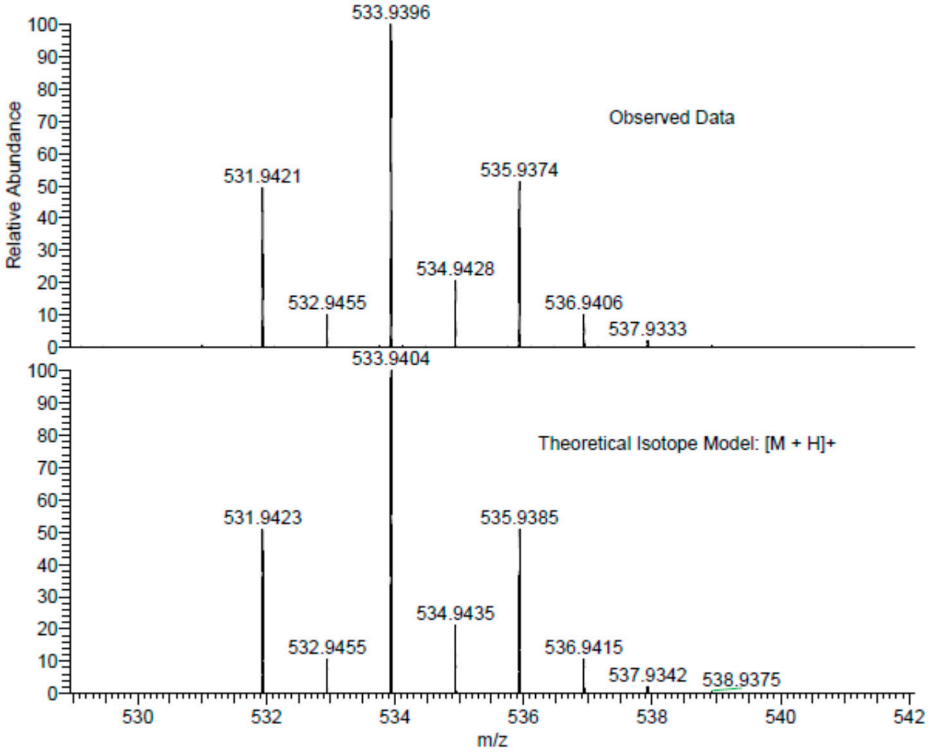

Compound **61** [M + NH_4]$^+$

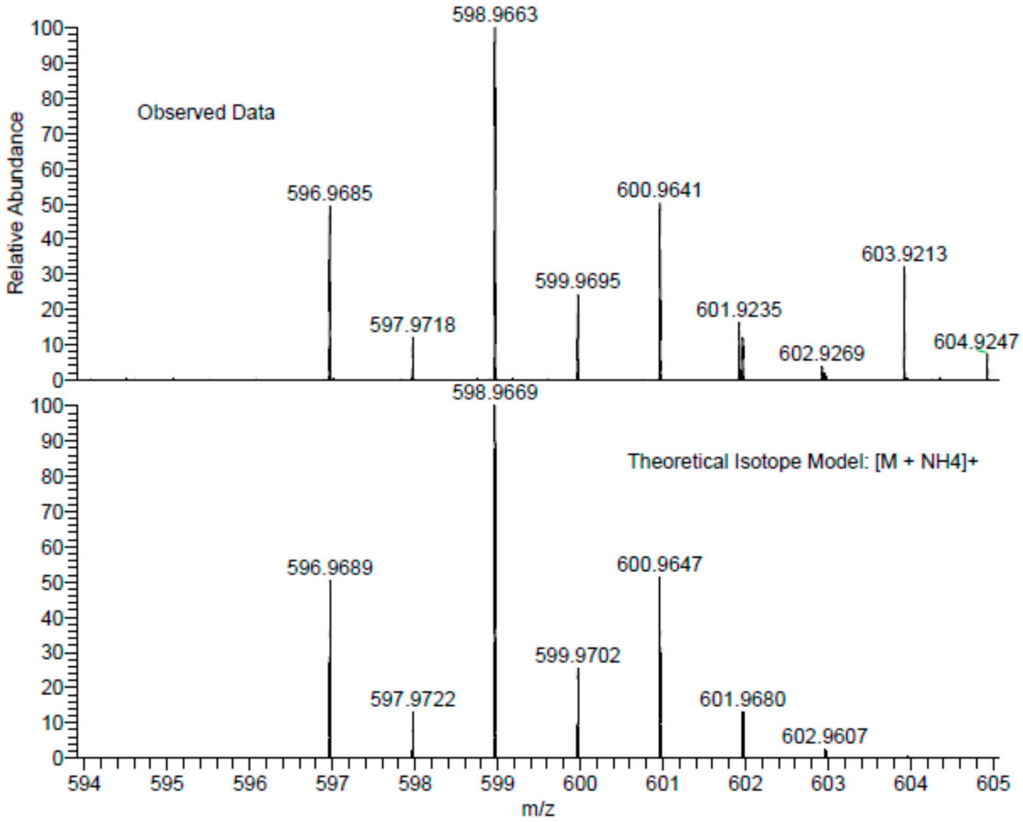

Compound **62** [M + NH_4]$^+$

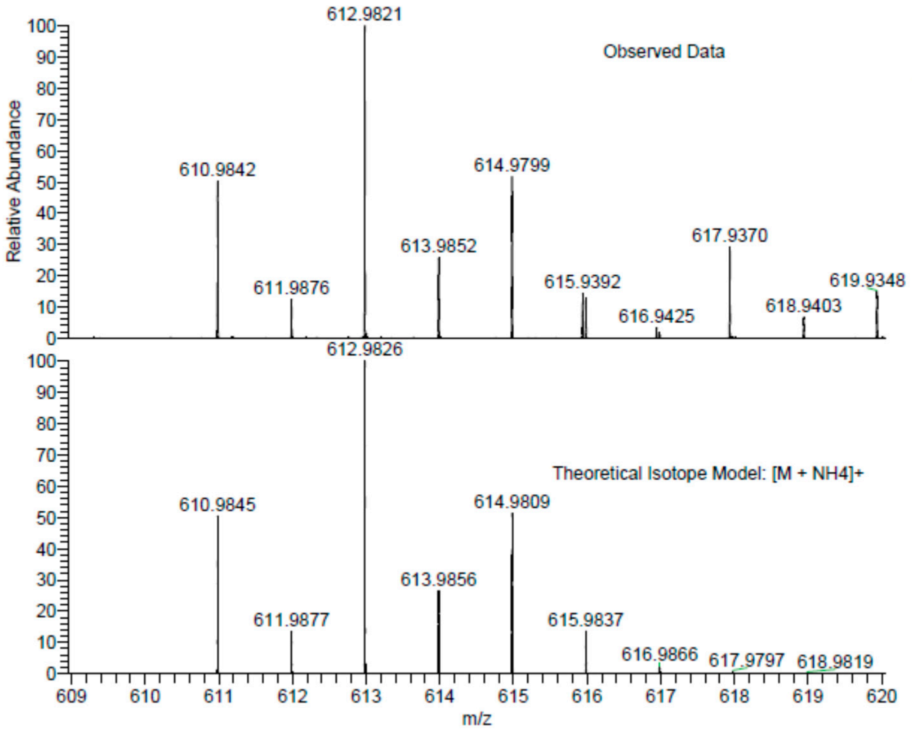

Compound **64** [M + H]$^+$

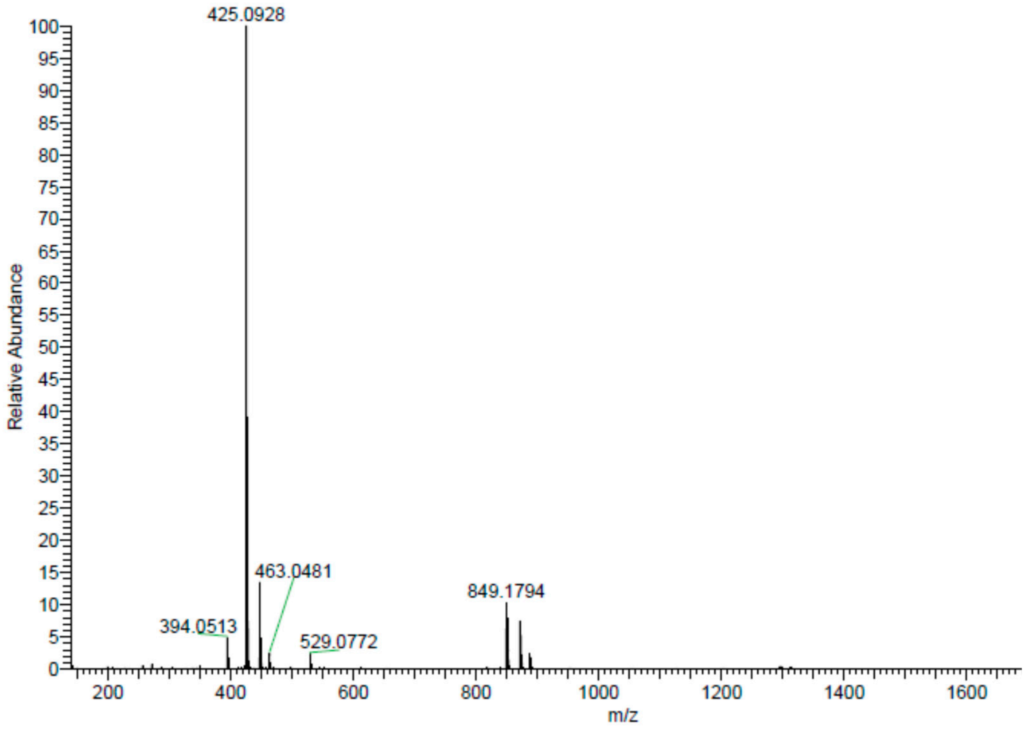

Compound **65** [M + H]$^+$

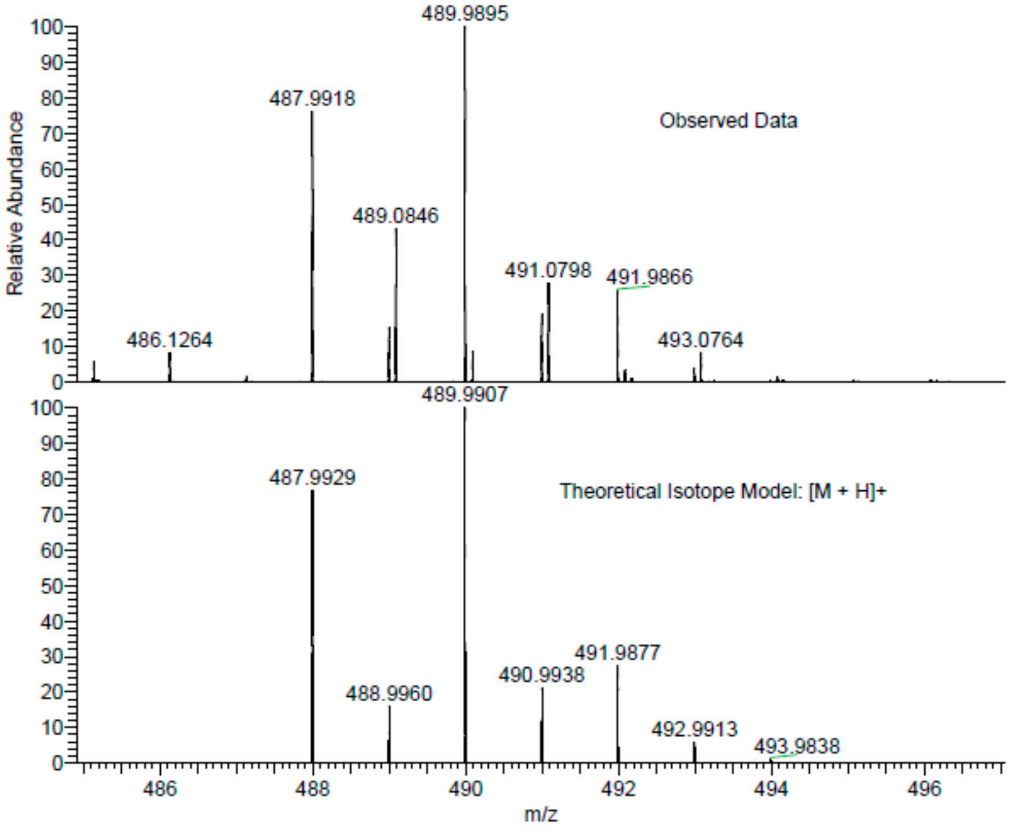

Compound **66** $[M + H]^+$

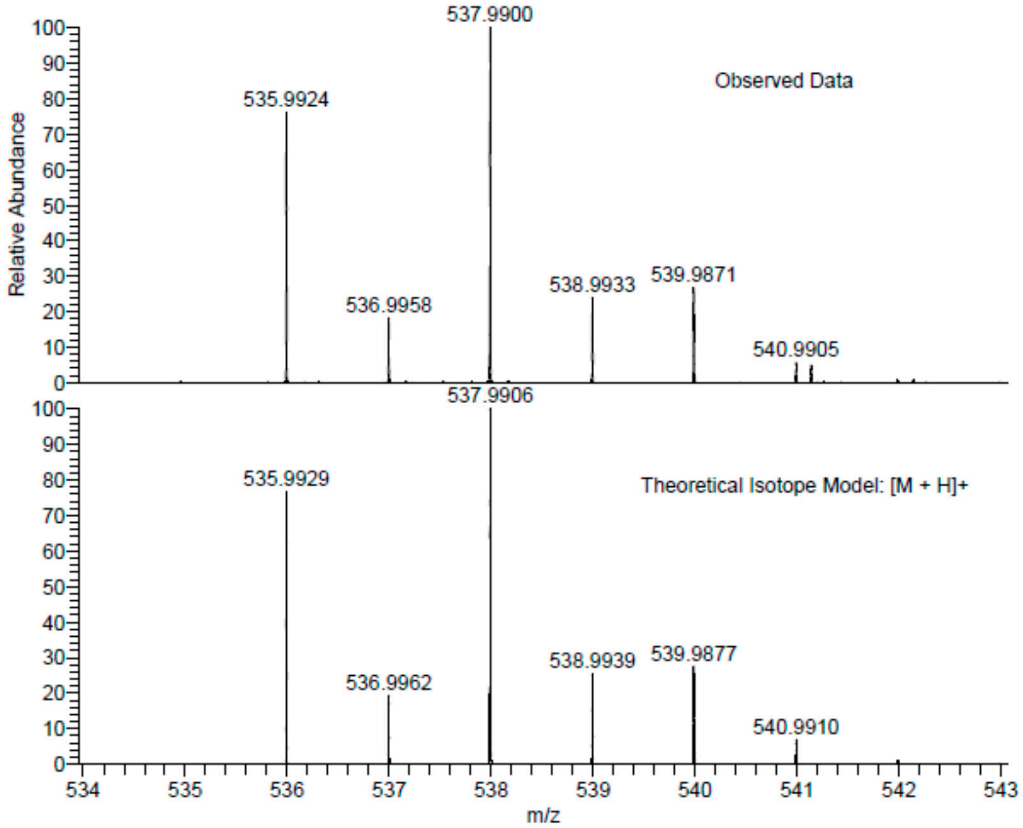

Compound **67** $[M + NH_4]^+$

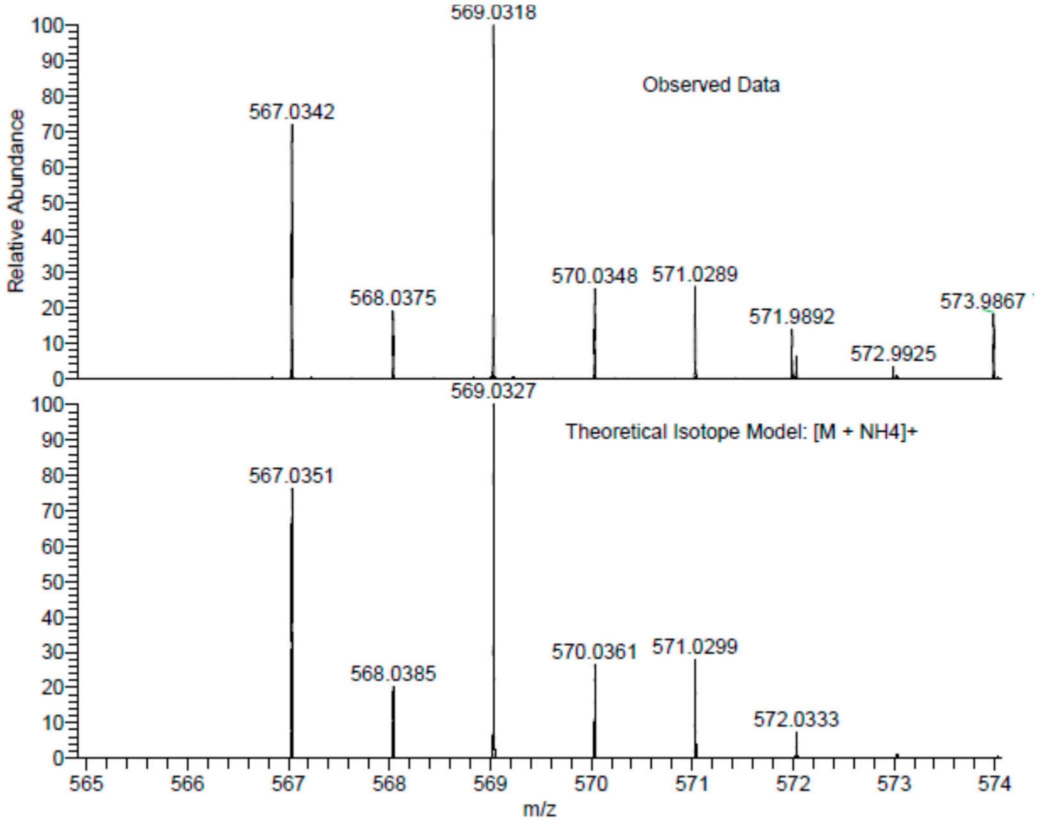

Compound **69** [M + H]+

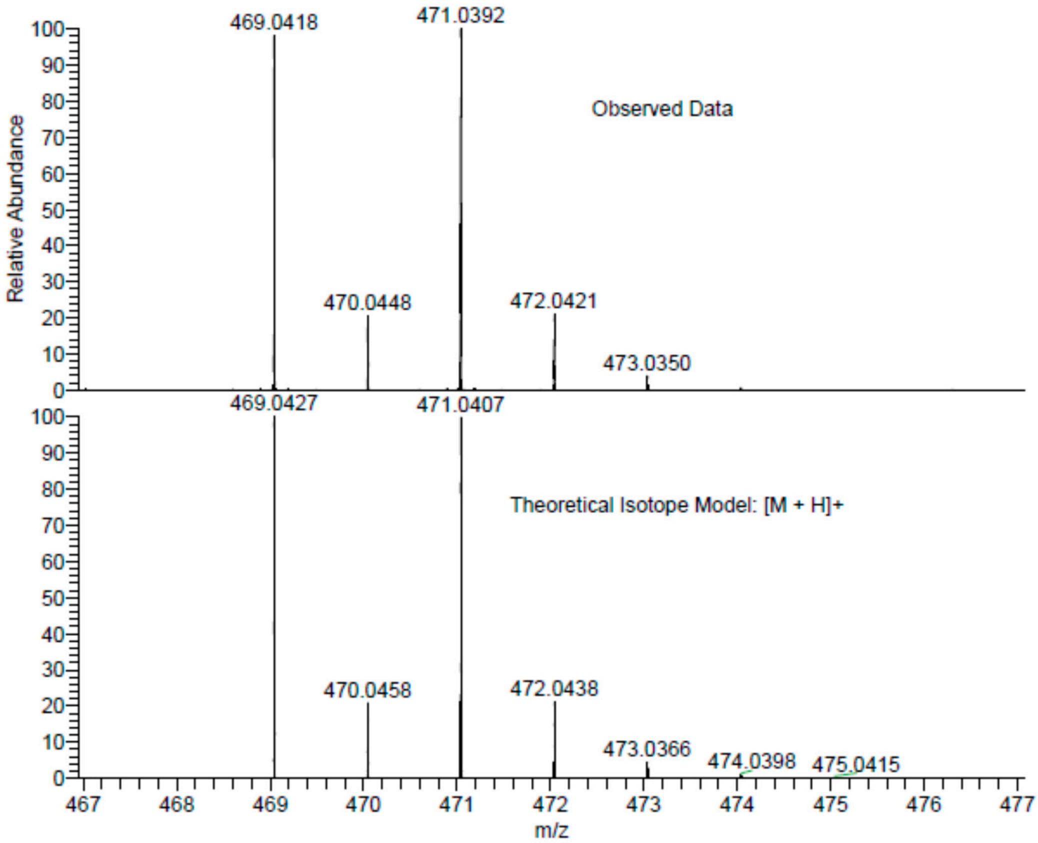

Compound **71** [M + H]+

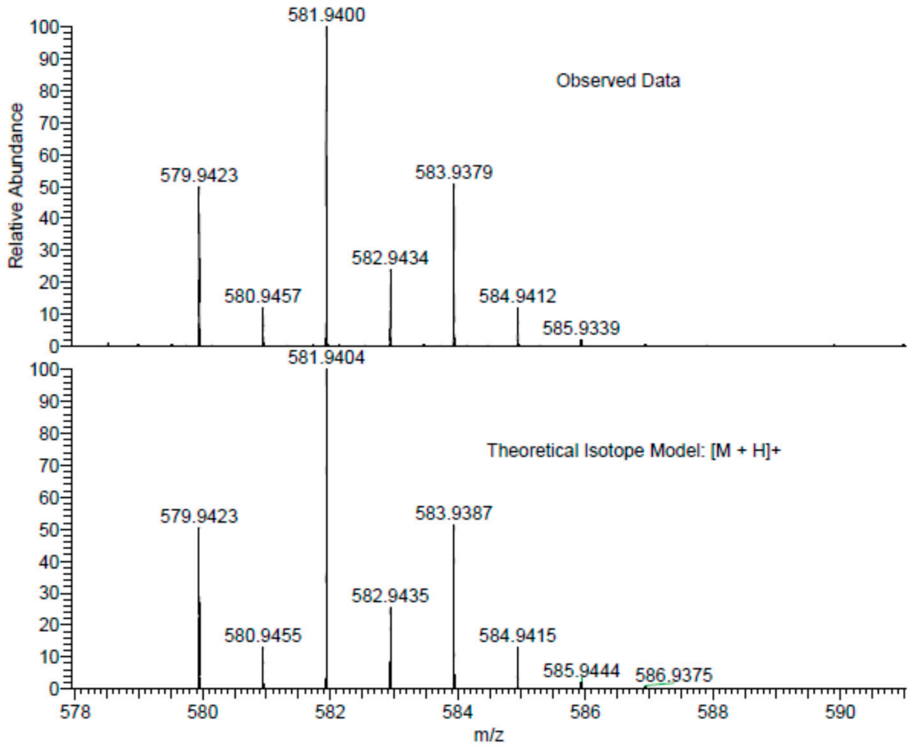

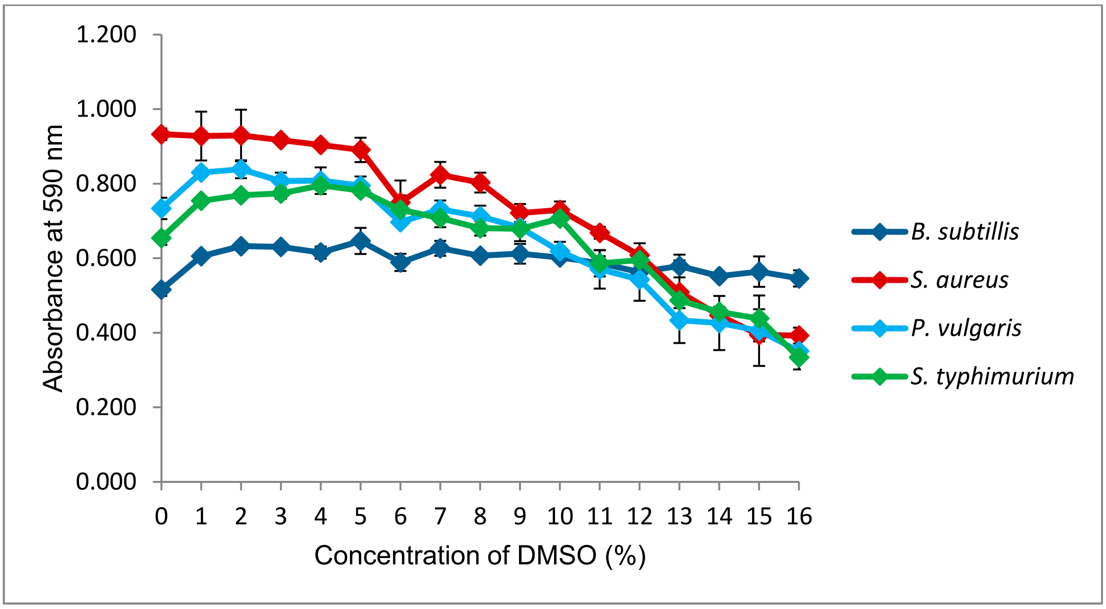

Figure S1. The absorbance measured at the 590 nm wave-length for *Bacillus subtilis*, *Staphylococcous aureus*, *Proteus vulgaris* and *Salmonella typhimurium* with different percentages of DMSO ranging from 0% to 16%. The experiment was conducted in triplicate. There is no decrease in absorbance up to 5% DMSO, meaning there is no inhibition of growth.

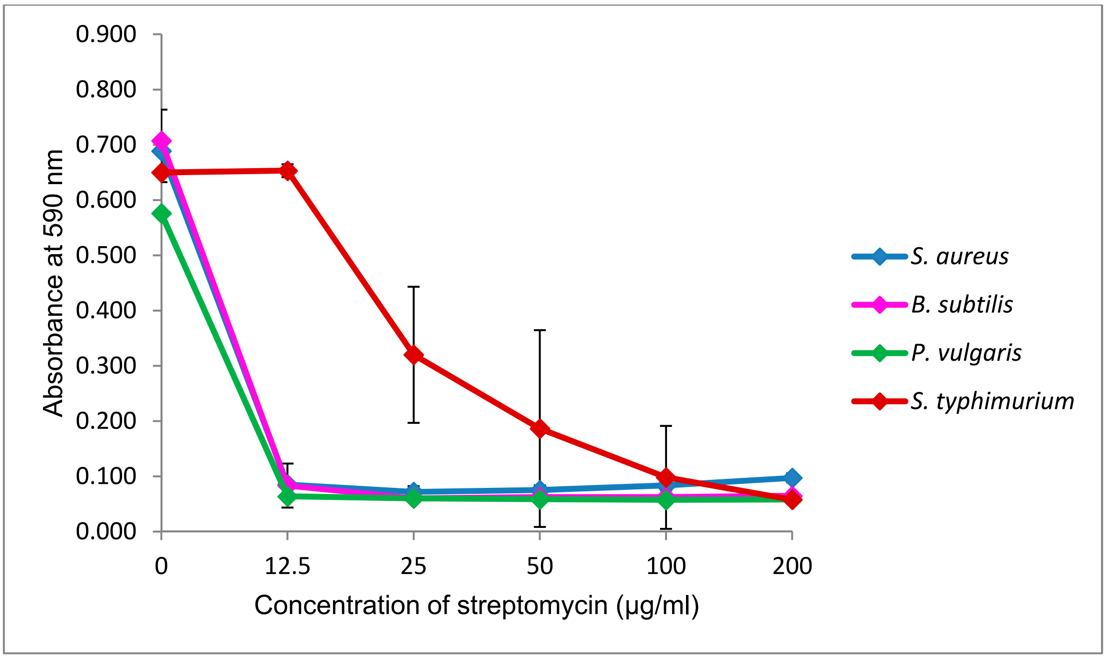

Figure S2. The graph shows the absorbance measured at the 590 nm wave-length after 16 h of incubation for *Bacillus subtilis, Staphylococcous aureus, Proteus vulgaris* and *Salmonella typhimurium* with different concentrations of streptomycin ranging from 12.5 µg/mL to 600 µg/mL. The experiment was conducted in triplicate.

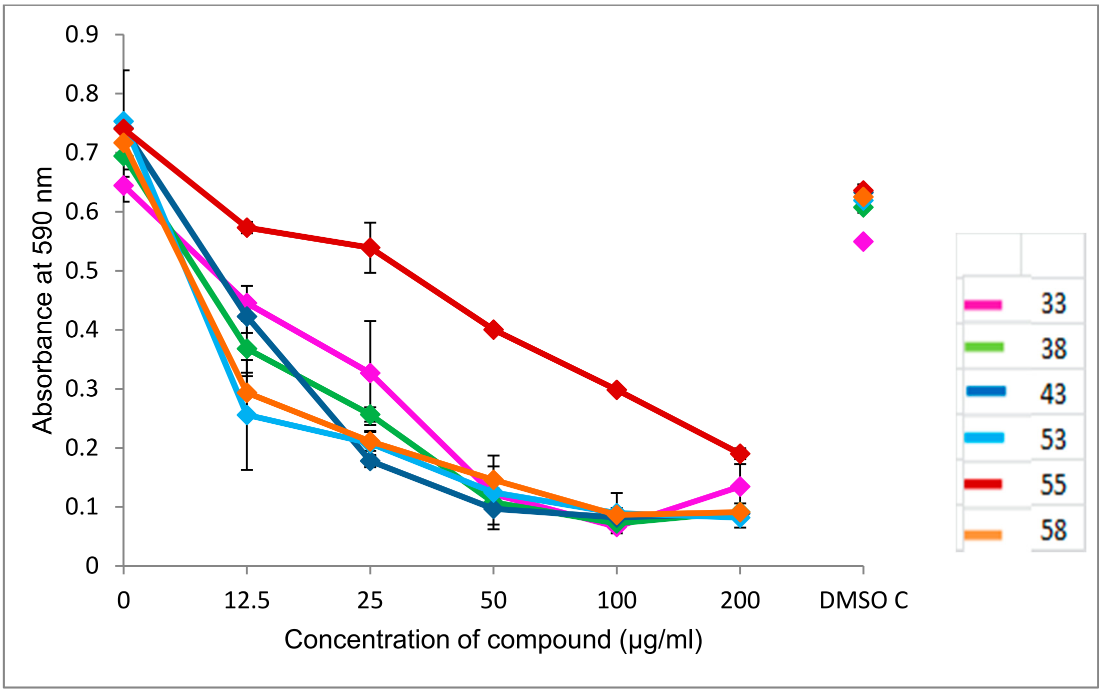

Figure S3. The graph shows the absorbance measured at the 590 nm wave-length after 16 h of incubation for *Staphylococcus aureus* with different concentrations of compounds **33**, **38**, **43**, **53**, **55** and **58** ranging from 12.5 µg/mL to 200 µg/mL. As there is decrease in absorbance meaning the growth of the bacteria is inhibited. At the end of the graph the results of DMSO plus compounds without bacteria are given as a control.

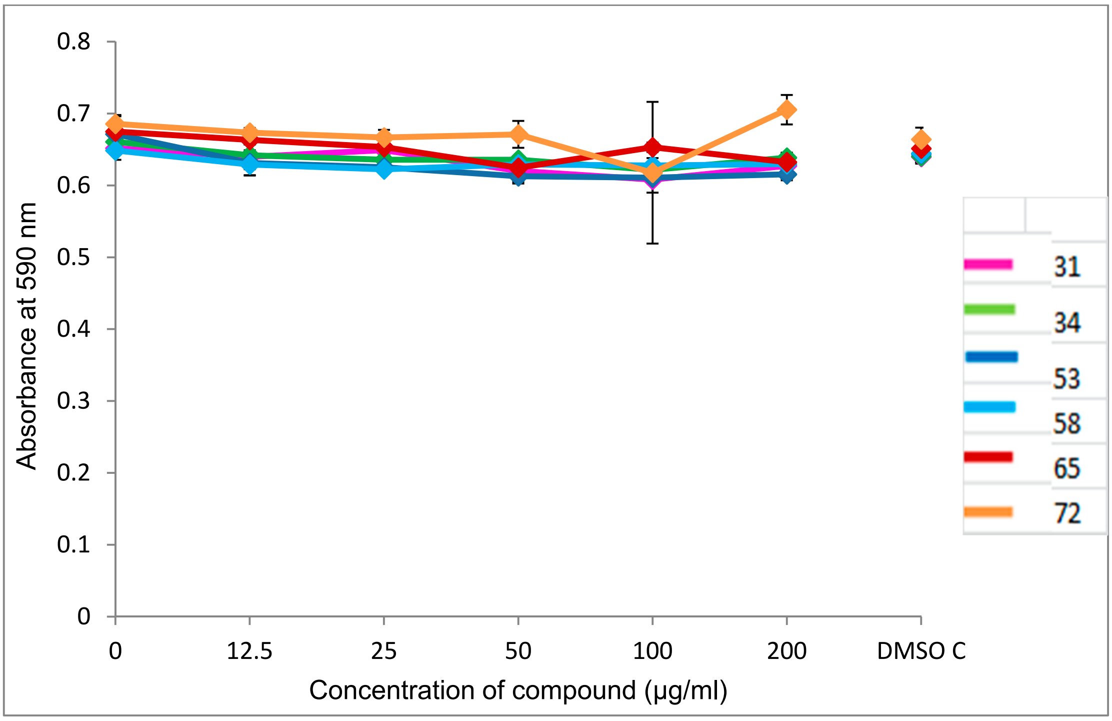

Figure S4. The graph shows the absorbance measured at the 590 nm wave-length after 16 h of incubation for *Salmonella typhimurium* with different concentrations of compounds **31**, **34**, **53**, **58**, **65** and **72** ranging from 12.5 µg/mL to 600 µg/mL. There is no decrease in absorbance for any of the compounds, meaning there is no inhibition of growth. At the end is the DMSO control.

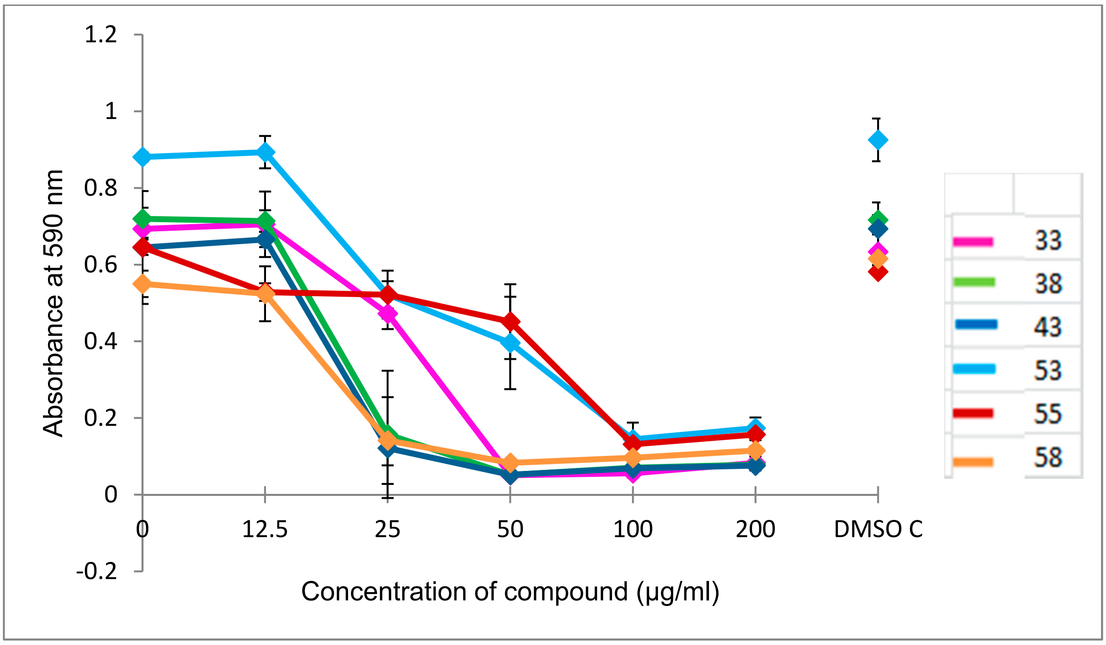

Figure S5. The graph shows the absorbance measured at the 590 nm wave-length after 16 h of incubation for *Bacillus subtilis* with different concentrations of compounds **33**, **38**, **43**, **53**, **55**, and **58** ranging from 12.5 µg/mL to 200 µg/mL. As there is decrease in absorbance meaning, the growth of bacteria is inhibited. At the end of the graph the results of DMSO plus compounds without bacteria are given as a control.

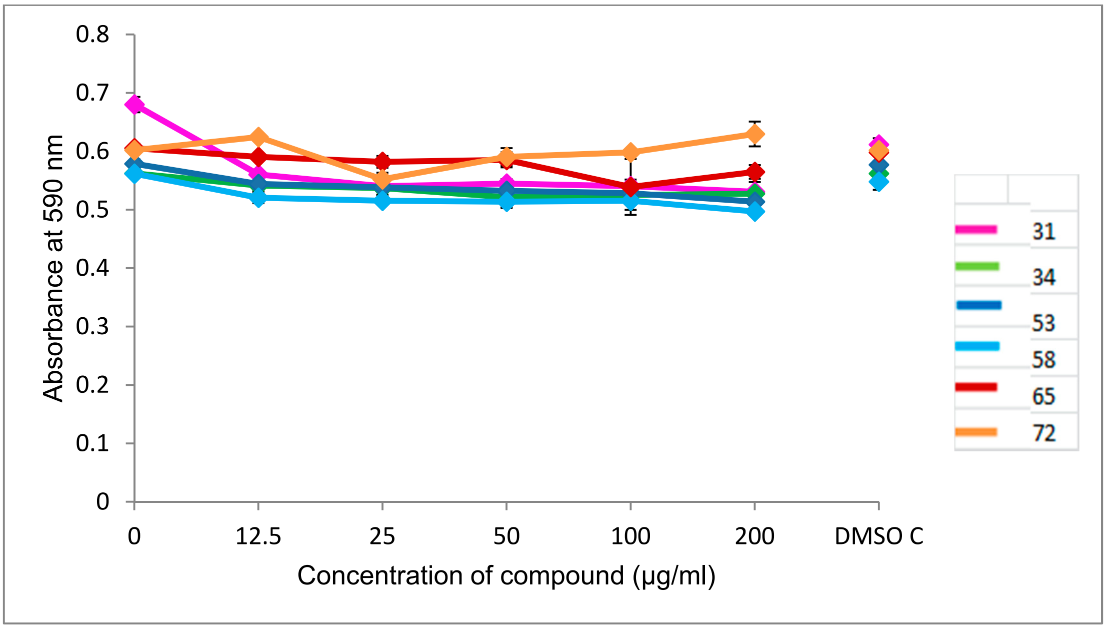

Figure S6. The graph shows the absorbance measured at the 590 nm wave-length after 16 h of incubation for *Proteus vulgaris* with different concentrations of compounds **31**, **34**, **53**, **58**, **65** and **72** ranging from 12.5 µg/mL to 200 µg/mL. There is no decrease in absorbance for any of the compounds, meaning there is no inhibition of growth. At the end is the DMSO control.

Table S1. MIC and MBC (μmol/mL) of selected compounds and Streptomycin against Gram-positive bacteria *B. subtilis*, *S. aureus* and Gram-negative bacteria *P. vulgaris*, *S. typhimurium*.

Compound	*B. subtilis*		*S. aureus*		*P. vulgaris*		*S. typhimurium*	
	**MIC**	**MBC**	**MIC**	**MBC**	**MIC**	**MBC**	**MIC**	**MBC**
**31**	996	996	996	>1195	>1195	>1195	>1195	>1195
**33**	113	113	227	>1363	>1363	>1363	>1363	>1363
**38**	108	108	216	>1301	>1301	>1301	>1301	>1301
**43**	49.5	99	198	-	>1188	>1188	>1188	>1188
**45**	938	938	938	-	>1125	>1125	>1125	>1125
**50**	1059	1271	847	>1271	>1271	>1271	>1271	>1271
**53**	216	216	216	433	>1301	>1301	>1301	>1301
**55**	204	204	408	>1226	>1226	>1226	>1226	>1226
**58**	49.5	99	198	396	>1188	>1188	>1188	>1188
**60**	1125	>1125	750	750	>1125	>1125	>1125	>1125
**63**	>1301	>1301	867	>1301	>1301	>1301	>1301	>1301
**68**	>1188	>1188	990	>1188	>1188	>1188	>1188	>1188
**Streptomycin**	21.4	21.4	21.4	42.9	10.7	10.7	171.9	171.9
